# Supplementary material for: State of the art modelling for the Black Sea ecosystem to support European policies
Source: PLoS One. 2025 Jan 15;20(1):e0312170. doi: 10.1371/journal.pone.0312170 (PMC11734964; doi:10.1371/journal.pone.0312170)
Supplement: S1 Appendix — (DOCX) [file pone.0312170.s001.docx]

**Supporting information 1**


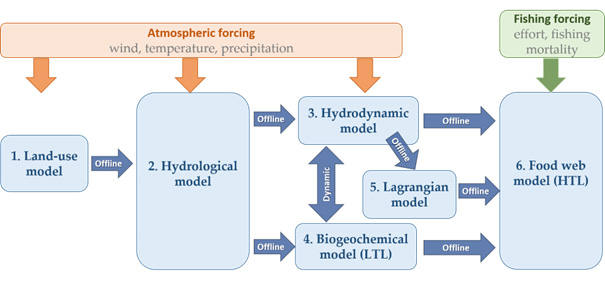


**Fig. 1. Blue2 Modelling Framework.**

**Table 1. Black Sea functional groups source data.**

Species composition, methods, and references used to estimate the basic input parameters (Biomass (B), Production over Biomass (P/B), Consumption over Biomass (Q/B), Biomass accumulation (BA), Diet (D), By-Catch (B-C), Catch (C), Discard (Di) for the 1995 Ecopath model.

| **Biomass references include also time series data incorporated in Ecosim (1995-2021) module. Abbreviations: E (energy required); W (weight); a (coefficient); z (total mortality); F (fishing mortality); M (natural mortality); T (water temperature); A (aspect ratio caudal fin); h (dummy variable= 1 for herbivores and 0 for carnivores and detritivores); d (dummy variable= 1 for detritivores and 0 for herbivores and carnivores); K (growth coefficient); BT (bottom temperature); D (water depth); Me (dummy variable= 1 for motile epifauna and 0 for others); Car (dummy variable= 1 for carnivorous and 0 for others); Mol (dummy variable= 1 for mollusca and 0 for others); Crus (dummy variable= 1 for crustaceans and 0 for others); Pol (dummy variable= 1 for polychaeta and 0 for others); Echi (dummy variable= 1 for echinodermata and 0 for others)** | | |
| --- | --- | --- |
| **Input parameters** | **Method** | **Source** |
| **1. Bottlenose dolphin:** *Tursiops truncatus* | | |
| **B** |  | [1-3](#_ENREF_1) |
| **P/B** | Life history table | [4](#_ENREF_4) |
| **Q/B** | From modified energy requirement equation: E =aW^0.714^ | [5](#_ENREF_5),[6](#_ENREF_6) |
| **D** |  | [7](#_ENREF_7),[8](#_ENREF_8) |
| **B-C** |  | [9-14](#_ENREF_9) |
| **2. Short-beaked common dolphin:** *Delphinus delphis* | | |
| **B** |  | [1-3](#_ENREF_1) |
| **P/B** | Life history table | [4](#_ENREF_4) |
| **Q/B** | From modified energy requirement equation: E =aW^0.714^ | [5](#_ENREF_5),[6](#_ENREF_6) |
| **D** |  | [2](#_ENREF_2),[8](#_ENREF_8) |
| **B-C** |  | [9-14](#_ENREF_9) |
| **3. Harbour porpoise:** *Phocoena phocoena* | | |
| **B** |  | [1](#_ENREF_1) |
| **P/B** | Life history table | [4](#_ENREF_4) |
| **Q/B** | From modified energy requirement equation: E =aW^0.714^ | [5](#_ENREF_5),[6](#_ENREF_6) |
| **D** |  | [15-19](#_ENREF_15) |
| **B-C** |  | [9-14](#_ENREF_9),[20-23](#_ENREF_20) |
| **4.** **Gull and cormorant:** *Larus genei, Larus ichthyaetus, Larus melanocephalus, Larus michahellis, Larus ridibundus, Microcarbo pygmaeus, Phalacrocorax aristotelis, Phalacrocorax carbo* | | |
| **B** |  | [24](#_ENREF_24),[25](#_ENREF_25) |
| **P/B** | Mortality (z)= -ln(Survival rate) | [26-28](#_ENREF_26) |
| **Q/B** | Yearly food intake (including breeding and no breeding seasons)/B | [29](#_ENREF_29),[30](#_ENREF_30) |
| **D** |  | [31-37](#_ENREF_31) |
| **B-C** |  | [9](#_ENREF_9),[11](#_ENREF_11),[38](#_ENREF_38) |
| **5.** **Tern:** *Sterna albifrons, Sterna caspia, Sterna hirundo, Sterna niger, Sterna nilotica, Sterna sandvicensis* | | |
| **B** |  | [24](#_ENREF_24),[25](#_ENREF_25) |
| **P/B** | Mortality (z)= -ln(Survival rate) | [26](#_ENREF_26) |
| **Q/B** | Yearly food intake (including breeding and no breeding seasons)/B | [29](#_ENREF_29),[30](#_ENREF_30) |
| **D** |  | [39](#_ENREF_39) |
| **6. Pelicans:** *Pelecanus crispus, Pelecanus onocrotalus* | | |
| **B** |  | [24](#_ENREF_24),[25](#_ENREF_25) |
| **P/B** | Mortality (z)= -ln(Survival rate) | [*40-42*](#_ENREF_40) |
| **Q/B** | Yearly food intake (including breeding and no breeding seasons)/B | [*43*](#_ENREF_43) |
| **D** |  | [*41*](#_ENREF_41)*,*[*44*](#_ENREF_44) |
| **7. Other large pelagic fish:** *Lichia amia* | | |
| **B** |  | CPUE |
| **P/B** | Mortality (z)= F+M | [45](#_ENREF_45) |
| **Q/B** | log (𝑄/𝐵) = 7.964 − 0.204 𝑙𝑜𝑔𝑊 − 1.965 𝑇 + 0.083 𝐴 + 0.532 ℎ + 0.398 𝑑 | [46](#_ENREF_46) |
| **BA** | To account for migration | [47](#_ENREF_47),[48](#_ENREF_48) |
| **D** |  | [49](#_ENREF_49) |
| **C** |  | GFCM capture production |
| **Di** |  | [50-57](#_ENREF_50) |
| **8. Atlantic bonito:** *Sarda sarda* | | |
| **B** |  | [48](#_ENREF_48) |
| **P/B** | Mortality (z)= F+M | [45](#_ENREF_45) |
| **Q/B** | log (𝑄/𝐵) = 7.964 − 0.204 𝑙𝑜𝑔𝑊 − 1.965 𝑇 + 0.083 𝐴 + 0.532 ℎ + 0.398 𝑑 | [46](#_ENREF_46) |
| **BA** | To account for migration | [47](#_ENREF_47),[48](#_ENREF_48) |
| **D** |  | [58](#_ENREF_58),[59](#_ENREF_59) |
| **C** |  | GFCM capture production |
| **Di** |  | [50-57](#_ENREF_50),[60](#_ENREF_60) |
| **9. Bluefish:** *Pomatomus saltatrix* | | |
| **B** |  | [48](#_ENREF_48) |
| **P/B** | Mortality (z)= F+M | [45](#_ENREF_45) |
| **Q/B** | log (𝑄/𝐵) = 7.964 − 0.204 𝑙𝑜𝑔𝑊 − 1.965 𝑇 + 0.083 𝐴 + 0.532 ℎ + 0.398 𝑑 | [46](#_ENREF_46) |
| **BA** | To account for migration | [47](#_ENREF_47),[48](#_ENREF_48) |
| **D** |  | [61-63](#_ENREF_61) |
| **C** |  | GFCM capture production |
| **Di** |  | [50-57](#_ENREF_50),[60](#_ENREF_60) |
| **10.** **Mackerel:** *Scomber scombrus, Scomber colias* | | |
| **B** |  | CPUE, [64](#_ENREF_64) |
| **P/B** | Mortality (z)= F+M | [45](#_ENREF_45) |
| **Q/B** | log (𝑄/𝐵) = 7.964 − 0.204 𝑙𝑜𝑔𝑊 − 1.965 𝑇 + 0.083 𝐴 + 0.532 ℎ + 0.398 𝑑 | [46](#_ENREF_46) |
| **BA** | To account for migration | [47](#_ENREF_47),[48](#_ENREF_48) |
| **D** |  | [65-68](#_ENREF_65) |
| **C** |  | GFCM capture production |
| **Di** |  | [50-57](#_ENREF_50),[60](#_ENREF_60),[69-71](#_ENREF_69) |
| **11.** **Other medium pelagic fish:** *Alosa immaculata, Alosa caspia, Belone belone, Sphyraena sphyraena* | | |
| **B** |  | [CPUE 64](#_ENREF_64) |
| **P/B** | Mortality (z)= F+M | [45](#_ENREF_45) |
| **Q/B** | log (𝑄/𝐵) = 7.964 − 0.204 𝑙𝑜𝑔𝑊 − 1.965 𝑇 + 0.083 𝐴 + 0.532 ℎ + 0.398 𝑑 | [46](#_ENREF_46) |
| **D** |  | [67](#_ENREF_67),[72](#_ENREF_72),[73](#_ENREF_73) |
| **C** |  | GFCM capture production |
| **Di** |  | [50-57](#_ENREF_50),[60](#_ENREF_60),[69-71](#_ENREF_69) |
| **12.** **Horse mackerel:** *Trachurus trachurus, Trachurus mediterraneus* | | |
| **B** |  | GFCM-stock assessment |
| **P/B** | Mortality (z)= F+M | [45](#_ENREF_45) |
| **Q/B** | log (𝑄/𝐵) = 7.964 − 0.204 𝑙𝑜𝑔𝑊 − 1.965 𝑇 + 0.083 𝐴 + 0.532 ℎ + 0.398 𝑑 | [46](#_ENREF_46) |
| **D** |  | [65-67](#_ENREF_65),[74-77](#_ENREF_74) |
| **C** |  | GFCM capture production |
| **Di** |  | [50-57](#_ENREF_50),[60](#_ENREF_60),[69-71](#_ENREF_69) |
| **13-14.** **European anchovy:** *Engraulis encrasicolus* (Adults&Juveniles) | | |
| **B** |  | GFCM-stock assessment |
| **P/B** | Mortality (z)= F+M | [45](#_ENREF_45) |
| **Q/B** | log (𝑄/𝐵) = 7.964 − 0.204 𝑙𝑜𝑔𝑊 − 1.965 𝑇 + 0.083 𝐴 + 0.532 ℎ + 0.398 𝑑 | [46](#_ENREF_46) |
| **D** |  | [78-81](#_ENREF_78) |
| **C** |  | GFCM capture production |
| **Di** |  | [50-57](#_ENREF_50),[60](#_ENREF_60),[69-71](#_ENREF_69) |
| **15.** **Sprat:** *Sprattus sprattus* | | |
| **B** |  | GFCM-stock assessment |
| **P/B** | Mortality (z)= F+M | [45](#_ENREF_45) |
| **Q/B** | log (𝑄/𝐵) = 7.964 − 0.204 𝑙𝑜𝑔𝑊 − 1.965 𝑇 + 0.083 𝐴 + 0.532 ℎ + 0.398 𝑑 | [46](#_ENREF_46) |
| **D** |  | [67](#_ENREF_67),[78](#_ENREF_78),[80](#_ENREF_80),[82](#_ENREF_82),[83](#_ENREF_83) |
| **C** |  | GFCM capture production |
|  |  | [50-57](#_ENREF_50),[60](#_ENREF_60),[69-71](#_ENREF_69) |
| **16.** **European pilchard:** *Sardina pilchardus* | | |
| **B** |  | CPUE |
| **P/B** | Mortality (z)= F+M | [45](#_ENREF_45) |
| **Q/B** | log (𝑄/𝐵) = 7.964 − 0.204 𝑙𝑜𝑔𝑊 − 1.965 𝑇 + 0.083 𝐴 + 0.532 ℎ + 0.398 𝑑 | [46](#_ENREF_46) |
| **D** |  | [67](#_ENREF_67),[78](#_ENREF_78),[84-86](#_ENREF_84) |
| **C** |  | GFCM capture production |
| **Di** |  | [50-57](#_ENREF_50),[60](#_ENREF_60),[69-71](#_ENREF_69) |
| **17.** **Other small pelagic fish:** *Spicara maena, Spicara spp.* | | |
| **B** |  | CPUE |
| **P/B** | Mortality (z)= F+M | [45](#_ENREF_45) |
| **Q/B** | log (𝑄/𝐵) = 7.964 − 0.204 𝑙𝑜𝑔𝑊 − 1.965 𝑇 + 0.083 𝐴 + 0.532 ℎ + 0.398 𝑑 | [46](#_ENREF_46) |
| **D** |  | [67](#_ENREF_67),[78](#_ENREF_78),[87-89](#_ENREF_87) |
| **C** |  | GFCM capture production |
| **Di** |  | [50-57](#_ENREF_50),[60](#_ENREF_60),[69-71](#_ENREF_69) |
| **18.** **Large demersals:** *Conger conger*, *Merluccius merluccius*, *Lophius budegassa, Lophius piscatorius* | | |
| **B** |  | CPUE |
| **P/B** | Mortality (z)= F+M | [45](#_ENREF_45) |
| **Q/B** | log (𝑄/𝐵) = 7.964 − 0.204 𝑙𝑜𝑔𝑊 − 1.965 𝑇 + 0.083 𝐴 + 0.532 ℎ + 0.398 𝑑 | [46](#_ENREF_46) |
| **D** |  | [67](#_ENREF_67),[90-94](#_ENREF_90) |
| **C** |  | GFCM capture production |
| **Di** |  | [50-57](#_ENREF_50),[60](#_ENREF_60),[69-71](#_ENREF_69) |
| **19. Medium demersal and benthopelagic fish:** *Dicentrarchus labrax, Gaidropsarus spp., Sciaena umbra, Umbrina cirrosa, Zeus faber, Phycis blennoides, Micromesistius poutassou, Scorpaena notata, Scorpaena porcus, Gaidropsarus mediterraneus, Ophidion rochei, Dactylopterus volitans, Chelidonichthys cuculus, Chelidonichthys lucerna, Eutrigla gurnardus, Morone saxatilis* | | |
| **B** |  | CPUE |
| **P/B** | Mortality (z)= F+M | [45](#_ENREF_45) |
| **Q/B** | log (𝑄/𝐵) = 7.964 − 0.204 𝑙𝑜𝑔𝑊 − 1.965 𝑇 + 0.083 𝐴 + 0.532 ℎ + 0.398 𝑑 | [46](#_ENREF_46) |
| **D** |  | [67](#_ENREF_67),[91](#_ENREF_91),[95-108](#_ENREF_95) |
| **C** |  | GFCM capture production |
| **Di** |  | [50-57](#_ENREF_50),[60](#_ENREF_60),[69-71](#_ENREF_69) |
| **20.** **Whiting:** *Merlangius merlangus* | | |
| **B** |  | GFCM-stock assessment |
| **P/B** | Mortality (z)= F+M | [45](#_ENREF_45) |
| **Q/B** | log (𝑄/𝐵) = 7.964 − 0.204 𝑙𝑜𝑔𝑊 − 1.965 𝑇 + 0.083 𝐴 + 0.532 ℎ + 0.398 𝑑 | [46](#_ENREF_46) |
| **D** |  | [67](#_ENREF_67),[80](#_ENREF_80),[91](#_ENREF_91),[109-112](#_ENREF_109) |
| **C** |  | GFCM capture production |
| **Di** |  | [50-57](#_ENREF_50),[60](#_ENREF_60),[69-71](#_ENREF_69),[80](#_ENREF_80),[113](#_ENREF_113) |
| **21. Mugilidae:** *Liza saliens, Liza aurata, Mugil cephalus* | | |
| **B** |  | CPUE, [64](#_ENREF_64) |
| **P/B** | Mortality (z)= F+M | [45](#_ENREF_45) |
| **Q/B** | log (𝑄/𝐵) = 7.964 − 0.204 𝑙𝑜𝑔𝑊 − 1.965 𝑇 + 0.083 𝐴 + 0.532 ℎ + 0.398 𝑑 | [46](#_ENREF_46) |
| **D** |  | [67](#_ENREF_67),[91](#_ENREF_91),[114-117](#_ENREF_114) |
| **C** |  | GFCM capture production |
| **Di** |  | [50-57](#_ENREF_50),[60](#_ENREF_60),[69-71](#_ENREF_69) |
| **22.** **Sparidae:** *Boops boops, Dentex macrophthalmus, Pagrus pagrus, Dentex dentex, Diplodus annularis, Diplodus puntazzo, Diplodus sargus, Diplodus vulgaris, Lithognathus mormyrus, Oblada melanura, Pagellus erythrinus, Sarpa salpa, Spondyliosoma cantharus, Sparus aurata* | | |
| **B** |  | CPUE |
| **P/B** | Mortality (z)= F+M | [45](#_ENREF_45) |
| **Q/B** | log (𝑄/𝐵) = 7.964 − 0.204 𝑙𝑜𝑔𝑊 − 1.965 𝑇 + 0.083 𝐴 + 0.532 ℎ + 0.398 𝑑 | [46](#_ENREF_46) |
| **D** |  | [67](#_ENREF_67),[110](#_ENREF_110),[115](#_ENREF_115),[118-128](#_ENREF_118) |
| **C** |  | GFCM capture production |
| **Di** |  | [50-57](#_ENREF_50),[60](#_ENREF_60),[69-71](#_ENREF_69) |
| **23. Turbot:** *Scophthalmus maximus* | | |
| **B** |  | GFCM-stock assessment |
| **P/B** | Mortality (z)= F+M | [45](#_ENREF_45) |
| **Q/B** | log (𝑄/𝐵) = 7.964 − 0.204 𝑙𝑜𝑔𝑊 − 1.965 𝑇 + 0.083 𝐴 + 0.532 ℎ + 0.398 𝑑 | [46](#_ENREF_46) |
| **D** |  | [67](#_ENREF_67),[112](#_ENREF_112),[115](#_ENREF_115),[120](#_ENREF_120),[129](#_ENREF_129) |
| **C** |  | GFCM capture production |
| **Di** |  | [50-57](#_ENREF_50),[60](#_ENREF_60),[69-71](#_ENREF_69) |
| **24.** **Flatfish:** *Scophthalmus rhombus, Platichthys flesus, Solea solea,* *Pegusa nasuta, Arnoglossus kessleri, Arnoglossus laterna, Arnoglossus thori, Buglossidium luteum, Microchirus variegatus* | | |
| **B** |  | CPUE |
| **P/B** | Mortality (z)= F+M | [45](#_ENREF_45) |
| **Q/B** | log (𝑄/𝐵) = 7.964 − 0.204 𝑙𝑜𝑔𝑊 − 1.965 𝑇 + 0.083 𝐴 + 0.532 ℎ + 0.398 𝑑 | [46](#_ENREF_46) |
| **D** |  | [67](#_ENREF_67),[91](#_ENREF_91),[111](#_ENREF_111),[112](#_ENREF_112),[115](#_ENREF_115),[129-132](#_ENREF_129) |
| **C** |  | GFCM capture production |
| **Di** |  | [50-57](#_ENREF_50),[60](#_ENREF_60),[69-71](#_ENREF_69) |
| **25. Red mullet:** *Mullus barbatus* | | |
| **B** |  | GFCM-stock assessment |
| **P/B** | Mortality (z)= F+M | [45](#_ENREF_45) |
| **Q/B** | log (𝑄/𝐵) = 7.964 − 0.204 𝑙𝑜𝑔𝑊 − 1.965 𝑇 + 0.083 𝐴 + 0.532 ℎ + 0.398 𝑑 | [46](#_ENREF_46) |
| **D** |  | [67](#_ENREF_67),[112](#_ENREF_112),[115](#_ENREF_115),[120](#_ENREF_120),[133-138](#_ENREF_133) |
| **C** |  | GFCM capture production |
| **Di** |  | [50-57](#_ENREF_50),[60](#_ENREF_60),[69-71](#_ENREF_69) |
| **26. Surmullet:** *Mullus surmuletus* | | |
| **B** |  | CPUE[64](#_ENREF_64) |
| **P/B** | Mortality (z)= F+M | [45](#_ENREF_45) |
| **Q/B** | log (𝑄/𝐵) = 7.964 − 0.204 𝑙𝑜𝑔𝑊 − 1.965 𝑇 + 0.083 𝐴 + 0.532 ℎ + 0.398 𝑑 | [46](#_ENREF_46) |
| **D** |  | [67](#_ENREF_67),[115](#_ENREF_115),[120](#_ENREF_120),[135](#_ENREF_135) |
| **C** |  | GFCM capture production |
| **Di** |  | [50-57](#_ENREF_50),[60](#_ENREF_60),[69-71](#_ENREF_69) |
| **27. Small demersal:** *Apletodon dentatus,* *Diplecogaster bimaculata, Lepadogaster spp., Mesogobius batrachocephalus, Atherina spp., Callionymus spp., Aphia minuta, Babka gymnotrachelus, Benthophiloides spp., Caspiosoma caspium, Chromogobius quadrivittatus, Gammogobius steinitzi, Gobius spp., Gobidae, Blenniidae* | | |
| **B** |  | CPUE |
| **P/B** | Mortality (z)= F+M | [45](#_ENREF_45) |
| **Q/B** | log (𝑄/𝐵) = 7.964 − 0.204 𝑙𝑜𝑔𝑊 − 1.965 𝑇 + 0.083 𝐴 + 0.532 ℎ + 0.398 𝑑 | [46](#_ENREF_46) |
| **D** |  | [67](#_ENREF_67),[112](#_ENREF_112),[115](#_ENREF_115),[139](#_ENREF_139),[140](#_ENREF_140) |
| **C** |  | GFCM capture production |
| **Di** |  | [50-57](#_ENREF_50),[60](#_ENREF_60),[69-71](#_ENREF_69),[141](#_ENREF_141) |
| **28. Picked dogfish:** *Squalus acanthias* | | |
| **B** |  | GFCM-stock assessment |
| **P/B** | Mortality (z)= F+M | [45](#_ENREF_45) |
| **Q/B** | log (𝑄/𝐵) = 7.964 − 0.204 𝑙𝑜𝑔𝑊 − 1.965 𝑇 + 0.083 𝐴 + 0.532 ℎ + 0.398 𝑑 | [46](#_ENREF_46) |
| **D** |  | [67](#_ENREF_67),[115](#_ENREF_115),[142-145](#_ENREF_142) |
| **C** |  | GFCM capture production |
| **Di** |  | [50](#_ENREF_50),[52-57](#_ENREF_52),[60](#_ENREF_60),[69-71](#_ENREF_69) |
| **29. Demersal sharks:** *Squatina squatina, Mustelus mustelus* | | |
| **B** |  | CPUE |
| **P/B** | Mortality (z)= F+M | [45](#_ENREF_45) |
| **Q/B** | log (𝑄/𝐵) = 7.964 − 0.204 𝑙𝑜𝑔𝑊 − 1.965 𝑇 + 0.083 𝐴 + 0.532 ℎ + 0.398 𝑑 | [46](#_ENREF_46) |
| **D** |  | [67](#_ENREF_67),[115](#_ENREF_115),[145-152](#_ENREF_145) |
| **C** |  | GFCM capture production |
| **Di** |  | [50](#_ENREF_50),[52-57](#_ENREF_52),[60](#_ENREF_60),[69-71](#_ENREF_69) |
| **30. Rays:** *Dasyatis pastinaca, Raja clavata* | | |
| **B** |  | [64](#_ENREF_64) |
| **P/B** | Mortality (z)= F+M | [45](#_ENREF_45) |
| **Q/B** | log (𝑄/𝐵) = 7.964 − 0.204 𝑙𝑜𝑔𝑊 − 1.965 𝑇 + 0.083 𝐴 + 0.532 ℎ + 0.398 𝑑 | [46](#_ENREF_46) |
| **D** |  | [67](#_ENREF_67),[115](#_ENREF_115),[144](#_ENREF_144),[153-157](#_ENREF_153) |
| **C** |  | GFCM capture production |
| **Di** |  | [50](#_ENREF_50),[52-57](#_ENREF_52),[60](#_ENREF_60),[69-71](#_ENREF_69) |
| **31. Benthic cephalopods:** *Octopus vulgaris, Sepia officinalis* | | |
| **B** |  | CPUE |
| **P/B** | log M =- 0.2107-0.0824 log W +0.6757 log K + 0.4627 log T | [158](#_ENREF_158) |
| **Q/B** |  | [159](#_ENREF_159) |
| **D** |  | [160-162](#_ENREF_160) |
| **C** |  | GFCM capture production |
| **Di** |  | [50-57](#_ENREF_50),[60](#_ENREF_60),[69-71](#_ENREF_69) |
| **32. Shrimp/prawns:** *Palaemon adspersus,* *Palaemon spp., Parapeneus longirostris* | | |
| **B** |  | CPUE |
| **P/B** | log(P/B) = 10.154-0.271*LOG(M)-2824.247*(1/(T+273)-0.063*LOG(D+1)+0.13*(Me)+0.076*(Car)-0.311*(Mol)-0.154*(Crus)-0.266*(Pol)-0.472*(Echi) | [163](#_ENREF_163),[164](#_ENREF_164) |
| **Q/B** |  | [159](#_ENREF_159) |
| **D** |  | [165](#_ENREF_165),[166](#_ENREF_166) |
| **C** |  | GFCM capture production |
| **Di** |  | [50-57](#_ENREF_50),[60](#_ENREF_60),[69-71](#_ENREF_69) |
| **33. Other Decapods:** *Homarus gammarus, Nephrops norvegicus, Eriphia verrucosa* | | |
| **B** |  | CPUE |
| **P/B** | log(P/B) = 10.154-0.271*LOG(M)-2824.247*(1/(T+273)-0.063*LOG(D+1)+0.13*(Me)+0.076*(Car)-0.311*(Mol)-0.154*(Crus)-0.266*(Pol)-0.472*(Echi) | [163](#_ENREF_163),[164](#_ENREF_164) |
| **Q/B** |  | [159](#_ENREF_159) |
| **D** |  | [167-171](#_ENREF_167) |
| **C** |  | GFCM capture production |
| **Di** |  | [50-57](#_ENREF_50),[60](#_ENREF_60),[69-71](#_ENREF_69) |
| **34. *Rapana venosa*** | | |
| **B** |  | GFCM-stock assessment |
| **P/B** |  | GFSM-stock assessment, 198 |
| **Q/B** |  | 2-3 |
| **D** |  | [172-174](#_ENREF_172) |
| **C** |  | GFCM capture production |
| **Di** |  | [50](#_ENREF_50),[52-57](#_ENREF_52),[60](#_ENREF_60),[69-71](#_ENREF_69),[175](#_ENREF_175) |
| **35. Gastropods:** *Amphibalanus improvisus* | | |
| **B** |  | [176](#_ENREF_176) [177-179](#_ENREF_177) [180-182](#_ENREF_180) [176](#_ENREF_176),[183](#_ENREF_183) [184](#_ENREF_184) |
| **P/B** | log(P/B) = 10.154-0.271*LOG(M)-2824.247*(1/(T+273)-0.063*LOG(D+1)+0.13*(Me)+0.076*(Car)-0.311*(Mol)-0.154*(Crus)-0.266*(Pol)-0.472*(Echi) | [163](#_ENREF_163),[164](#_ENREF_164) |
| **Q/B** |  | [2](#_ENREF_2),[3](#_ENREF_3) |
| **D** |  | [2](#_ENREF_2),[3](#_ENREF_3) |
| **C** |  | GFCM capture production |
| **Di** |  | [50-57](#_ENREF_50),[60](#_ENREF_60),[69-71](#_ENREF_69) |
| **36. Bivalves:** *Mytilus galloprovincialis, Parvicardium exiguum, Papillicardium papillosum, Modiolula phaseolina, Mya arenaria, Chamelea gallina* | | |
| **B** |  | [176](#_ENREF_176),[183](#_ENREF_183) [184-186](#_ENREF_184) [177-179](#_ENREF_177) [180-182](#_ENREF_180) |
| **P/B** | log(P/B)=10.154-0.271*LOG(W)-2824.247*(1/(BT+273)-0.063*LOG(D+1)+0.13*(Me)+0.076*(Car)-0.311*(Mol)-0.154*(Crus)-0.266*(Pol)-0.472*(Echi) | [163](#_ENREF_163),[164](#_ENREF_164) [185](#_ENREF_185) |
| **Q/B** |  | [2](#_ENREF_2),[3](#_ENREF_3) |
| **D** |  | [2](#_ENREF_2),[3](#_ENREF_3) |
| **C** |  | GFCM capture production |
| **Di** |  | [50-57](#_ENREF_50),[60](#_ENREF_60),[69-71](#_ENREF_69),[187](#_ENREF_187) |
| **37. Mobile benthos:** *Prionospio cirrifera, Heteromastus filiformis, Nemertea, Nephtys hombergii, Dipolydora quadrilobata, Pholoe inornata, Spirobranchus triqueter, Harmothoe reticulata, Terebellides stroemii, Phtisica marina* | | |
| **B** |  | [176](#_ENREF_176),[183](#_ENREF_183) [177-182](#_ENREF_177),[184](#_ENREF_184) |
| **P/B** | log(P/B) = 10.154-0.271*LOG(M)-2824.247*(1/(T+273)-0.063*LOG(D+1)+0.13*(Me)+0.076*(Car)-0.311*(Mol)-0.154*(Crus)-0.266*(Pol)-0.472*(Echi) | [163](#_ENREF_163),[164](#_ENREF_164) |
| **Q/B** |  | [159](#_ENREF_159) |
| **D** |  | [2](#_ENREF_2),[3](#_ENREF_3) |
| **Di** |  | [50-57](#_ENREF_50),[60](#_ENREF_60),[69-71](#_ENREF_69),[184](#_ENREF_184),[187](#_ENREF_187) |
| **38. Sessile benthos:** *Cryptosula pallasiana, Ascidiella aspersa* | | |
| **B** |  | [176](#_ENREF_176),[183](#_ENREF_183) [177-182](#_ENREF_177),[184](#_ENREF_184) |
| **P/B** | log(P/B) = 10.154-0.271*LOG(M)-2824.247*(1/(T+273)-0.063*LOG(D+1)+0.13*(Me)+0.076*(Car)-0.311*(Mol)-0.154*(Crus)-0.266*(Pol)-0.472*(Echi) | [163](#_ENREF_163),[164](#_ENREF_164) |
| **Q/B** |  | [159](#_ENREF_159) |
| **D** |  | 2,3 |
| **Di** |  | [50-57](#_ENREF_50),[60](#_ENREF_60),[69-71](#_ENREF_69),[187](#_ENREF_187) |
| **39. Jelly:** *Aurelia aurita, Mnemiopsis leidyi* | | |
| **B** |  | [80](#_ENREF_80),[188](#_ENREF_188) |
| **P/B** |  | [189](#_ENREF_189) |
| **Q/B** |  | [2](#_ENREF_2),[3](#_ENREF_3) |
| **D** |  | [2](#_ENREF_2),[3](#_ENREF_3) |
| **40-41. Small and Large-Zooplankton** | | |
| **B** |  | [190](#_ENREF_190),[191](#_ENREF_191) |
| **P/B** |  | [190](#_ENREF_190),[191](#_ENREF_191) |
| **Q/B** |  | [190](#_ENREF_190),[191](#_ENREF_191) |
| **D** |  | [190](#_ENREF_190),[191](#_ENREF_191) |
| **43-43. Small and large phytoplankton: Dinoflagellates and Diatoms** | | |
| **B** |  | [190](#_ENREF_190) |
| **P/B** |  | [191](#_ENREF_191) |
| **44. Seagrass:** *Zoostera marina, Zoostera noltii* | | |
| **B** |  | [192](#_ENREF_192) [193-196](#_ENREF_193) |
| **P/B** |  | [2](#_ENREF_2),[3](#_ENREF_3) |
| **Di** |  | [2](#_ENREF_2),[3](#_ENREF_3) |
| **45.** **Seaweed:** *Ceramium virgatum, Ceramium diaphanum, Cladophora spp., Ulva rigida, Cystoseira barbata* | | |
| **B** |  | [193](#_ENREF_193),[196](#_ENREF_196),[197](#_ENREF_197) |
| **P/B** |  | [2](#_ENREF_2),[3](#_ENREF_3) |

Fig. S2 shows the selected parameters that define the ecological and thermodynamic rules of the balanced model. We considered the Ecopath model balanced given the following six criteria:

1. Ecotrophic efficiency (EE (A)) represents the production utilized within the system (e.g., predation and fishing mortalities): most functional groups should have EE values close to, but lower than, 1 (Heymans et al., 2016). Homeotherms (green circles) are often top predators that are not fished or predated upon; therefore, low EE is expected for those groups, as for other large pelagics (7). The jellyfish group also showed a low EE value due to a lack of knowledge on their predation mortality despite the fact that this group is far from being a ‘trophic dead-end’ [87], there are little information on their predators as they are quickly digested. EE values for primary producers were also significantly less than 1, as there is no good information regarding macrophyte predators and they have been reported to be not well consumed within the ecosystem [88]. Phytoplankton energy flows outside the microbial loop are underrepresented in food web models [89].
2. The production/consumption rate (P/Q (B)) should range from 0.1 to 0.3 for most functional groups; fast-growing groups such as small and large zooplankton (40, 41) slightly exceeded the highest range, while slow-growing groups such as homeotherms showed lower values. The P/Q value for Atlantic bonito (8) is expected to be in the lowest range, as the vital rates of top predators should be lower than those of their preys [30,45] However, this rate was lower than the lowest range, as well as for anchovy juveniles (14) and rays (30). The input values generated by these P\Q ratios have been double-checked, and better data should be used when available.
3. Respiration/biomass rate (R/B (C)) ranges from 1 to 10 for fishes and higher values for small organisms (homeotherms groups, seabirds with high respiration rates (Heymans et al., 2016) and zooplankton were excluded from this analysis). Results suggested that “mobile benthos” group (37) biomass data should be revised when new data are available, whilst anchovy juvenile (14) are expected to have high respiration rates.
4. The slope of the biomass (on a log scale) (D) should decline by 5–10% across all taxa arrayed by trophic level, and groups that are above or below the slope line should be checked for data integrity (Heymans et al., 2016). Seabirds (4, 5, and 6) do not follow this rule having small individual weight (therefore the group biomasses). The overall slope for this model was ~13%, which is slightly above the range suggested.
5. The slope of the production/biomass rate (on a log scale) (P/B (E)) across all taxa aligned by their trophic levels declined by 9% within the expected range of 5–10%.
6. The slope of the consumption/biomass rate (on a log scale) (Q/B (F)) across all taxa aligned by their trophic levels declined by 3% below the expected range of 5–10%. This is due to the high expected Q/B rates of seabirds located at mid-high TL (Heymans et al., 2016).

**
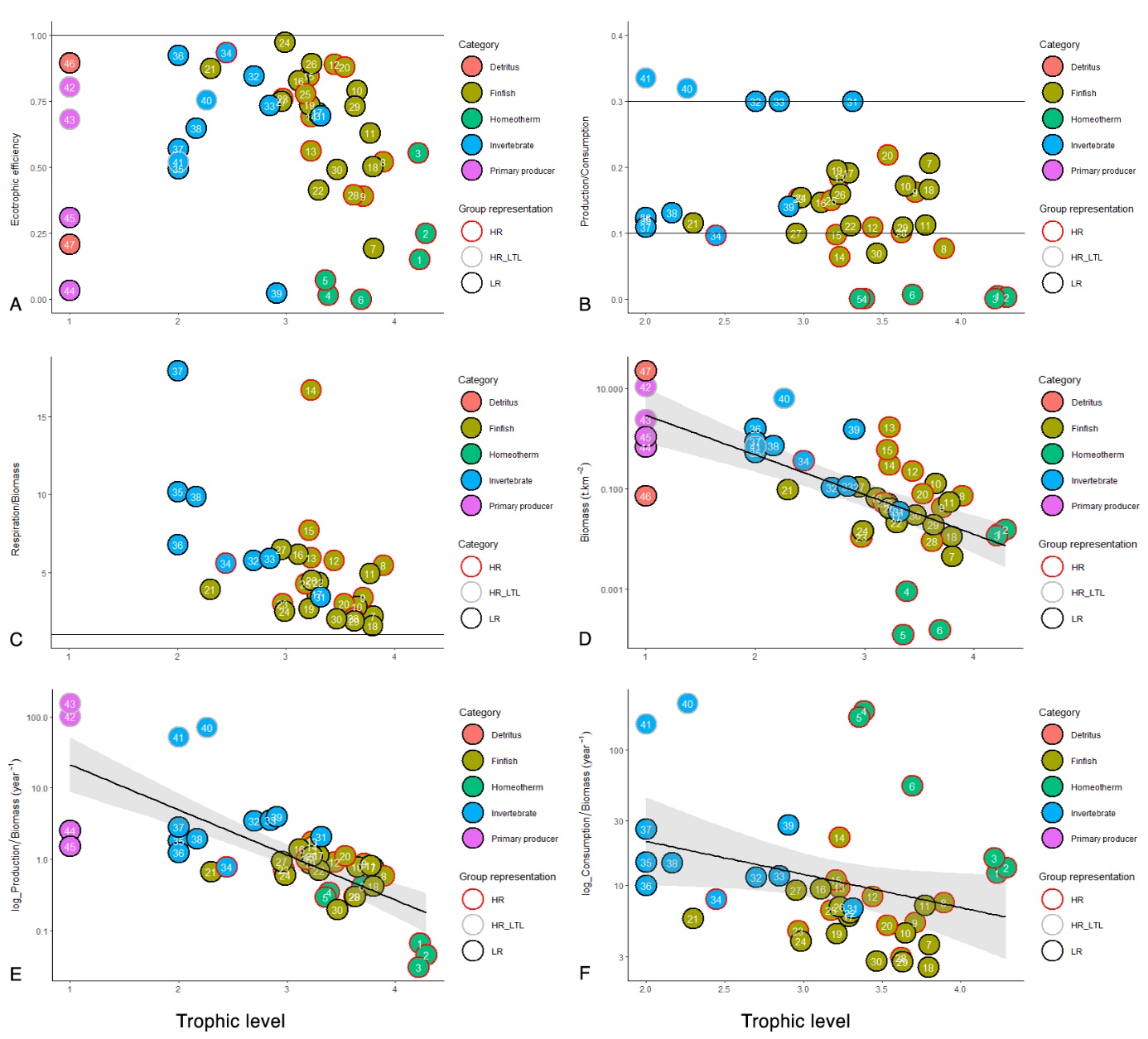
Fig. 2. Selected parameters versus trophic levels defining balancing rules.**

Ecotrophic efficiency (A), Production/consumption rate (B), Respiration/biomass rate (C), Biomass (on logarithmic scale) (D), Production/biomass rate (E), Consumption/biomass rate (F).

**Table 2. Functional groups basal dispersal rates.**

| Functional group | Base dispersal rate | Functional group | Base dispersal rate |
| --- | --- | --- | --- |
| 1_Bottlenose_dolphin | 500 | 24_Flatfish | 30 |
| 2_Common_dolphin | 500 | 25_Red_mullet | 30 |
| 3_Harbour_porpoise | 500 | 26_Striped_mullet | 30 |
| 4_Seagulls_Cormorants | 300 | 27_Small_demersal | 30 |
| 5_Terns | 300 | 28_Picked_dogfish | 30 |
| 6_Pelicans | 300 | 29_Demersal sharks | 30 |
| 7_Other_large_pelagic_fish | 500 | 30_Rays | 30 |
| 8_Atlantic_bonito | 500 | 31_Benthic_cephalapods | 10 |
| 9_Bluefish | 300 | 32_Shrimp/prawns | 10 |
| 10_Mackerels | 100 | 33_Other_decapods | 3 |
| 11_Medium_pelagics | 100 | 34_Rapana_venosa | 3 |
| 12_Horse_mackerel | 50 | 35_Gastropoda | 3 |
| 13_Anchovy_Ad. | 50 | 36_Bivalvia | 3 |
| 14_Anchovy_Juv. | 50 | 37_Mobile_benthos | 5 |
| 15_Sprat | 50 | 38_Sessile_benthos | 3 |
| 16_European_pilchard | 50 | 39_Jellyfish | 50 |
| 17_Other_small_pelagic_fish | 50 | 40_Zoo_large | 100 |
| 18_Large_demersals | 30 | 41_Zoo_small | 100 |
| 19_Med_dem/ben_pel_fish | 30 | 42_Phy_large | 100 |
| 20_Whitin | 30 | 43_Phy_small | 100 |
| 21_Mugilidae | 30 | 44_Seagrass | 1 |
| 22_Sparidae | 30 | 45_Seaweed | 1 |
| 23_Turbot | 30 | 46_Discards | 1 |

**Table 3. Functional groups response functions to environmental variables.**

Showing the niche preferences (minimum, 10^th^ and 90^th^ percentiles, and maximum) for depth, temperature, salinity and oxygen.

|  | DEPTH | | | | TEMPERATURE | | | | SALINITY | | | | OXYGEN | |
| --- | --- | --- | --- | --- | --- | --- | --- | --- | --- | --- | --- | --- | --- | --- |
| FGs | Min | Pref  Min  (10th) | Pref  Max  (90th) | Max | Min | Pref Min (10th) | Pref Max (90th) | Max | Min | Pref Min (10th) | Pref Max (90th) | Max | Min | Pref Min (10th) |
| 1_Bottlenose_dolphin | **8** | **50** | **220** | **2170** |  |  |  |  |  |  |  |  |  |  |
| 2_Common_dolphin | **8** | **290** | **1550** | **2212** |  |  |  |  |  |  |  |  |  |  |
| 3_Harbour_porpoise | **0** | **10** | **50** | **2000** |  |  |  |  |  |  |  |  |  |  |
| 4_Seagulls_Cormorants | **0** | **0** | **100** | **500** |  |  |  |  |  |  |  |  |  |  |
| 5_Terns | **0** | **0** | **40** | **150** |  |  |  |  |  |  |  |  |  |  |
| 6_Pelicans | **0** | **0** | **40** | **150** |  |  |  |  |  |  |  |  |  |  |
| 7_Other_large_pelagic_fish | **8** | **49** | **82.4** | **2212** | 4 | 15 | 28 | 32 | 8 | 32 | 37 | 39 |  |  |
| 8_Atlantic_bonito | **40** | **92** | **139** | **2212** | 3 | 13 | 28 | 32 | 5 | 31 | 37 | 40 |  |  |
| 9_Bluefish | **8** | **22** | **95** | **2212** | 4 | 14 | 27 | 32 | 13 | 30 | 37 | 41 |  |  |
| 10_Mackerels | **8** | **50** | **200** | **1000** | 3 | 12 | 28 | 32 | 24 | 32 | 35 | 36 |  |  |
| 11_Medium_pelagics | **20** | **56** | **174** | **1000** | 9 | 13 | 22 | 28 | 9 | 14 | 38 | 40 |  |  |
| 12_Horse_mackerel | **8** | **61** | **221** | **2212** | 7 | 13 | 20 | 26 | 9 | 32 | 38 | 39 |  |  |
| 13_Anchovy_Ad. | **8** | **10** | **300** | **2212** | 7 | 10 | 26 | 31 | 7 | 7 | 38 | 41 |  |  |
| 14_Anchovy_Juv. | **8** | **10** | **300** | **2212** | 7 | 10 | 26 | 31 | 7 | 7 | 38 | 41 |  |  |
| 15_Sprat | **8** | **10** | **78** | **2212** | 5 | 7 | 18 | 23 | 3 | 6 | 37 | 40 |  |  |
| 16_European_pilchard | **8** | **25** | **100** | **2212** | -1 | 10 | 20 | 25 | 5 | 33 | 38 | 39 |  |  |
| 17_Other_small_pelagic_fish | **8** | **40** | **80** | **2212** | 15 | 18 | 22 | 23 | 16 | 35 | 38 | 40 |  |  |
| 18_Large_demersals | **8** | **74** | **396** | **1056** | 7 | 10 | 23 | 25 | 7 | 32 | 38 | 39 |  |  |
| 19_Medium_dem/ben_pel_fish | **10** | **38** | **114** | **2212** | 9 | 12 | 23 | 30 | 11 | 31 | 38 | 39 |  |  |
| 20_Whiting | **8** | **30** | **50** | **1000** | 6 | 8 | 19 | 20 | 7 | 17 | 37 | 38 |  |  |
| 21_Mugilidae | **8** | **8** | **10** | **120** | 3 | 16 | 28 | 32 | 16 | 31 | 37 | 39 |  |  |
| 22_Sparidae | **8** | **8** | **67** | **227** | 10 | 16 | 24 | 28 | 15 | 29 | 38 | 40 |  |  |
| 23_Turbot | **20** | **25** | **46** | **70** | 7 | 10 | 19 | 21 | 4 | 7 | 35 | 39 | **0** | **10** |
| 24_Flatfish | **8** | **10** | **59** | **194** | 5 | 10 | 19 | 24 | 7 | 11 | 37 | 40 | **0** | **10** |
| 25_Red_mullet | **10** | **100** | **300** | **328** | 7 | 11 | 22 | 28 | 15 | 32 | 38 | 39 | **0** | **10** |
| 26_Striped_mullet | **8** | **52** | **190** | **409** | -2 | 10 | 21 | 24 | 5 | 31 | 38 | 39 | **0** | **10** |
| 27_Small_demersal | **8** | **8** | **26** | **50** | 12 | 14 | 21 | 25 | 14 | 30 | 39 | 39 |  |  |
| 28_Picked_dogfish | **8** | **50** | **300** | **1099** | -1 | 8 | 19 | 29 | 4 | 31 | 36 | 39 |  |  |
| 29_Demersal sharks | **8** | **8** | **444** | **1099** | 4 | 11 | 22 | 28 | 5 | 33 | 36 | 39 |  |  |
| 30_Rays | **8** | **134** | **444** | **1017** | 1 | 10 | 20 | 28 | 5 | 33 | 38 | 39 |  |  |
| 31_Benthic_cephalapods | **0** | **0** | **50** | **242** | 8 | 11 | 21 | 28 | 31 | 32 | 38 | 39 |  |  |
| 32_Shrimp/prawns | **8** | **11** | **46** | **93** | 8 | 10 | 21 | 24 | 6 | 9 | 38 | 39 |  |  |
| 33_Other_decapods | **8** | **16** | **50** | **150** | 6 | 10 | 18 | 23 | 5 | 32 | 37 | 39 |  |  |
| 34_Rapana_venosa | **10** | **15** | **36** | **60** | 4 | 12 | 22 | 30 | 17 | 28 | 34 | 35 | **0** | **10** |
| 35_Gastropoda | **10** | **15** | **36** | **60** | 4 | 12 | 22 | 30 | 17 | 28 | 34 | 35 | **0** | **10** |
| 36_Bivalvia | **47** | **51** | **67** | **86** | 4 | 11 | 20 | 26 | 12 | 19 | 38 | 39 | **0** | **10** |
| 37_Mobile_benthos | **8** | **10** | **59** | **1000** |  |  |  |  |  |  |  |  | **0** | **10** |
| 38_Sessile_benthos | **8** | **10** | **59** | **1000** |  |  |  |  |  |  |  |  | **0** | **10** |
| 44_Seagrass | **0** | **0** | **20** | **30** |  |  |  |  |  |  |  |  |  |  |
| 45_Seaweed | **0** | **0** | **20** | **30** |  |  |  |  |  |  |  |  |  |  |


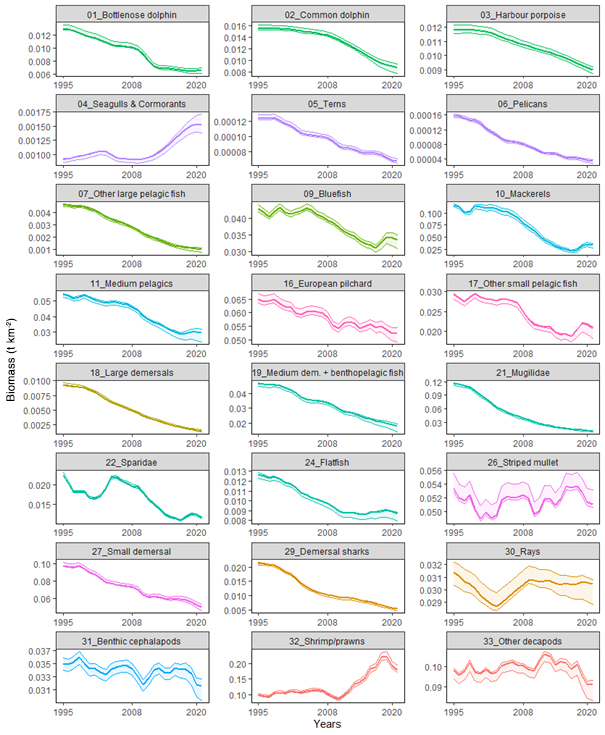


**Fig. 3. Modelled biomass predictions of the best-fitted model.**

Modelled biomass predictions of the best fitted model for those functional groups without observed data for the 1995-2021 period.

**Table 4. Trend analyses.**

Results of predicted biomasses for functional groups with observed time series data. Italics indicate significant trends at α = 0.05.

| **Functional Group** | **Slope** | **Intercept** | **p-value** |
| --- | --- | --- | --- |
| Atlantic bonito | -0.0012 | 0.108 | *<< 0.001* |
| Mediterranean horse mackerel | -0.0014 | 0.207 | *0.035* |
| Anchovy adults | -0.0204 | 2.221 | *0.028* |
| Anchovy juveniles | -0.0025 | 0.119 | 0.564 |
| Sprat | -0.0054 | 0.509 | *0.001* |
| Whiting | -0.0011 | 0.052 | *0.034* |
| Turbot | -0.0001 | 0.008 | *0.045* |
| Red mullet | -0.0002 | 0.045 | *0.001* |
| Picked dogfish | -0.0001 | 0.008 | *<< 0.001* |
| *Rapana venosa* | -0.0035 | 0.365 | *0.003* |


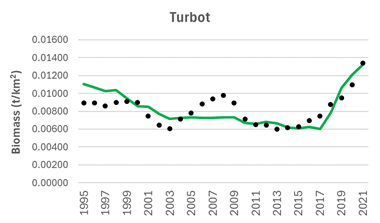


**Fig. 4. Turbot predictions with alternative model parameterization.**


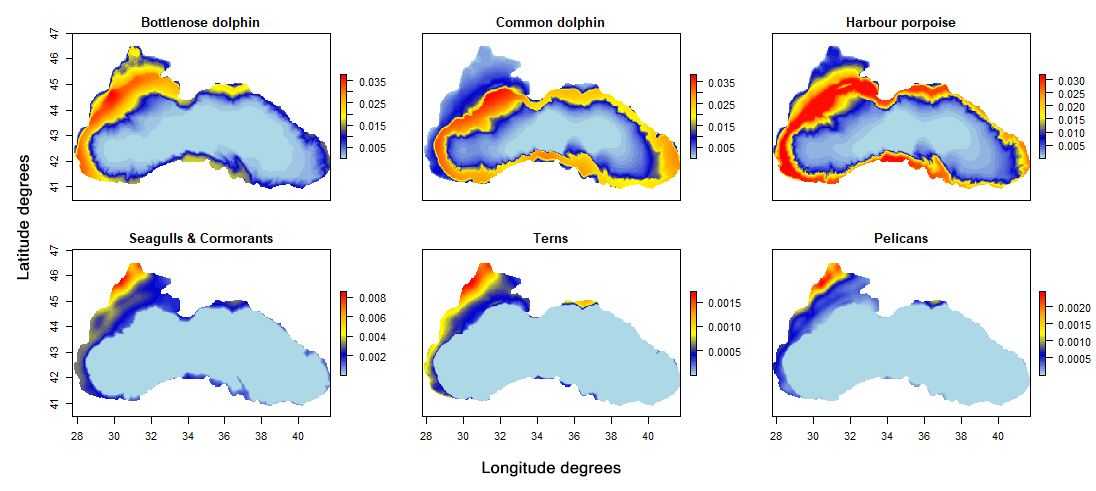
**Fig. 5. Biomass spatial distributions of mammals and seabirds species.**

Data are presented as t*km^-2^ averaged over all modelled years 1995-2021.


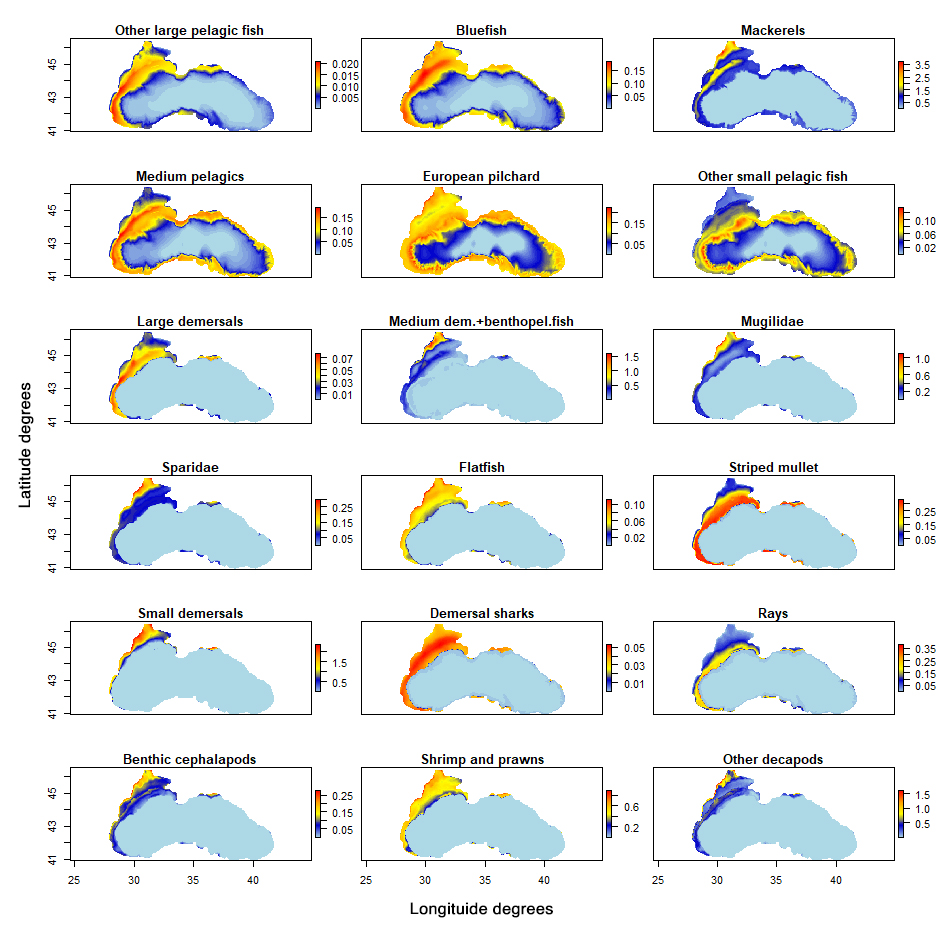


**Fig. 6.** **Biomass spatial distributions of the non-stock-assessed species.**

Data are presented as t*km^-2^ averaged over all modelled years 1995-2021.

References Table 1

1 Paiu, R. M., Panigada, S., Cañadas, A., Gol’din, P., Popov, D., David, L., Amaha Ozturk, A., Glazov, D. ACCOBAMS. Estimates of abundance and distribution of cetaceans in the Black Sea from 2019 surveys. 54 (Monaco, 2021).

2 Piroddi, C. *et al.* Modelling the Mediterranean Sea ecosystem at high spatial resolution to inform the ecosystem-based management in the region. *Scientific Reports* **12**, 19680 (2022).

3 Piroddi, C. *et al.* Historical changes of the Mediterranean Sea ecosystem: modelling the role and impact of primary productivity and fisheries changes over time. *Scientific reports* **7**, 44491 (2017).

4 Barlow, J. & Boveng, P. Modeling age‐specific mortality for marine mammal populations. *Marine Mammal Science* **7**, 50-65 (1991).

5 Pauly, D., Trites, A., Capuli, E. & Christensen, V. Diet composition and trophic levels of marine mammals. *ICES Journal of Marine Science: Journal du Conseil* **55**, 467-481 (1998).

6 Hunter, J. *A multiple regression model for predicting the energy requirements of marine mammals.* MSc degree thesis, University of British Columbia, (2005).

7 Gladilina, E. & Gol’din, P. New prey fishes in diet of black sea bottlenose dolphins, Tursiops truncatus (Mammalia, Cetacea). *Vestnik Zoologii* **48**, 83-92 (2014).

8 Birkun Jr, A. Interactions between cetaceans and fisheries in the Black Sea. *Cetaceans of the Mediterranean and Black Seas: state of knowledge and conservation strategies. A report to the ACCOBAMS Secretariat*, 11 (2002).

9 Ancha, L. Regional by-catch of long-lived species (sea birds, marine mammals and sea turtles) in the Mediterranean and Black Seas. *Masters project to be submitted in partial fulfillment of the requirements for the Master of Environmental Management degree in the Nicholas School of the Environment and Earth Sciences of Duke University* (2008).

10 Reeves, R. R., McClellan, K. & Werner, T. B. Marine mammal bycatch in gillnet and other entangling net fisheries, 1990 to 2011. *Endangered Species Research* **20**, 71-97 (2013).

11 Lewison, R. L. *et al.* Global patterns of marine mammal, seabird, and sea turtle bycatch reveal taxa-specific and cumulative megafauna hotspots. *Proceedings of the National Academy of Sciences* **111**, 5271-5276 (2014).

12 European Commission. Report o the second meeting of the subgroup on fishery and environment (SGFEN) of the Scientific, Technical and Economic Committee for Fisheries (STECF). Incidental catches of small cetaceans. (Brussels, 2002).

13 Zaharieva, Z., Racheva, V. & Simeonovska-Nikolova, D. Cetacean bycatch in turbot gillnets by Bulgarian fisheries in the Black Sea. *Acta Zoolоgica Bulgarica* **74**, 95-102 (2022).

14 Tonay, A. M. Estimates of cetacean by-catch in the turbot fishery on the Turkish Western Black Sea Coast in 2007 and 2008. *Journal of the Marine Biological Association of the United Kingdom* **96**, 993-998 (2016).

15 Tonay, A. M., Dede, A., Öztürk, A.A., Öztürk, B. (ed Rapp. Comm. int. Mer Médit.) (CIESM).

16 Santos, M. & Pierce, G. J. The diet of harbour porpoise (Phocoena phocoena) in the northeast Atlantic: a review. *Oceanography and marine biology*, 363-369 (2003).

17 Andreasen, H. *et al.* Diet composition and food consumption rate of harbor porpoises (Phocoena phocoena) in the western Baltic Sea. *Marine Mammal Science* **33**, 1053-1079 (2017).

18 Gol'din, P. Growth and body size of the harbour porpoise, Phocoena phocoena (Cetacea, Phocoenidae), in the Sea of Azov and the Black Sea. (2004).

19 Bilgin, S., Kose, O. & Yesilcicek, T. External morphology and weight-length relationships (WLRs) of harbour porpoise, Phocoena phocoena (Cetacea: Phocoenidae) in the Black Sea. *Turkish Journal of Fisheries and Aquatic Sciences* **20**, 221-228 (2019).

20 López, D. M., Barcelona, S. G., Báez, J. C., De la Serna, J. M. & de Urbina, J. M. O. Marine mammal bycatch in Spanish Mediterranean large pelagic longline fisheries, with a focus on Risso’s dolphin (Grampus griseus). *Aquatic Living Resources* **25**, 321-331 (2012).

21 Di Natale, A. By-catch of Marine Mammals in Tuna and Swordfish Fisheries: the Mediterranean Case. *ICES CM* (1997).

22 Bilgin, S., Köse, Ö. & Yeşilçiçek, T. Incidental catches of endangered (Phocoena phocoena) and vulnerable (Delphinus delphis) cetaceans and catch composition of turbot bottom gillnet fisheries in the southeastern Black Sea, Turkey. (2018).

23 Popov, D. *et al.* Assessment of the bycatch level for the Black Sea harbour porpoise in the light of new data on population abundance. *Frontiers in Marine Science* **10**, 1119983 (2023).

24 Rudenko, A. G. Present status of gulls and terns nesting in the Black Sea Biosphere Reserve. *Colonial Waterbirds*, 41-45 (1996).

25 Nankinov, D. N. Coastal parks and reserves along the Black Sea and their importance for seabirds. *Marine Ornithology* **24**, 35-38 (1996).

26 Krebs, C. J. *Ecological methodology*. (Harper & Row New York, 1989).

27 Arizaga, J., Herrero, A., Aldalur, A., Cuadrado, J. F. & Oro, D. Effect of Pre-Fledging Body Condition on Juvenile Survival in Yellowlegged Gulls Larus michahellis. *Acta Ornithologica* **50**, 139-147 (2015).

28 Cam, E., Oro, D., Pradel, R. & Jimenez, J. Assessment of hypotheses about dispersal in a long‐lived seabird using multistate capture–recapture models. *Journal of Animal Ecology* **73**, 723-736 (2004).

29 Karpouzi, V. S., Watson, R. & Pauly, D. Modelling and mapping resource overlap between seabirds and fisheries on a global scale: a preliminary assessment. *Marine Ecology Progress Series* **343**, 87-99 (2007).

30 Paleczny, M. *An analysis of temporal and spatial patterns in global seabird abundance during the modern industrial era, 1950-2010, and the relationship between global seabird decline and marine fisheries catch* Master degree thesis, University of British Columbia, (2012).

31 Oro, D., Ruiz, X., Jover, L., Pedrocchi, V. & González-Solís, J. Diet and adult time budgets of Audouin's Gull Larus audouinii in response to changes in commercial fisheries. *Ibis* **139**, 631-637 (1997).

32 Bosch, M., Oro, D., Cantos, F. J. & Zabala, M. Short‐term effects of culling on the ecology and population dynamics of the yellow‐legged gull. *Journal of Applied Ecology* **37**, 369-385 (2000).

33 Goutner, V. in *Mediterranean Marine Avifauna* 431-447 (Springer, 1986).

34 Goutner, V. The diet of Mediterranean Gull (Larus melanocephalus) chicks at fledging. *Journal für Ornithologie* **135**, 193-201 (1994).

35 Xirouchakis, S. M. *et al.* Status and diet of the European Shag (Mediterranean subspecies) Phalacrocorax aristotelis desmarestii in the Libyan Sea (south Crete) during the breeding season. *Marine Ornithology* **45**, 1-9 (2017).

36 Goutner, V., Papakostas, G. & Economidis, P. S. Diet and growth of great cormorant (Phalacrocorax carbo) nestlings in a Mediterranean estuarine environment (Axios Delta, Greece). *Israel Journal of Ecology and Evolution* **43**, 133-148 (1997).

37 BirdLife International. European Red List of Birds. Luxembourg: Office for Official Publications of the European Communities., (Wageningen, 2015).

38 ICES. Report of the Working Group on Seabird Ecology (WGSE). *ICES CM 2008/LRC:05*, 99 pp. (2008).

39 Dies, J. I. & Dies, B. Breeding biology and colony size of Sandwich Tern at L'Albufera de Valencia (Western Mediterranean). *Ardeola* **51**, 431-435 (2004).

40 Crivelli, A., Hatzilacou, D. & Catsadorakis, G. The breeding biology of the Dalmatian Pelican Pelecanus crispus. *Ibis* **140**, 472-481 (1998).

41 Crivelli, A. Action plan for the Dalmatian pelican (Pelecanus crispus) in Europe. *The European Commission and BirdLife International* (1996).

42 Catsadorakis, G. & Crivelli, A. J. Nesting habitat characteristics and breeding performance of Dalmatian Pelicans in Lake Mikri Prespa, NW Greece. *Waterbirds*, 386-393 (2001).

43 Dentressangle, F., Poizat, G. & Crivelli, A. Feeding frequency influences creching age in the Dalmatian pelican, Pelecanus crispus. *Journal of Ornithology* **149**, 431-437 (2008).

44 Albanis, T., Hela, D. & Hatzilakos, D. Organochlorine residues in eggs of Pelecanus crispus and its prey in wetlands of Amvrakikos Gulf, North-western Greece. *Chemosphere* **31**, 4341-4349 (1995).

45 Pauly, D. On the interrelationships between natural mortality, growth parameters, and mean environmental temperature in 175 fish stocks. *Journal du Conseil* **39**, 175-192 (1980).

46 Palomares, M. L. D. & Pauly, D. Predicting food consumption of fish populations as functions of mortality, food type, morphometrics, temperature and salinity. *Marine and freshwater research* **49**, 447-453 (1998).

47 Daskalov, G. M. Overfishing drives a trophic cascade in the Black Sea. *Marine ecology progress series* **225**, 53-63 (2002).

48 Daskalov, G. M., Demirel, N., Ulman, A., Georgieva, Y. & Zengin, M. Stock dynamics and predator–prey effects of Atlantic bonito and bluefish as top predators in the Black Sea. *ICES Journal of Marine Science* **77**, 2995-3005 (2020).

49 Coetzee, D. Stomach content analysis of the leervis, Lichia amia (L.), from the Swartvlei system, southern Cape. *South African Journal of Zoology* **17**, 177-181 (1982).

50 FAO. The State of Mediterranean and Black Sea Fisheries 2022.

. (Food and Agriculture Organization of the United Nations, Rome, 2022).

51 Şahin, C., Ceylan, Y. & Kalaycı, F. Purse seine fishery discards on the Black Sea coasts of Turkey. *Turkish Journal of Fisheries and Aquatic Sciences* **15**, 81-91 (2015).

52 Kalayci, F. & Yeşilçiçek, T. Influence of season, depth and mesh size on the trammel nets catch composition and discard in the Southern Black Sea, Turkey. *Marine Biology Research* **10**, 824-832 (2014).

53 Balik, İ. Effect of depth and season on catch composition and discard rate in gillnet fishery in the south-eastern coast of the Black Sea. *Acta Aquatica Turcica* **16**, 82-93 (2020).

54 Kasapoglu, N. & Duzgunes, E. The common problem in the Black Sea fisheries: By-catch and its effects on the fisheries economy. *Turkish Journal of Fisheries and Aquatic Sciences* **17**, 387-394 (2017).

55 FAO. The State of Mediterranean and Black Fisheries. 134 pp. (Rome, Italy, 2016).

56 FAO. *Fishery Information, Data and Statistics Unit GFCM capture production 1970–2017* 2020).

57 FAO. The State of Mediterranean and Black Sea Fisheries. 172 (Rome, 2018).

58 Fletcher, N., Batjakas, I. & Pierce, G. Diet of the Atlantic bonito *Sarda sarda* (Bloch, 1793) in the Northeast Aegean Sea. *Journal of Applied Ichthyology* **29**, 1030-1035 (2013).

59 Campo, D., Mostarda, E., Castriota, L., Scarabello, M. & Andaloro, F. Feeding habits of the Atlantic bonito, Sarda sarda (Bloch, 1793) in the southern Tyrrhenian sea. *Fisheries Research* **81**, 169-175 (2006).

60 EC. Impact assessment of discard reducing policies. EU Discard Annex. (European Commission. Project: ZF0926_S10, 2011).

61 Habib, B., YANIK, T. & Türker, D. Diet composition of bluefish Pomatomus saltatrix (Linnaeus, 1766) in the Sea of Marmara. *Marine Science and Technology Bulletin* **9**, 46-50 (2020).

62 Georgieva, Y. G. & Daskalov, G. M. Shift in growth of an apex marine predator: bluefish Pomatomus saltatrix (L., 1766)(Perciformes: Potamonidae) in relation to changes in feeding. *Acta Zoologica Bulgarica* **71**, 63-72 (2019).

63 Dhieb, K., Ghorbel, M. & Bouain, A. Regime alimentaire de Pomatomus saltatrix (Teleostei, Pomatomidae) dans le Golfe de Gabes. *Tunisie. Rapp. Comm. int. Mer Médit* **36**, 259 (2001).

64 Prodanov, K. *Environmental management of fish resources in the Black Sea and their rational exploitation*. Vol. 68 (Food & Agriculture Org., 1997).

65 Stergiou, K. *et al.* New fisheries-related data from the Mediterranean Sea. *Mediterranean Marine Science* **15**, 213-224 (2014).

66 Cardona, L., Álvarez de Quevedo, I., Borrell, A. & Aguilar, A. Massive consumption of gelatinous plankton by Mediterranean apex predators. *PloS one* **7**, e31329 (2012).

67 Karachle, P. *Feeding ecology of the most important fish stock in the North Aegean Sea*, Ph. D. Thesis, Aristotle University of Thessaloniki, Department of Biology …, (2008).

68 Sever, T., Bayhan, B., Bilecenoglu, M. & Mavili, S. Diet composition of the juvenile chub mackerel (Scomber japonicus) in the Aegean Sea (Izmir Bay, Turkey). *Journal of Applied Ichthyology* **22**, 145-148 (2006).

69 Yıldız, T. & Karakulak, F. Discards in bottom‐trawl fishery in the western Black Sea (Turkey). *Journal of Applied Ichthyology* **33**, 689-698 (2017).

70 Ceylan, Y., Şahin, C. & Kalayci, F. Bottom trawl fishery discards in the Black Sea coast of Turkey. *Mediterranean Marine Science* **15**, 156-164 (2014).

71 Sağlam, N. E. & Soyer, M. F. Discards in Bottom-trawl Fishery in the South-Eastern Black Sea, Turkey. *Acta Zoologica Bulgarica* **74** (2022).

72 Ceyhan, T., Akyol, O., Sever, T. M. & Kara, A. Diet composition of adult twaite shad (Alosa fallax) in the Aegean Sea (Izmir Bay, Turkey). *Journal of the Marine Biological Association of the United Kingdom* **92**, 601-604 (2012).

73 Kalogirou, S., Mittermayer, F., Pihl, L. & Wennhage, H. Feeding ecology of indigenous and non‐indigenous fish species within the family Sphyraenidae. *Journal of fish biology* **80**, 2528-2548 (2012).

74 Kyrtatos, N. A. Contribution à la connaissance de la nourriture de Trachurus mediterraneus (Steind.) et de son influence sur les chaînes alimentaires de la mer Égée Centrale. *Rapp. Comm. Int. Expl. Sci. Mer Médit* **35**, 452-453 (1998).

75 Salem, M. B. Régime alimentaire de Trachurus trachurus (Linnaeus, 1758) et de T. mediterraneus (Steindachner, 1868),(poissons, téléosteens, carangidae) de la province Atlantic-Méditerranéenne. *Cybium (Paris)* **12**, 247-253 (1988).

76 Yankova, M. H., Raykov, V. S. & Frateva, P. B. Diet composition of horse mackerel, Trachurus mediterraneus ponticus Aleev, 1956 (Osteichthyes: Carangidae) in the Bulgarian Black Sea waters. *Turkish Journal of Fisheries and Aquatic Sciences* **8** (2008).

77 Georgieva, Y. G., Daskalov, G. M., Klayn, S. L., Stefanova, K. B. & Stefanova, E. S. Seasonal diet and feeding strategy of horse mackerel trachurus mediterraneus (Steindachner, 1868)(Perciformes: Carangidae) in the South-Western Black Sea. *Acta Zool. Bulg* **71**, 201-210 (2019).

78 Karachle, P. & Stergiou, K. Feeding and ecomorphology of three clupeoids in the N Aegean Sea. *Mediterranean Marine Science* **15**, 9-26 (2014).

79 Catalan, I. A. *et al.* Growth and feeding patterns of European anchovy (Engraulis encrasicolus) early life stages in the Aegean Sea (NE Mediterranean). *Estuarine, Coastal and Shelf Science* **86**, 299-312 (2010).

80 Shiganova, T. A. & Bulgakova, Y. V. Effects of gelatinous plankton on Black Sea and Sea of Azov fish and their food resources. *ICES Journal of Marine Science* **57**, 641-648 (2000).

81 Mazlum, R. E., Solak, E. & Bilgin, S. Size and seasonal diet variation of European anchovy, Engraulis encrasicolus (Linnaeus, 1758) in the southeast Black Sea. *Cahiers de Biologie Marine* **58**, 251-260 (2017).

82 Bișinicu, E., Harcotă, G.-E., Țoțoiu, A., Timofte, F. & Radu, G. Romanian Black Sea Zooplankton and Its Role in the Diet of Sprattus in 2016-2017. *Revista Cercetări Marine-Revue Recherches Marines-Marine Research Journal* **47**, 185-193 (2017).

83 Bayhan, B. & Sever, T. M. Spring diet and feeding strategy of the European sprat Sprattus sprattus (L., 1758) from the Black Sea coast of Turkey. *Turkish Journal of Agriculture-Food Science and Technology* **3**, 697-700 (2015).

84 Cunha, M. E., Garrido, S. & Pissarra, J. The use of stomach fullness and colour indices to assess Sardina pilchardus feeding. *Journal of the Marine Biological Association of the United Kingdom* **85**, 425-431 (2005).

85 Demirhindi, U. in *Proceedings and Technical Papers of the General Fisheries Council for the Mediterranean.*

86 Sever, T., Bayhan, B. & Taskavak, E. A preliminary study on the feeding regime of European pilchard (Sardina pilchardus Walbaum 1792) in Izmir Bay, Turkey, Eastern Aegean Sea. *NAGA, WorldFish Center Quarterly* **28**, 41-48 (2005).

87 Harchouche, K., Maurin, C. & Zerouali-Khodja, F. Régime alimentaire de Spicara maena (centracanthidae), des eaux algériennes. *Bulletin de la Société zoologique de France* **134**, 125-143 (2009).

88 Mytilineou, C. *Contribution to the Biology of picarel Spicara flexuosa (Raf. 1810), in the Patraikos Gulf (Greece)*, PhD Thesis, University of Athens, (1987).

89 Sever, T. M. Diet composition of the blotched picarel Spicara flexuosa Rafinesque. 1810 (Actinopterygii: Sparidae) from the Aegean Sea. Turkey. *Acta Zoologica Bulgarica* **71**, 581-588 (2019).

90 Negzaoui-Garali, N., Ben Salem, M. & Capape, C. Feeding habits of the black anglerfish, Lophius budegassa (Osteichthyes: Lophiidae), off the Tunisian coast (central Mediterranean). *Cahiers de biologie marine* **49**, 113 (2008).

91 Macpherson, E. Resource partitioning in a Mediterranean demersal fish community. (1981).

92 Tsimenidis, N. C. Contribution to the study of the angler-fishes Lophius budegassa Spinola, 1807 and L. piscatorius L., 1758 in Greek seas. *Institute of Oceanography Fisheries Research (Athens) Special Publication* **4**, 180-190 (1980).

93 Şenbahar, A. & Özaydin, O. Feeding Ecology of Black-bellied Anglerfish Lophius budegassa in the Central Aegean Sea, Turkey. *Journal of Ichthyology*, 1-10 (2021).

94 Froglia, C. in *Atti V Congresso Nazionale della Societá Italiana di Biologia Marina.* 327-341.

95 Madurell, T. & Cartes, J. E. Trophic relationships and food consumption of slope dwelling macrourids from the bathyal Ionian Sea (eastern Mediterranean). *Marine Biology* **148**, 1325-1338 (2006).

96 Bascinar, N. S. & Sağlam, H. Feeding habits of black scorpionfish Scorpaena porcus, in the South-Eastern Black Sea. *Turkish Journal of Fisheries and Aquatic Sciences* **9** (2009).

97 Roşca, I. & Arteni, O. M. Feeding ecology of black scorpionfish (Scorpaena porcus Linnaeus, 1758) from the Romanian Black Sea (Agigea--Eforie Nord area). *Animal Biology & Animal Husbandry* **2** (2010).

98 Aydin, M. & Mazlum, R. E. Feeding ecology of black scorpion fish (Scorpaena porcus Linnaeus, 1758) in SE Black Sea region,(Ordu) Turkey. *Journal of the Marine Biological Association of the United Kingdom* **100**, 435-444 (2020).

99 Aydin, M. & Bengil, E. G. T. Feeding Habit and Length-Weight Relationship, Sciaena umbra Linnaeus, 1758 from Southeastern Black Sea. *Acta Aquatica Turcica* **16**, 479-486 (2020).

100 Engin, S. & Seyhan, K. Age, growth, sexual maturity and food composition of Sciaena umbra in the south‐eastern Black Sea, Turkey. *Journal of Applied Ichthyology* **25**, 96-99 (2009).

101 Stergiou, K. & Fourtouni, H. Food habits, ontogenetic diet shift and selectivity in Zeus faber Linnaeus, 1758. *Journal of Fish Biology* **39**, 589-603 (1991).

102 Tamoikin, I. Y., Kutsyn, D. & Vdodovich, I. New data on the age, size and feeding of the shi drum Umbrina cirrosa (Sciaenidae) off the Black Sea Coast of Crimea. *Journal of Ichthyology* **63**, 707-717 (2023).

103 Amorim, M. C. P. & Hawkins, A. D. Ontogeny of acoustic and feeding behaviour in the grey gurnard, Eutrigla gurnardus. *Ethology* **111**, 255-269 (2005).

104 Shideler, A. C. & Houde, E. D. Spatio-temporal variability in larval-stage feeding and nutritional sources as factors influencing striped bass (Morone saxatilis) recruitment success. *Estuaries and coasts* **37**, 561-575 (2014).

105 Stagioni, M., Montanini, S. & Vallisneri, M. Feeding of tub gurnard Chelidonichthys lucerna (Scorpaeniformes: Triglidae) in the north-east Mediterranean. *Journal of the Marine Biological Association of the United Kingdom* **92**, 605-612 (2012).

106 Rogdakis, Y., Ramfos, A., Koukou, K., Dimitriou, E. & Katselis, G. Feeding habits and trophic level of sea bass (Dicentrarchus labrax) in the Messolonghi-Etoliko lagoons complex (Western Greece). *Journal of Biological Research* **13**, 13 (2010).

107 Madurell, T. & Labropoulou, M. in *Proceedings of the 6th Panhellenic Symposium of Oceanography and Fisheries.* 39-44.

108 Froglia, C. Osservazioni sull'alimentazione di Sciaena umbra ed Umbrina cirrosa (Pisces, Sciaenidae) in prossimita di barriere artificiali in Adriatico. *Biol. Mar. Med.* **5**, 100-108 (1998).

109 Artüz, M. L. The diet and food consumption of whiting Merlangius merlangus merlangus (Linne) 1758 in the Sea of Marmara. *Hidrobiologica, Publications Scientifiques* **1**, 23-26 (2005).

110 Papaconstantinou, C. *et al.* in *Technical Report* (National Centre for Marine Research Athens, 1989).

111 Papaconstantinou, C. Investigation of the abundance and distribution of the demersal stocks of primary importance to the Greek Fishery in the North Aegean Sea (Greece). (2016).

112 Bănaru, D. & Harmelin-Vivien, M. Feeding behaviour of Black Sea bottom fishes: Did it change over time? *Acta oecologica* **35**, 769-777 (2009).

113 Kalaycı, F. & Yeşilçiçek, T. Effects of depth, season and mesh size on the catch and discards of whiting (Merlangius merlangus euxinus) gillnet fishery in the southern Black Sea, Turkey. *Turkish Journal of Fisheries and Aquatic Sciences* **14**, 449-456 (2014).

114 Labropoulou, M. & Plaitis, W. Selective predation on small crustaceans by six demersal fish species in Iraklion Bay (Cretan Sea, north-eastern Mediterranean). *Oceanographic Literature Review* **2**, 120 (1997).

115 Karachle, P. & Stergiou, K. An update on the feeding habits of fish in the Mediterranean Sea (2002-2015). *Mediterranean Marine Science* **18**, 43-52 (2017).

116 Harmelin‐Vivien, M., Kaim‐Malka, R., Ledoyer, M. & Jacob‐Abraham, S. Food partitioning among scorpaenid fishes in Mediterranean seagrass beds. *Journal of fish biology* **34**, 715-734 (1989).

117 Labropoulou, M. & Eleftheriou, A. The foraging ecology of two pairs of congeneric demersal fish species: importance of morphological characteristics in prey selection. *Journal of fish biology* **50**, 324-340 (1997).

118 Bayhan, B., Sever, T. M. & Heral, O. Diet composition of the Morocco dentex: Dentex maroccanus Valenciennes, 1830 (Teleostei: Sparidae) in the central Turkish Aegean Sea. *Oceanological and Hydrobiological Studies* **46**, 133-139 (2017).

119 Kallianiotis, A., Torre, M. & Argyri, A. Age, growth, mortality, reproduction and feeding habits of the striped seabream, Lithognathus mormyrus (Pisces: Sparidae) in the coastal waters of the Thracian Sea, Greece. *Scientia Marina* **69**, 391-404 (2005).

120 Labropoulou, M. & Papadopoulou-Smith, K.-N. Foraging behaviour patterns of four sympatric demersal fishes. *Estuarine, Coastal and Shelf Science* **49**, 99-108 (1999).

121 Daban, İ. B. Comparative study on the feeding ecology of the White Seabream, Diplodus sargus, and the Black Seabream, Spondyliosoma cantharus (Osteichthyes: Sparidae) in the North Aegean Sea. *Zoology in the Middle East* **68**, 126-134 (2022).

122 Ilhan, D. Age, growth, and diet of axillary seabream, Pagellus acarne (Actinopterygii: Perciformes: Sparidae), in the central Aegean Sea. *Acta Ichthyologica et Piscatoria* **48**, 329-339 (2018).

123 Caragitsou, E. & Papaconstantinou, C. Feeding habits of red pandora (Pagellus erythrinus) off the western coast of Greece. *Journal of Applied Ichthyology* **4**, 14-22 (1988).

124 Jukic, S. Nutrition of the hake (Merluccius merluccius), bogue (Boops boops), striped mullet (Mullus barbatus) and pandora (Pagellus erythrinus) in the Bay of Kaštela. *Acta Adriatica* **14**, 3-40 (1972).

125 Papaconstantinou, C. & Caragitsou, E. Feeding interaction between two sympatric species Pagrus pagrus and Phycis phycis around Kastellorizo Island (Dodecanese, Greece). *Fisheries Research* **7**, 329-342 (1989).

126 Pallaoro, A., Santic, M. & Jardas, I. Feeding habits of the saddled bream, Oblada melanura (Sparidae), in the Adriatic Sea. *Cybium* **27**, 261-268 (2003).

127 Verlaque, M. Note preliminaire sur le comportement alimentaire de Sarpa salpa (L.)(Sparidae) en Méditerranée. *Rapp. Comm. Int. Mer Médit* **29**, 193-196 (1985).

128 Rosecchi, E. L'alimentation de Diplodus annualris, Diplodus sargus, Diplodus vulgaris et Sparus aurata (Pisces, Sparidae) dnas le golfe du Lion et les lagunes littorales. *Revue des Travaux de l'Institut des Pêches maritimes* **49**, 125-141 (1985).

129 Wyche, C. & Shackley, S. The feeding ecology of Pleuronectes platessa L., Limanda limanda (L.) and Scophthalmus rhombus (L.) in Carmarthen Bay, South Wales, UK. *Journal of fish biology* **29**, 303-311 (1986).

130 Bayhan, B., Sever, T. M. & Taşkavak, E. Age, length-weight relationships and diet composition of scaldfish Arnoglossus laterna (Walbaum, 1792)(Pisces: Bothidae) in Izmir Bay (Aegean Sea). *Journal of Animal and Veterinary Advances* **7**, 924-929 (2008).

131 Cresson, P., Ruitton, S., Ourgaud, M. & Harmelin-Vivien, M. Contrasting perception of fish trophic level from stomach content and stable isotope analyses: a Mediterranean artificial reef experience. *Journal of Experimental Marine Biology and Ecology* **452**, 54-62 (2014).

132 Molinero, A. & Flos, R. Influence of sex and age on the feeding habits of the common sole Solea solea. *Marine biology* **111**, 493-501 (1991).

133 Caragitsou, Ε. & Tsimenidis, N. Seasonal changes and comparative analysis of the food of the red mullet (Mullus Barbatus) in the Gulfs of Saronikos and Thermaikos. (2016).

134 Chérif, M. *et al.* Food and feeding habits of the red mullet, Mullus barbatus (Actinopterygii: Perciformes: Mullidae), off the northern Tunisian coast (central Mediterranean). *Acta Ichthyologica et Piscatoria* **41** (2011).

135 Karachle, P. *et al.* New fisheries-related data from the Mediterranean Sea (April 2015). *Mediterranean Marine Science* **16**, 285-293 (2015).

136 Papaconstantinou, C. & Caragitsou, E. in *Proceedings of the 2nd Panhellenic Symposium of Oceanography and Fisheries.* 577-583.

137 Vassilopoulou, V. & Papaconstantinou, C. Feeding habits of red mullet (Mullus barbatus) in a gulf in western Greece. *Fisheries research* **16**, 69-83 (1993).

138 Onay, H. & Dalgic, G. Seasonal changes in the food spectrum and day-time rhythm of feeding in red mullet Mullus barbatus (Linnaeus, 1758) in the southeast Black Sea. *Fresenius Environmental Bulletin* **28**, 2671-2678 (2019).

139 La Mesa, M. *et al.* Feeding ecology of the transparent goby Aphia minuta (Pisces, Gobiidae) in the northwestern Adriatic Sea. *Scientia Marina* **72**, 99-108 (2008).

140 Danilova, M. Diet of juvenile silversides, Atherina boyeri, from the Black Sea. *Journal of ichthyology* **31**, 137-145 (1991).

141 Kasapoğlu, N. Age, growth and mortality rates of discard species (Uranoscopus scaber, Neogobius melanostomus and Gobius niger) in the Black Sea. *Ege Journal of Fisheries and Aquatic Sciences* **33**, 397-403 (2016).

142 Laptikhovsky, V., Arkhipkin, A. & Henderson, A. Feeding habits and dietary overlap in spiny dogfish Squalus acanthias (Squalidae) and narrowmouth catshark Schroederichthys bivius (Scyliorhinidae). *Journal of the Marine Biological Association of the United Kingdom* **81**, 1015-1018 (2001).

143 Avsar, D. Age, growth, reproduction and feeding of the spurdog (Squalus acanthias Linnaeus, 1758) in the South-eastern Black Sea. *Estuarine, Coastal and Shelf Science* **52**, 269-278 (2001).

144 Demirhan, S. A., Seyhan, K. & Başusta, N. Dietary overlap in spiny dogfish (Squalus acanthias) and thornback ray (Raja clavata) in the southeastern Black Sea. (2007).

145 Gül, G. & Demirel, N. Evaluation of the comprehensive feeding strategy and trophic role of overexploited mesopredator species in the Sea of Marmara (northeastern Mediterranean). *Estuarine, Coastal and Shelf Science* **259**, 107448 (2021).

146 Saidi, B., Enajjar, S., Bradai, M. N. & Bouain, A. Diet composition of smooth‐hound shark, Mustelus mustelus (Linnaeus, 1758), in the Gulf of Gabès, southern Tunisia. *Journal of Applied Ichthyology* **25**, 113-118 (2009).

147 Gračan, R., Mladineo, I. & Lazar, B. Insight into the diet composition and gastrointestinal parasite community of the common smooth-hound, Mustelus mustelus (Carcharhiniformes: Triakidae), in the northern Adriatic Sea. *Natura Croatica* **23** (2014).

148 Lipej, L., Mavric, B., Rešek, S., Cherif, M. & Capape, C. Food and feeding habits of the blackspotted smooth-hound, Mustelus punctulatus (Elasmobranchii: Carcharhiniformes: Triakidae), from the northern Adriatic. *Acta Ichthyologica et Piscatoria* **41** (2011).

149 Valls, M., Quetglas, A., Ordines, F. & Moranta, J. Feeding ecology of demersal elasmobranchs from the shelf and slope off the Balearic Sea (western Mediterranean). *Scientia Marina* **75**, 633-639 (2011).

150 Jardas, I. Supplement to the knowledge of ecology of some Adriatic cartilaginous fishes (Chondrichthyes) with special reference to their nutrition. *Acta Adriatica* **14**, 1-60 (1972).

151 Jardas, I., Šantić, M., Nerlović, V. & Pallaoro, A. Diet of the smooth-hound Mustelus mustelus (Chondrichthyes: Triakidae) in the eastern Adriatic Sea. *Cybium* **31**, 459-464 (2007).

152 Filiz, H. Diet composition of smooth-hound, Mustelus mustelus (Linnaeus, 1758), in Aegean Sea, Turkey. *Belg. J. Zool* **139**, 81-84 (2009).

153 Ismen, A. Age, growth, reproduction and food of common stingray (Dasyatis pastinaca L., 1758) in Iskenderun Bay, the eastern Mediterranean. *Fisheries Research* **60**, 169-176 (2003).

154 Saglam, H. & Bascinar, N. S. Feeding ecology of thornback ray (Raja clavata Linnaeus, 1758) on the Turkish coast of the south-eastern Black Sea. *Marine Biology Research* **4**, 451-457 (2008).

155 Santic, M., Paladin, A. & Agovic, A. Diet of common stingray, Dasyatis pastinaca (Chondrichthyes: Dasyatidae) in the eastern Adriatic Sea. *Cahiers de biologie marine* **52**, 349-356 (2011).

156 Šantić, M., Rađa, B. & Pallaoro, A. Diet and feeding strategy of thornback ray Raja clavata. *Journal of fish Biology* **81**, 1070-1084 (2012).

157 Eronat, E. G. T. & Özaydın, O. Diet composition of the thornback ray, Raja clavata Linnaeus, 1758 (Elasmobranchii: Rajidae) in the Turkish Aegean Sea. *Zoology in the Middle East* **61**, 38-44 (2015).

158 Pauly, D. Population dynamics of short-lived species, with emphasis on squids. *Northwest Atlantic Fisheries Organization (NAFO)* **9**, 101-106 (1984).

159 Pauly, D., Sambilay Jr, V. & Opitz, S. in *Trophic Models of Aquatic Ecosystems. V. Christensen and D. Pauly (eds.) ICLARM Conference Proceedings.* 236-225.

160 Castro, B. & Guerra, A. The diet of Sepia officinalis (Linnaeus 1758) and Sepia elegans (D'Orbigny 1835)(Cephalopoda, Sepioidea) from the Ria de Vigo (Northwestern Spain). *Scientia Marina (Spain)* (1990).

161 Ambrose, R. F. & Nelson, B. V. Predation by Octopus vulgaris in the Mediterranean. *Marine Ecology* **4**, 251-261 (1983).

162 Ajana, R., Techetach, M. & Saoud, Y. Diet of Octopus vulgaris from the moroccan Mediterranean Coast. *Thalassas: An International Journal of Marine Sciences* **34**, 415-420 (2018).

163 Brey, T. A collection of empirical relations for use in ecological modelling. *Naga the ICLARM quarterly* **22**, 24-28 (1999).

164 Brey, T. *Population dynamics in benthic invertebrates. In: A Virtual Handbook Version 01.2. Alfred Wegener Institute for Polar and Marine Research, Germany,* [*http://www.awi-bremerhaven.de/Benthic/Ecosystem/FoodWeb/Handbook/main.html.*](http://www.awi-bremerhaven.de/Benthic/Ecosystem/FoodWeb/Handbook/main.html.), 2001).

165 Karani, I., Kitsos, M.-S., Chartosia, N. & Koukouras, A. Diet composition of the penaeid shrimp, Melicertus kerathurus (Forskål, 1775)(Decapoda, Penaeidae) in the Aegean Sea. *Crustaceana*, 385-396 (2005).

166 Micu, D. & Niţă, V. First record of the Asian prawn Palaemon macrodactylus Rathbun, 1902 (Caridea: Palaemonoidea: Palaemonidae) from the Black Sea. *Aquatic Invasions* **4**, 597-604 (2009).

167 Cristo, M. & Cartes, J. E. A comparative study of the feeding ecology of Nephrops norvegicus (L.),(Decapoda: Nephropidae) in the bathyal Mediterranean and the adjacent Atlantic. *Scientia Marina* **62**, 81-90 (1998).

168 Johnson, M. P., Lordan, C. & Power, A. M. Habitat and ecology of Nephrops norvegicus. *Advances in marine biology* **64**, 27-63 (2013).

169 Acar, S., Ertürk Gürkan, S., Ateş, A. S. & Yalçın Özdilek, Ş. A Contribution on the Diet of the Warty Crab, Eriphia verrucosa (Forskål, 1775) by Informative Bayesian Stable Isotope Mixing Models. *Russian Journal of Marine Biology* **48**, 495-503 (2022).

170 Barker, P. & Gibson, R. Observations on the feeding mechanism, structure of the gut, and digestive physiology of the European lobster Homarus gammarus (L.)(Decapoda: Nephropidae). *Journal of Experimental Marine Biology and Ecology* **26**, 297-324 (1977).

171 Goncalves, R., Lund, I., Sousa, D. & Skov, P. V. Shrimp waste meal (Pandalus borealis) as an alternative ingredient in diets for juvenile European lobster (Homarus gammarus, L.). *Animal Feed Science and Technology* **294**, 115478 (2022).

172 Seyhan, K., Mazlum, E. R., Emiral, H., Engin, S. & Demirhan, S. Diel feeding periodicity, gastric emptying, and estimated daily food consumption of whelk (Rapana venosa) in the south eastern Black Sea (Turkey) marine ecosystem. (2003).

173 Saglam, H. & Duzgunes, E. Biological parameters and feeding behaviour of invasive whelk Rapana venosa Valenciennes, 1846 in the south-eastern Black Sea of Turkey. *Journal of Coastal Life Medicine* **2**, 442-446 (2014).

174 Stadnichenko, S. & Kurakin, A. Feeding intensity and daily mussel consumption of Rapa whelk () in the north-western Black Sea. *Oceanological and Hydrobiological Studies* **51**, 344-354 (2022).

175 Erik, G. & Dağtekin, M. Effect of fishery closure on discard composition of Rapa Whelk, Rapana venosa (Valenciennes 1846) beam trawl fisheries in Black Sea. *Turkish Journal of Fisheries and Aquatic Sciences* **22** (2021).

176 Stoykov, S. & Uzunova, S. Dynamics of macrozoobenthos in the Southern Bulgarian Black Sea coastal and open-sea areas. *Mediterranean Marine Science* **2**, 27-36 (2001).

177 Dumitrache, C. & Abaza, V. The present state of benthic communities in the Romanian coastal waters. *Recherches Marines, INCDM, Constanta* **35**, 61-75 (2004).

178 Todorova, V., Dimitrov, L., Doncheva, V., Trifonova, E. & Prodanov, B. in *Proceedings of the Twelfth International Conference on the Mediterranean Coastal Environment MEDCOAST.* 06-10.

179 Revkov, N. K., Boltacheva, N. A., Timofeev, V. A., Bondarev, I. P. & Bondarenko, L. V. Macrozoobenthos of the Zernov's Phyllophora Field, Northwestern Black Sea: species richness, quantitative representation and long-term variations. *Nature Conservation Research. Заповедная наука* **3**, 32-43 (2018).

180 Sergeeva, N. G. & Gulin, M. B. Meiobenthos from an active methane seepage area in the NW Black Sea. *Marine Ecology* **28**, 152-159 (2007).

181 Teaca, A., Muresan, M., Begun, T., Popa, A. & Ion, G. Marine benthic habitats within a physical disturbed site from the Romanian Coast of the Black Sea. *Journal of Environmental Protection and Ecology* **20**, 723-732 (2019).

182 Kolyuchkina, G. A. *et al.* Benthic community structure near the margin of the oxic zone: A case study on the Black Sea. *Journal of Marine Systems* **227** (2022).

183 Uzunova, S. Benthic communities in the coastal area of the Northern part of the Bulgarian Black Sea region. *Proceedings of the Union of Scientists–Varna*, 65-71 (2012).

184 Uzunova, S., Mihneva, V., Tserkova, F. & Petrova-Pavlova, E. Macrobenthos diversity in the bycatch of rapa welk trawling along the western Black shelf. *International Scientific Advisory Committee*, 49 (2021).

185 Stadnichenko, S. & Zolotarev, V. Estimating the Productivity of the Black Sea Bivalve Chamelea gallinafrom the Size and Biomass of Its Populations. *Russian Journal of Marine Biology* **27**, 130-134 (2001).

186 Revkov, N. & Revkova, T. Long-term Variations in the Black Sea Population of Smooth Scallop, Flexopecten glaber (Linnaeus, 1758)(Bivalvia: Pectinidae): A Review. *Turkish Journal of Fisheries and Aquatic Sciences* **23** (2023).

187 Dalgıç, G. & Ceylan, Y. Seasonal discards and by-catch of striped venus clam (Chamelea gallina)(Mollusca, Bivalves) fishery in the Black Sea. *Turkish Journal of Fisheries and Aquatic Sciences* **12**, 811-816 (2012).

188 Salihoglu, B., Arkin, S. S., Akoglu, E. & Fach, B. A. Evolution of future Black Sea fish stocks under changing environmental and climatic conditions. *Frontiers in Marine Science* **4**, 339 (2017).

189 Palomares, M. & Pauly, D. in *Jellyfish Blooms: Causes, Consequences, and Recent Advances* 11-21 (Springer, 2009).

190 Miladinova, S., Stips, A., Macias Moy, D. & Garcia-Gorriz, E. Revised Black Sea ecosystem model. (2017).

191 Miladinova, S., Stips, A., Garcia-Gorriz, E. & Moy, D. M. in *EUR 28060 EN* (Publications Office of the European Union, 2016).

192 Berov, D., Klayn, S., Deyanova, D. & Karamfilov, V. Current distribution of Zostera seagrass meadows along the Bulgarian Black Sea coast (SW Black Sea, Bulgaria)(2010-2020). *Biodiversity Data Journal* **10** (2022).

193 Aysel, V., Erdugan, H., Duraltarakçi, B. & Okudan, E. Marine algae and seagrasses of Giresun shores (Black Sea, Turkey). *Journal of Black Sea/Mediterranean environment* **11**, 241-300 (2005).

194 Milchakova, N. A. On the status of seagrass communities in the Black Sea. *Aquatic Botany* **65**, 21-31 (1999).

195 Milchakova, N. A. & Phillips, R. C. Black sea seagrasses. *Marine pollution bulletin* **46**, 695-699 (2003).

196 Aysel, V., Erdugan, H., Dural, B. & SükranOkudan, E. Marine algae and seagrasses of Tekirdag (Black Sea, Turkey). *Journal of Black Sea/Mediterranean environment* **12**, 251-267 (2006).

197 Marin, O., Abaza, V., Filimon, A. & Dumitrache, C. Current status of the benthic communities in the Romanian Black Sea waters. *Revista Cercetări Marine-Revue Recherches Marines-Marine Research Journal* **48**, 135-144 (2018).

198 Leonchyk and Bitetto, 2021. Stock Assessment Form Rapa Whelk. https://gfcmsitestorage.blob.core.windows.net/documents/SAC/SAFs/DemersalSpecies/2020/RPW_GSA_29_2020_BGR_GEO_ROU_RUS_TUR_UKR.pdf

XX-XX-XX-XXX-XX-C
