# Supplementary material for: State of the art modelling for the Black Sea ecosystem to support European policies
Source: PLoS One. 2025 Jan 15;20(1):e0312170. doi: 10.1371/journal.pone.0312170 (PMC11734964; doi:10.1371/journal.pone.0312170)
Supplement: Attachment — Submitted filename: PONE-D-24-26903_reviewer.pdf [file pone.0312170.s003.pdf]

PLOS ONE

~~The~~ state of the art modelling for the Black Sea ecosystem to support European policies

--Manuscript Draft--

**Manuscript Number:**	PONE-D-24-26903
**Article Type:**	Research Article
**Full Title:**	The state of the art modelling for the Black Sea ecosystem to support European policies
**Short Title:**	Modelling the Black Sea ecosystem
**Corresponding Author:**	Natalia Serpetti, Ph.D.<br>JRC: European Commission Joint Research Centre<br>Ispra, Varese ITALY
**Keywords:**	Coupled ecosystem models, food web dynamics, hydrodynamic-biogeochemical drivers, ecosystem approach to fisheries, Marine Strategy Framework Directive, Ecopath with Ecosim, Ecospace
**Abstract:**	The Black Sea is affected by numerous anthropogenic pressures, such as eutrophication and pollution through coastal and river discharges, fisheries overexploitation, species invasions, and the impacts of climate change. Growing concerns regarding the cumulative effects of these pressures have necessitated the need for an ecosystem approach to assessing the state of this basin. In recent years, the European Commission-JRC has developed a scientific and modelling tool, the Blue2 Modelling Framework with the aim of exploring the consequences of EU management and policy options on marine ecosystems. This framework has been designed to provide information on specific ecological indicators set out in EU legislation. Here, we present the Blue2 framework for the Black Sea ecosystem. The model represented the mid-1990s' conditions in the Black Sea and included the representation of the ecosystem from primary producers to marine mammals and sea birds. The model simulations covered a period between from 1995-2021. The results showed that gulls & cormorant seabirds, sprat, horse mackerel and mugilidae had structuring role in the food web. Fishing fleet had indirect negative impacts on marine mammals in addition to commercially exploited species. Analysis of the ecosystem indicators confirmed the overall temporal degradation of the Black Sea when comparing results with other Black Sea models, whilst the comparison with the Mediterranean Sea allowed us to identify comparable indicators between similar model structures. The spatial/temporal model successfully simulated the overall ongoing declining dynamics of the Black Sea ecosystem as the biomasses of the majority of the functional groups had significant decreasing trends during the simulation period. This model is the first attempt to represent the historical and current state of the Black Sea ecosystem spatially and temporally, serving as a reference baseline for evaluating policy scenarios and assisting policy makers in the evaluation of potential environmental impacts of management options.
**Order of Authors:**	Natalia Serpetti, Ph.D.
	Chiara Piroddi, Ph.D.
	Ekin Akoglu, Ph.D.
	Elisa Garcia-Gorriz, Ph.D.
	Svetla Miladinova, Ph.D.
	Diego Macias-Moy, Ph.D.
**Additional Information:**	
**Question**	**Response**
**Financial Disclosure**	The author(s) received no specific funding for this work.

Powered by Editorial Manager® and ProduXion Manager® from Aries Systems Corporation

Enter a financial disclosure statement that describes the sources of funding for the work included in this submission. Review the submission guidelines for detailed requirements. View published research articles from *PLOS ONE* for specific examples.<br><br>This statement is required for submission and **will appear in the published article** if the submission is accepted. Please make sure it is accurate.<br><br>**Funded studies**<br>Enter a statement with the following details:<br>• Initials of the authors who received each award<br>• Grant numbers awarded to each author<br>• The full name of each funder<br>• URL of each funder website<br>• Did the sponsors or funders play any role in the study design, data collection and analysis, decision to publish, or preparation of the manuscript?<br><br>Did you receive funding for this work?	
**Competing Interests**<br><br>Use the instructions below to enter a competing interest statement for this submission. On behalf of all authors, disclose any competing interests that could be perceived to bias this work—acknowledging all financial support and any other relevant financial or non-financial competing interests.<br><br>This statement is required for submission and **will appear in the published article** if the submission is accepted. Please make sure it is accurate and that any funding sources listed in your Funding Information later in the submission form are also declared in your Financial Disclosure statement.<br><br>View published research articles from	The authors have declared that no competing interests exist.

PLOS ONE for specific examples.<br><br>**NO authors have competing interests**<br><br>Enter: *The authors have declared that no competing interests exist.*<br><br>**Authors with competing interests**<br><br>Enter competing interest details beginning with this statement:<br><br>*I have read the journal's policy and the authors of this manuscript have the following competing interests: [insert competing interests here]*<br><br>* typeset	
**Ethics Statement**<br><br>Enter an ethics statement for this submission. This statement is required if the study involved:<br><br>• Human participants<br>• Human specimens or tissue<br>• Vertebrate animals or cephalopods<br>• Vertebrate embryos or tissues<br>• Field research<br><br>Write "N/A" if the submission does not require an ethics statement.<br><br>General guidance is provided below. Consult the submission guidelines for detailed instructions. **Make sure that all information entered here is included in the Methods section of the manuscript.**	None of the above applied

**Format for specific study types**<br><br>**Human Subject Research (involving human participants and/or tissue)**<br>• Give the name of the institutional review board or ethics committee that approved the study<br>• Include the approval number and/or a statement indicating approval of this research<br>• Indicate the form of consent obtained (written/oral) or the reason that consent was not obtained (e.g. the data were analyzed anonymously)<br><br>**Animal Research (involving vertebrate animals, embryos or tissues)**<br>• Provide the name of the Institutional Animal Care and Use Committee (IACUC) or other relevant ethics board that reviewed the study protocol, and indicate whether they approved this research or granted a formal waiver of ethical approval<br>• Include an approval number if one was obtained<br>• If the study involved *non-human primates*, add *additional details* about animal welfare and steps taken to ameliorate suffering<br>• If anesthesia, euthanasia, or any kind of animal sacrifice is part of the study, include briefly which substances and/or methods were applied<br><br>**Field Research**<br><br>Include the following details if this study involves the collection of plant, animal, or other materials from a natural setting:<br>• Field permit number<br>• Name of the institution or relevant body that granted permission	
**Data Availability**<br><br>Authors are required to make all data underlying the findings described fully available, without restriction, and from the time of publication. PLOS allows rare exceptions to address legal and ethical concerns. See the PLOS Data Policy and FAQ for detailed information.	Yes - all data are fully available without restriction

A Data Availability Statement describing where the data can be found is required at submission. Your answers to this question constitute the Data Availability Statement and **will be published in the article**, if accepted.<br><br>**Important:** Stating 'data available on request from the author' is not sufficient. If your data are only available upon request, select 'No' for the first question and explain your exceptional situation in the text box.<br><br>Do the authors confirm that all data underlying the findings described in their manuscript are fully available without restriction?	
**Describe where the data may be found in full sentences. If you are copying our sample text, replace any instances of XXX with the appropriate details.**<br><br>• If the data are **held or will be held in a public repository**, include URLs, accession numbers or DOIs. If this information will only be available after acceptance, indicate this by ticking the box below. For example: *All XXX files are available from the XXX database (accession number(s) XXX, XXX.).*<br>• If the data are all contained **within the manuscript and/or Supporting Information files**, enter the following: *All relevant data are within the manuscript and its Supporting Information files.*<br>• If neither of these applies but you are able to provide **details of access elsewhere**, with or without limitations, please do so. For example:<br><br>*Data cannot be shared publicly because of [XXX]. Data are available from the XXX Institutional Data Access / Ethics Committee (contact via XXX) for researchers who meet the criteria for access to confidential data.*<br><br>*The data underlying the results presented in the study are available from (include the name of the third party*	All relevant data are within the manuscript and its Supporting Information files.

and contact information or URL).<br>• This text is appropriate if the data are owned by a third party and authors do not have permission to share the data.<br><br>* typeset	
Additional data availability information:	Tick here if the URLs/accession numbers/DOIs will be available only after acceptance of the manuscript for publication so that we can ensure their inclusion before publication.

Powered by Editorial Manager® and ProduXion Manager® from Aries Systems Corporation

Manuscript

Click here to access/download;Manuscript;BS_state_of_the_art_paper_sub

~~The~~ state of the art modelling for the Black Sea ecosystem to support European policies

Serpetti, N.[1], Piroddi, C. [1], Akoglu[2,3], E., Garcia-Gorriz., E.[1], Miladinova, S. [1], Macias, D. [1]

1 European Commission, Joint Research Centre, Directorate D-Sustainable Resources, Ispra, Italy

2 Middle East Technical University, Institute of Marine Sciences, Department of Marine Biology and Fisheries, P.O. Box 28, Erdemli-Mersin, 33731, Türkiye

3 Middle East Technical University, Climate Change and Sustainable Development Application and Research Center, Ankara, 06800, Türkiye

Abstract

The Black Sea is affected by numerous anthropogenic pressures, such as eutrophication and pollution through coastal and river discharges, fisheries overexploitation, species invasions, and the impacts of climate change. Growing concerns regarding the cumulative effects of these pressures have necessitated the need for an ecosystem approach to assessing the state of this basin. In recent years, the European Commission-JRC has developed a scientific ~~and~~ modelling tool, the Blue2 Modelling Framework with the aim of exploring the consequences of EU management and policy options on marine ecosystems. This framework has been designed to provide information on specific ecological indicators set out in EU legislation. Here, we present the Blue2 framework for the Black Sea ecosystem. The model represented the mid-1990s' conditions in the Black Sea ~~and included the representation of the~~ ecosystem from primary producers to marine mammals and sea birds. The model simulations covered a period ~~between~~ from 1995-2021. The results showed that gulls & cormorant seabirds, sprat, horse mackerel and mugilidae had structuring role in the food web. Fishing fleet had indirect negative impacts on marine mammals in addition to commercially exploited species. Analysis of the ecosystem indicators confirmed the overall temporal degradation of the Black Sea when comparing results with other Black Sea models, whilst the comparison with the Mediterranean Sea allowed us to identify comparable indicators between similar model structures. The spatial/temporal model successfully simulated the overall ongoing declining dynamics of the Black Sea ecosystem as the biomasses of the majority of the functional groups had significant decreasing trends during the simulation period. This model is the first attempt to represent the historical and current state of the Black Sea ecosystem spatially and

temporally, serving as a reference baseline for evaluating policy scenarios and assisting policy makers in the evaluation of potential environmental impacts of management options.

Keywords: coupled ecosystem models, food web dynamics, hydrodynamic-biogeochemical drivers, ecosystem approach to fisheries, Marine Strategy Framework Directive, Ecopath with Ecosim, Ecospace

1. Introduction

Since the 1960s, the Black Sea has undergone a series of transformations driven by numerous anthropogenic stressors. These stressors, acting either concurrently or sequentially, include overfishing [1], pollution [2,3], eutrophication and hypoxia [4], coastal abrasion and erosion [5,6], invasion of alien species [7], and climate change.

The food web dynamics in the Black Sea have undergone significant changes over decades. In the 1960s, the system was predominantly controlled by top-down predator regulation, with species such as Atlantic mackerel, bluefish, and Atlantic bonito being dominant. However, by the 1970s, there was a notable shift towards bottom-up resource control [8], largely attributed to concurrent eutrophication and the overexploitation of apex predators [9]. Recent reports indicate that over 60% of fish stocks are subjected to overfishing in the Mediterranean and the Black Seas [10].

The anthropogenic impacts have significantly diminished both benthic and pelagic biodiversity, while undermining ecosystem resilience. By the late 1980s, the ecosystem, once dominated by small pelagic fish such as anchovy, underwent another transformation marked by the collapse of the anchovy stock and the proliferation of the alien comb jelly species, *Mnemiopsis leidyi* [11,12]. Within a decade from the early 1980s, fisheries yield plummeted by 70% [13].

In the 1990s, active measures were taken to address overfishing and eutrophication [14]; however, despite these interventions, the overall health of the Black Sea ecosystem continues to deteriorate [9]. Climate-induced forces threaten this weak equilibrium [15]. The Black Sea is considered a hotspot of biodiversity [16], yet it is one of the most impacted large marine ecosystems due to anthropogenic pressures [9] and one of the least spatially-assessed marine regions in relation to its biodiversity [17].

Temporal-spatial analysis of species distributions and biodiversity is lacking for the Black Sea, despite the necessity to implement/enforce management policies. Cooperation and

commitment from all countries bordering the Black Sea is required to ensure the long-term health and resilience of this ecosystem. To date, the ecosystem-based management (EBM) approach in the Black Sea has been limited to major commercial fish stocks for fisheries management advice [18].

Policymakers often embrace ecological modelling approaches to assess the impacts of decision-making processes in marine ecosystems [19]. In an evidence-based policy context, policymakers urge spatially and temporally explicit marine ecosystem assessments to develop comparable indicators across regions impacted by multiple stressors cumulatively. In this context, the European Commission and its science Directorate-General (DG), the Joint Research Centre, has developed the Blue2 modelling framework (Blue2MF), which consists of a comprehensive suite of modelling tools designed to simulate EU marine ecosystems, covering all essential components, across various management and policy scenarios [20]. The Blue2MF is extensively used to support EU policies, mainly the Marine Strategy Framework Directive (MSFD) [21–23] and the Zero Pollution Action Plan [24], by analysing the probable future environmental conditions in EU marine regions under different policy scenarios.

The Ecopath with Ecosim food-web modelling [25], which is part of the Blue2MF, has been considered one of the most widely adopted High Trophic Level (HTL) approach to assess cumulative effects, in time and space, of multiple environmental conditions accounting for food-web interactions, anthropogenic stressors such as, fisheries, aquaculture, renewable energy and infrastructures [26,27] and climate/environmental changes [28].

In this study, we present the Blue2MF for the Black Sea ecosystem and provide an initial spatio-temporal assessment of the Black Sea ecosystem since 1995 to delineate the recent conditions of the food web and fish stocks in the Black Sea. The model was set up with a structure to encompass all representative species of the ecosystem [9,18], while also following the rationale used by Piroddi et al. [19] for the Blue2MF of the Mediterranean Sea ecosystem, i.e., incorporating functional groups and/or species that represent the overall biodiversity of the ecosystem, with the same temporal range (1995-2021) and spatial resolution. Having similar ecological, temporal and spatial structure, enable comparison among models, in this case the Black and Mediterranean seas, and indicators, which, occasionally, are sensitive/dependent of model structure [29,30]. The following rationale is important to provide independent and evidence-based support in the whole policies cycle assessment.

2. Materials and Methods

The Blu2MF Black Sea model developed here is a comprehensive suite of modelling tools focusing on different ecosystem components, including atmospheric forcing, freshwater hydrology, marine oceanography, biochemical components, and the marine food web of the ecosystem [20].

BlueMF integrates models for freshwater quantity, LISFLOOD [31], and quality, GREEN [32]) to replicate the conditions of EU rivers, along with atmospheric forcing derived from either reanalysis sources (such as the ERA-5 dataset) or circulation models (such as EuroCORDEX), which account for the atmospheric deposition of critical chemical elements for marine ecosystems, e.g., nitrate from the EMEP model [33]. The core of Blue2MF is an ocean model that consists of a hydrodynamic and a biogeochemical module (also called the lower trophic level (LTL)). The hydrodynamic component is founded on the General Estuarine Transport Model (GETM) [34] and the General Ocean Turbulence Model, whereas the biogeochemical module has been expertly customized for each EU marine region [35]. At the end of this framework, there is the higher trophic level (HTL) food web model (Ecopath with Ecosim (EwE) [25], which is coupled (offline) to the hydrodynamic biogeochemical module of the Blue2MF (Appendix Fig. S1).

In the Mediterranean Sea, the LTL of Blue2MF has been widely utilized to simulate biogeochemical conditions during hindcast periods [36,37] and forecast future conditions [38,39]. The HTL component has been utilized on multiple occasions to investigate the HTL conditions in this basin [19,21,40].

Following this rationale, the marine Black Sea model of Blue2MF consisted of an HTL food web model coupled with its Black Sea LTL hydrodynamic biogeochemical model (GETM-BSEM [41]. In particular, biomass and growth rate data for small and large phytoplankton and zooplankton were used as input data for Ecopath (the static mass-balance module, representing the year 1995), Ecosim (the temporal dynamic module representing the period 1995-2021)) and Ecospace (the spatial dynamic module representing the period 1995-2021).

2.1. Ecopath

The model was built in Ecopath with Ecosim (EwE) version 6.7. Ecopath is a mass balance model across functional groups (FGs) that represents a snapshot of the ecosystem in a given

year. FGs are represented by species or groups of species that share similar predator-prey interactions, temporal biomass trends, ecosystem functioning, and habitat preferences.

FGs were selected based on the composition of the Black Sea commercial fishery [15] and ecosystem dynamics, as observed by Akoglu [9]. In addition, to assess and compare ecosystem structures across MSFD regions, the model was constructed following the same rationale described by Piroddi et al. [19], aiming to represent the diversity of the overall ecosystem and support best policy decisions.

Thus, in contrast to previously built Black Sea models [9,18] top-predator mammal species (bottlenose dolphin, common dolphin and harbour porpoise), seabird species (seagulls & cormorants, terns and pelicans), benthic invertebrates ('mobile' and 'sessile' benthos groups) as well as seagrass and seaweed primary producers were included as FGs.

Input data (biomass, production/biomass, consumption/biomass, diet, and catch/discards) were extracted and/or calculated from in situ literature, stock assessments, or, when lacking, inferred from the Mediterranean model [19].

Catches (1995-2021) from the General Fisheries Commission for the Mediterranean (GFCM) (https://www.fao.org/gfcm/data/capture-production/ar/) were extracted to obtain a list of species that mainly represented the commercial species in the ecosystem. The species were subsequently organized within the defined functional groups (FGs, Table 1). Catches data were separated into landings and discards, by functional groups across the fishing fleets (Beach-Seiner, Trawler, Dredge, Gillnet and Trammel net, Longline, Traps and pods, Purse Seine and Midwater Trawl) following the catch and discard proportions used by Piroddi et al. [19] for the Eastern Mediterranean region, and when available, specific local fishery data by fleets were included (details in the Appendix Table S1).

The biomasses of Atlantic bonito and bluefish were extracted from the literature and scaled down to consider the migratory behaviours of these stocks as they move into the Marmara and the Mediterranean Seas for wintering [42]. This approach was also applied to the Atlantic mackerel group. To account for these migratory behaviours, we also applied positive biomass accumulations for those species, whilst we set a negative biomass accumulations for rapa whelk (*Rapana venosa)* and picked dogfish to better represent the temporal decline observed by their stock assessments (Table S1).

The biomasses for all the other fished groups in 1995 was inferred using catch per unit of effort data (catch from the GFCM and reconstructed effort (for details, see section below)). Catches per Unit of Effort (CPUE) measured only the component of the population that is vulnerable to the gear (gear selectivity, size and age of fish, horizontal and vertical distribution of fish); to account for the total population, we corrected it applying a coefficient factor estimated by the model. For benthic invertebrates, data from literature were used (Table S1).

Ecopath is based on the assumption that the production of one FG is equal to the sum of all predation, non-predatory losses, exports, biomass accumulations, and catches, as expressed by the following equation:

$$P/B_i \cdot B_i = P/B_i \cdot B_i \cdot (1 - EE_i) + \sum_j (Q/B)_{ji} \cdot B_i \cdot DC_{ji} + Y_i + NM_i + BA_i \qquad (1)$$

where B_i is the initial biomass ($t*km^{-2}$), $(P/B)_i$ is the production rate per unit biomass of group *i* ($year^{-1}$), $(Q/B)_i$ is the consumption rate per unit biomass of group *i* ($year^{-1}$), DC_{ji} is the relative proportion of prey *i* in the diet of predator *j*, NM_i is the net migration of prey *i*, BA_i is the biomass accumulation of prey *i*, Y_i is the yield (landings + discards, $t*km^{-2}year^{-1}$) of prey *i*, and EE_i is the ecotrophic efficiency of group *i*, that is, the proportion of production used in the system.

In an Ecopath model, the energy input and output of all the FGs must be balanced under certain ecological and thermodynamic rules. To balance the Black Sea model, we applied a manual mass-balance procedure following Mackinson and Daskalov [43], which modifies the appropriate input parameters employing seven criteria. We balanced the model following the best practice guidelines [30,44,45]: mass balance was obtained primarily by adjusting the diet matrix and the coefficient factor used to estimate biomasses from the CPUE which are the data source with higher uncertainty. Here we presented selected factors, among all parameters, defining the ecological and thermodynamic rules for balancing Ecopath models.

2.1.1. Ecosystem indicators

The trophic flows, encompassing total production, consumption, respiration, catches, and flow to detritus, were estimated to represent the ecosystem structure and exploitation status [46–48]. The trophic level (TL) of each functional group was calculated as

$$TL_j = 1 + \sum_{i=1}^{n} DC_{ij} * TL_i \quad (6)$$

where 'j' is the predator of prey 'i', 'DC_{ji}' is the fraction of prey 'i' in the diet of predator 'j', and TL_i is the TL of prey i. By definition, TL I was attributed to primary producers and detritus, TL II to herbivores, TL III to first-order carnivores, and TL IV to second-order carnivores. This enabled us to calculatethe TL of the catches (TL_C), which is an indicator of "fishing down the food web" effect in the ecosystems [49] as overfishing usually leads to a decrease in the overall trophic level of the catch. TL_C was calculated as

$$TL_{Ci} = \frac{\sum_{i=1}^{n} TL_i * Y_i}{\sum_{i=1}^{n} Y_i} \quad (7)$$

where 'Y_i' refers to the landings of species (group) 'i'.

The total system throughput (TST) was calculated as the sum of all flows in the food web and is an indication of the whole ecosystem size. The total primary production/total system respiration (TPP/ TR) is a metric of system maturity and was calculated to assess the state of the ecosystem *sensu* [46]. The mean transfer efficiency (TE) is the efficiency at which energy is transferred between TLs and an indicator of the functioning of the food web, i.e., how efficiently the energy produced by lower trophic levels are transferred to higher trophic levels. The mean TE was calculated as the geometric mean of the TE for each integer TLs II to IV.

The system omnivory index (SOI) is based on the average omnivory index (OI), which is calculated as the variance of the trophic levels of a consumer's prey groups, indicating predatory specialization [48]. A high SOI is desired in mature ecosystems.

Relative system ascendancy and overhead were also calculated. Ascendancy is a measurement of system growth and the development of network links [50]. Overhead (O) is the energy in the reserve of an ecosystem that reflects the system's strength when it experiences unexpected perturbations [47].

Mixed trophic impact (MTI), which quantifies the impacts of a theoretical change in a specific group's biomass (including fishing activities) on other groups in the ecosystem [51], assessing both direct (e.g., predation) and indirect (e.g., competition) trophic relationships, was also calculated as

$$MTI_{ij} = DC_{ij} - FC_{ij} \quad (8)$$

where 'DC_{ij}' is the diet composition term expressing how much 'j' contributes to the diet of 'i', and 'FC_{ji}' is the proportion of predation on 'j' that is due to 'i' as a predator. MTI scales between -1, indicating a strong negative impact, and 1, indicating a strong positive impact, between FGs in the food web.

Finally, the keystoneness index (KS, Libralato et al. [52], which shows the FGs with a disproportiantely influential role in the food web contrary to their biomasses, was calculated as

$$KS_i = \log(\varepsilon_i * \frac{1}{p_i}) \qquad (9)$$

where 'ε_i' is the overall effect expressed as the square root of the sum of 'm_{ij}' square (with 'm_{ij}' being the relative impact of a slight increase in biomass of impacting group 'i' on biomass of impacted group 'j'), and p_i is the contribution of the functional group to the total biomass of the food web.

2.2. Ecosim

Ecosim uses Ecopath's outputs as starting information to simulate the dynamics of each FG over time. In Ecosim, a series of differential equations describe the changes in biomass for each FG (i) over time, as follows:

$$\frac{dB_i}{dt} = \left(\frac{P}{Q}\right)_i \cdot \sum Q_{ji} - \sum Q_{ji} + I_i - (M_i + F_i - e_i) \cdot B_i \qquad (10)$$

Where dB_i/dt is the biomass change of group i in the time step dt, $(P/Q)_i$ is its production/consumption ratio, i.e., gross growth efficiency, Q_{ji} is the consumption of group j (predator) on prey group(s) i, Q_{ij} is the consumption for predation by all predators j on group i (prey), I_i is the immigration rate, B_i is the biomass at time t, M_i and F_i are the natural and fishing mortality rates of group i, respectively, and e_i is the density-dependent emigration rate. Fishing mortality or fishing effort is used to drive the model.

Ecosim predicts biomasses and catches and compares them to observed time-series data of biomasses and catches using a log-likelihood sum of squares for calibration [53]. For each predator, consumption (Q_{ij}) is calculated based on the "foraging arena" theory, which divides the biomass of the prey into a vulnerable and a non-vulnerable fraction and the transfer rate or vulnerability between the two fractions determines the trophic flow between the predator and the prey [54]. The vulnerability concept incorporates density-dependent processes and

expresses how far a group is from its carrying capacity. Default values of vulnerability (v = 2) represent a mixed trophic flow, a low value (v < 2) indicates a 'bottom-up', donor-driven, flow control and a situation closer to carrying capacity, while a high value (v > 2) indicates a 'top-down', predator-driven, flow control and a situation further away from carrying capacity. For each predator-prey interaction, the consumption rates are calculated as:

$$Q_{ij} = \frac{a_{ij} \cdot v_{ij} \cdot B_i \cdot B_j \cdot T_i \cdot T_j \cdot M_{ij} / D_j}{v_{ij} + v_{ij} \cdot T_i \cdot M_{ij} + a_{ij} \cdot M_{ij} \cdot B_j \cdot T_j / D_j} \cdot f(Env_{function}, t) \quad (11)$$

where a_{ij} is the rate of effective search for prey *i* by predator *j;* T_i represents prey's relative feeding time; T_j is the predator's relative feeding time; M_{ij} is the mediation forcing effects due to the presence of a third indirectly interacting species with the prey-predator pair in the food web; v_{ij} is the vulnerability parameter, which is akin to the half-saturation constant in Holling-type functional responses [55]; D_j represents the effects of handling time as a limit to consumption rate; and *f(Envfunction,t)* is the environmental response function that restricts the size of the foraging arena (or consumption) to account for external environmental drivers changing over time [28,56]. Species-specific response functions to environmental factors were defined by minimum, 10th and 90th percentiles and maximum values of their optimal niches extracted from AquaMaps (https://www.aquamaps.org/) within the Black Sea domain. For functional groups defined by multiple species, the parameters were calculated as weighted means by species biomass composition (Table S3).

For the Black Sea model, we used relative temporal changes in fishing efforts by fleet and net primary productivity for small and large phytoplankton, derived from the Blue2MF hydrodynamic biogeochemical model (GETM-BSEM [41]) to drive the model and predict temporal biomass and catch changes (1995-2021).

"Fishing effort" (kW*days^{-1}) was calculated as the product of the number of fishing vessels, Kilowatt (kW) per vessel, and the number of days spent fishing. For EU countries, we used data from the official European Commission databases (STECF https://stecf.ec.europa.eu/index_en), while for other countries, we considered the available literature [10,57–61]. To account for improvements in technology (e.g., mobile phones, GPS, sonar, radio) that were not captured by kW as a measure of effort, a conservative technological "creep factor" of approximately 0.8% per year was used, as observed by Damalas et al. [62] and the European Court of Auditors [63].

Stock assessment data were used to validate biomasses and catches for turbot, whiting, red mullet, picked dogfish, horse mackerel, sprat, anchovy, and rapa whelk, while the International Commission for the Conservation of Atlantic Tunas (ICCAT) database (https://www.iccat.int/en/accesingdb.HTML) was used for large pelagic fish. The model was fitted [64] versus time series of observations of relative biomasses for stock-assessed species and relative catches for all fished functional groups (reported by the GFCM database). The fitting procedure is a routine that minimizes the sum of squares (SS) ratio between predicted and observed data as a metric for assessing the model performance. The fishing effort, primary production (PP) of large and small phytoplankton groups over time were used as the main forcing time series to drive the model.

The fitting procedure followed the approach described by Mackinson [53]. This method uses the Akaike Information criterion (AIC) [65,66]:

$$AIC = nlog\ (minSS/n) + 2k \qquad (12)$$

where n is the number of observations, $minSS$ is the minimum sum of squares resulting from the comparison of predicted with observed datasets, and k is the number of parameters to test statistical hypotheses related to changes in predator-prey dynamics, changes in productivity, impact of fishing, and possible combinations of the above-mentioned factors. The AIC is a statistical measure for model selection and penalizes for fitting too many parameters. AIC is used to choose the "best" model (the one yielding the lowest AIC) considering a good fit and the least number of estimated parameters. In this study, we used the second-order Akaike Information Criterion (AIC_c) calculated as follows:

$$AIC_c = AIC + 2k(k-1)/(n-k-1) \qquad (13)$$

to account for small sample sizes (n= observations) in the dataset.

39 independent time series were used during the calibration process using different weights assigned to the time series to reflect the uncertainties of the data. We identified the best-fitted model using "by-predator" setting; additional vulnerabilities were found using "by-predators-preys" setting with a hybrid fitting approach [67,68]. A thousand Monte Carlo runs were performed with the "best" fitted model to assess the uncertainties (5th and 95th percentiles) of the predictions using 10% variability in the input data (B, P/B, Q/B, EE).

Statistical significance of the changes in the temporal trends of simulated biomasses were analysed with time series and trend analyses. First, time series of simulated biomasses for the functional groups with observed data were decomposed into their seasonal, trend and irregular components using moving averages. The time series were then detrended by subtracting the time series of seasonal component from the time series of biomasses using “stats” package in R [69]. Finally, nonlinear trend analysis was done using the “zyp” package in R [70] and the data were pre-whitened prior to trend analysis to eliminate autocorrelation as model simulated values are known to be hampered from autocorrelation. the Zhang’s method [71].

2.3. Ecospace

Ecospace is the spatiotemporal dynamic module of EwE and, as for Ecopath and Ecosim modules, it is coupled with the hydrodynamic biogeochemical model GETM-BSEM outputs, namely depth, annual bottom oxygen concentration, temperature, salinity and monthly primary/secondary productivity (small and large phytoplankton and zooplankton) which drive the spatial distributions.

Since Ecospace does not explicitly account for dynamics in the water column [72], we extracted data on salinity, temperature, primary productivity and planktonic biomass for distinct depth ranges: surface (0–10 m), euphotic zone (0–50 m), entire water column (0–2000 m), and (4) bottom (maximum depth). All species are distributed in space according to their dispersal rates (Table S2) in a two-dimensional spatial grid (~3.7 km x 3.7 km) in relation to their depth-optimal niches (Table S3) from the coastline and to a maximum depth of 2212 m (Fig. 1A). The bottom oxygen concentration was used to drive the benthic species distributions to avoid their distributions from anoxic bottom conditions (Fig. 1B) (< 10 mmol O_2/m^2). Salinity integrated over 50m depth was only applied to the bivalve group, as values below 10‰ have a negative influence on the Mediterranean mussel, *Mytilus galloprovincialis* [73], the dominant species within this functional group. For the model hindcast, temperature and salinity (Fig. 1C and D) were not used, with the above exception. Table S3 shows the response functions to environmental variables; in bold, the functions applied in the hindcast.

Spatiotemporal changes in planktonic groups (small and large phytoplankton and zooplankton) were used in Ecospace as absolute biomass (Fig. 1 E-H), whereas the Net Primary Production of 1995 was used in the Ecospace base maps (Fig. 1 I).

Ecospace simulations were run with 5 years spin-up to allow species to be distributed in space in relation to their environmental niches before applying the temporal changes in planktonic biomasses and fishing effort.

Within the Ecospace module, the Habitat Foraging Capacity (HFC) allows to assess the suitability areas of the modelled species/functional groups based on the cumulative effects of the applied environmental conditions [74]. The HFC maps were extracted and qualitative validated against AquaMaps suitability maps, downloaded and standardised for the Black Sea domain [75] (https://www.aquamaps.org/)

Fig. 1. Black Sea environmental and LTL biological outputs used as inputs and drivers in the HTL model.
(A) Depth (m); (B) Bottom oxygen (averaged over 1995-2021); (C) Temperature averaged over 50m (averaged over 1995-2021); (D) Salinity averaged over 50m (averaged over 1995-2021); (E) Small phytoplankton integrated over 50m (averaged over 1995-2021); (F) Large phytoplankton integrated over 50m (averaged over 1995-2021); (G) Small zooplankton integrated over 50m (averaged over 1995-2021); (H) Large zooplankton integrated over 50m (averaged over 1995-2021); (I) Primary production integrated over 50m.Results and discussion.

2.4. Ecopath

We modelled the entire Black Sea excluding the Azov Sea, covering approximately 423,000 km^2 with a maximum depth of 2,212 m .

The final Blue2MF Black Sea HTL model, representing a snapshot of the ecosystem in 1995, has 47 functional groups (FGs), consisting of three dolphin species, three seabird groups, 24 fish groups, eight invertebrates groups, five planktonic groups, seagrass and seaweed, discards and detritus, and eight fishing fleet types (Fig. 2, flow diagram). The model was balanced following the PREBAL ecological and thermodynamic rules (Appendix Fig. S2). The full list of species/FGs and the basic estimates of the balanced model are given in Table 1, while details on input data calculations and references per FGs are presented in Table S1. Other inputs (landings, discards, effort and diet in Appendix 2).

Fig. 2. Energy flow and biomass diagram of the Black Sea ecosystem (1995). Functional groups (grey) and fleets (red) are represented by nodes. The relative sizes of the nodes for functional groups are proportional to their biomasses in the ecosystem, whereas the relative sizes of the nodes for fishing fleets are proportional to their catch volumes (fishing fleets: Beach-Seiner, Trawler, Dredge, Gillnet and Trammel net, Longline, Traps and pods, Purse Seine, Midwater Trawl). The functional groups are ordered by their trophic levels (y-axis) and their preferred domain (bottom x-axis Pelagic vs Demersal vs Benthic). Predator–prey relationships are expressed with a gradient yellow (predator) to blue (prey) line. Map was created by the authors using MATLAB ~~software~~ vR2014b.

Table 1. Model input parameters of the Black Sea balanced model for 1995. TL, trophic level; B= Biomass (t km^{-2}); P/B= Production/biomass ($year^{-1}$); Q/B= Consumption/biomass ($year^{-1}$); EE= Ecotrophic Efficiency, P/Q= Production/consumption ($year^{-1}$); BA= Biomass accumulation (t km^{-2}).

Num	Group name	TL	B	P/B	Q/B	EE	P/Q	BA
1	Bottlenose dolphin	4.23	0.013	0.07	12.45	0.151	0.01	0
2	Common_dolphin	4.29	0.016	0.04	13.65	0.252	0.00	0
3	Harbour porpoise	4.21	0.012	0.03	16.09	0.554	0.00	0
4	Seagulls & Cormorants	3.38	0.0009	0.34	192.63	0.017	0.00	0
5	Terns	3.36	0.0001	0.30	173.45	0.073	0.00	0
6	Pelicans	3.69	0.0002	0.42	54.32	0	0.01	0
7	Other large pelagic fish	3.80	0.005	0.77	3.71	0.192	0.21	0
8	Atlantic bonito	3.89	0.072	0.59	7.55	0.521	0.08	0.011
9	Bluefish	3.71	0.043	0.87	5.34	0.391	0.16	0.006
10	Mackerels	3.65	0.130	0.78	4.51	0.792	0.17	0.019
11	Medium pelagics	3.77	0.055	0.81	7.21	0.630	0.11	0
12	Horse mackerel	3.44	0.229	0.92	8.35	0.890	0.11	0
13	Anchovy Ad.	3.23	1.694	1.78	9.68	0.563	0.18	0
14	Anchovy Juv.	3.23	0.300	1.47	22.71	0.694	0.06	0
15	Sprat	3.21	0.611	1.08	11.02	0.846	0.10	0
16	European pilchard	3.11	0.067	1.39	9.47	0.828	0.15	0
17	Other small pelagic fish	3.29	0.029	1.14	5.96	0.707	0.19	0
18	Large demersals	3.79	0.011	0.42	2.51	0.501	0.17	0
19	Medium demersal benthopelagic fish	3.21	0.052	0.88	4.48	0.736	0.20	0
20	Whiting	3.53	0.080	1.13	5.15	0.880	0.22	0
21	Mugilidae	2.30	0.098	0.67	5.76	0.875	0.12	0
22	Sparidae	3.30	0.022	0.71	6.35	0.414	0.11	0
23	Turbot	2.97	0.011	0.71	4.66	0.761	0.15	0
24	Flatfish	2.98	0.015	0.61	3.92	0.973	0.15	0
25	Red mullet	3.18	0.053	0.99	6.62	0.777	0.15	0
26	Striped mullet	3.23	0.042	1.12	7.05	0.894	0.16	0
27	Small demersal	2.95	0.110	0.93	9.27	0.749	0.10	0
28	Picked dogfish	3.62	0.009	0.30	2.95	0.396	0.10	-0.001
29	Demersal sharks	3.63	0.020	0.30	2.75	0.731	0.11	0
30	Rays	3.46	0.030	0.20	2.81	0.493	0.07	0
31	Benthic cephalapods	3.31	0.035	2.06	6.88	0.694	0.30	0
32	Shrimp/prawns	2.70	0.107	3.46	11.53	0.845	0.30	0
33	Other decapods	2.85	0.114	3.54	11.79	0.735	0.30	0
34	Rapana venosa	2.44	0.367	0.78	7.98	0.935	0.10	-0.055
35	Gastropoda	2.00	0.541	1.82	15.00	0.494	0.12	0
36	Bivalvia	2.00	1.630	1.25	10.04	0.923	0.12	0
37	Mobile benthos	2.00	0.891	2.86	26.03	0.570	0.11	0
38	Sessile benthos	2.17	0.740	1.95	14.79	0.647	0.13	0
39	Jellyfish	2.91	1.565	3.94	27.92	0.024	0.14	0
40	Zoo large	2.26	6.525	70.59	220.59	0.756	0.32	0
41	Zoo small	2.00	0.715	52.15	155.65	0.521	0.34	0
42	Phy large	1.00	10.838	102.5		0.805		0
43	Phy small	1.00	2.405	156.3		0.682		0
44	Seagrass	1.00	0.696	2.53		0.036		0
45	Seaweed	1.00	1.091	1.53		0.310		0
46	Discards	1.00	0.074			0.894		0
47	Detritus	1.00	22.928			0.208		0

2.4.1. Ecosystem indicators

Functional groups are ranked by their TLs ranging from 1 (primary producers) to 4.29 (marine mammals); the highest TLs were found for ‘Common dolphin’ followed by ‘Bottlenose dolphin’, and ‘harbour porpoise’ (TL ≥ 4) (Table 1). ‘Seagulls & cormorants’ and ‘terns’, despite being considered top predators, showed relatively low TLs (3.38 and 3.36

respectively) due to the presence of discards and zooplankton in their diet. In EwE, discards are assigned to a detritus group with TL = 1, seabirds that feed considerably on discards, lowering the TL of those groups [76]. Several groups belonging to the pelagic and demersal domains had a TL greater than 3, while most of the functional groups with a TL smaller than 2 belonged to the benthic domain (Table 1 and Fig. 2).

Other ecosystem indicators are listed in Table 2. Trophic level of the community (TL>1) was 2.5, which was slightly higher than that in the Mediterranean Sea in 2000 but lower than that in the Black Sea ecosystem in 1960. The TL of the catches in 1995, which indicates the exploitation status of fish populations [49], was much lower (2.97) than that in the Black Sea in the 1960s (3.52) and the 1980s (3.08), highlighting the continuous degradation of this ecosystem [9,15].

The relative proportions of system throughput (TST) were in agreement with previous findings [9,15], and were composed of flows by consumption (38%) and respiration (23%), followed by flow to detritus (22%) and exports (17%). Comparing the absolute values of the total system throughput is not straightforward because this index is sensitive to the model structure [77]. Previous Black Sea models (Table 2) reported a TST of ~10,210 t km^{-2} yr^{-1} for 1995 and ~2007 t km^{-2} yr^{-1} for 1960; however, these models showed simpler structures than the model developed in this study, with only 10 and 22 functional groups, respectively. The TST value calculated in this study (Table 2) concurred with the value for the Mediterranean Sea model [76] that had the same model structure.

The ratio of total primary productivity to total respiration (TPP/TR), which is expected to be close to 1 in developed ecosystems, is an indication of ecosystem maturity [46]. Although Black Sea was in a more mature state than the Mediterranean Sea (Table 2), our results showed that the Black Sea was losing this balance as TPP/TR increased since the 1960s. The transfer efficiency (TE) has also been decreasing over time in the Black Sea ecosystem, indicating inefficient functioning of the food web in terms of energy transfers to higher trophic levels. This could be a reason for the lack of recovery of higher-trophic-level predatory fish species in the ecosystem in addition to their overexploitation by fisheries.

The system omnivory index (SOI) indicates the range of feeding interactions in the food web; therefore, this index is also sensitive to the model structure, which is confirmed by our results showing a similar SOI with the Mediterranean Sea model [76].

System ascendency and overhead show the organizational degree in an ecosystem and its resilience against stress [50,78], and the results indicated higher values for both indices for the Black Sea than for the Mediterranean Sea (Table 2).

Heymans et al. [77] highlighted that a direct comparison of absolute indicator values generated by EwE across different ecosystems should take ecosystem traits such as type, size, depth, and location as well as model's topology into consideration. In this study, we compared these indicators with a Mediterranean model built with the same model structure [19] and with other Black Sea EwE models built with different structures for different periods. The overall analysis of these statistical indices revealed that TST and SOI, as well as indices based on the trophic level greater than 1 (either TL of the community and catches) and transfer efficiency, were comparable with similar model structures, while TPP/TR, ascendency, and overhead were more ecosystem-dependent. However, our comparisons indicated a more deteriorated ecosystem condition in the Black Sea with respect to the results provided by earlier modelling studies.

Table 2. **Summary statistics for the Black Sea and Mediterranean Sea.**

**Indicators**	**Black Sea 1995 (this study)**	**Black Sea 2000 (Akoglu et al, 2014)**	**Black Sea 1960 (Akoglu 2023)**	**Mediterranean Sea 2000 (Piroddi et al., 2015)**
Mean TL_community (TL>1)	2.50	-	2.77	2.18-2.36
Mean TL_catches (TL>1)	2.97	3	3.52	3.08
TST (t km^{-2} yr^{-1})	4008	10210	2007	4000
TPP/TR	1.77	1.16	1.1	5.55
Mean TE (%)	6.01	7.4	13.1	9.2
SOI	0.22	0.12	0.15	0.27
Ascendency (%)	27.50	31.7	26.5	42.9
Overhead (%)	72.50	68.3	73.5	57.1

MTI analysis (Fig. 3) revealed that the majority of predators exerted a direct negative impact on their prey due to diet preferences. Functional groups negatively impacted themselves due to cannibalism or inter-species competition. Planktonic groups had predominantly positive effects on all pelagic and demersal groups in the system (e.g., through a bottom-up effect). However, these planktonic groups had negative impacts on benthic groups, as they were preyed upon by the aforementioned groups (e.g., through a top-down effect).

The results also identified strong direct and indirect negative impacts of gillnets & trammels on dolphins (directly as by-caught by this fleet, and indirectly for targeting the same "prey"

species) and on turbot and demersal sharks. An indirect positive effect was found for prey species of the above groups. Trawlers showed a positive indirect impact on seagulls that feed on discards, and a negative direct impact on the main trawling target demersal species. Longlines had a negative impact on seagulls & cormorants as by-caught by this fleets (Appendix 2), and an indirect positive impact on terns due to their competition with seagulls. Purse-seiners had direct negative impacts on bluefish and anchovy as well as on common dolphins by direct by-catches, but also indirectly, as this fleet targets dolphin's prey.

The species with the highest keystone index value (Fig. 4) in the system was large zooplankton (40); however, by definition, keystone species are the species with a relatively low biomass, but their biomass changes would have a disproportionately large effect on the ecosystem structure [79]. According to this definition, seagulls& cormorants (4), followed by sprat (15), mugilidae (21), mobile benthos (37), horse mackerel (12) and bottlenose dolphin (1) were the functional groups with the highest keystones index values. In the Black Sea model, representing the early 1960s, demersal sharks and dolphins were the keystone species [9]. This contrasting finding indicated that a change of control on the food web occurred since the 1960s, from a top-down to a bottom-up control.

Fig. 3. Mixed trophic impact relationships between functional groups and fisheries. Positive values (from white to blue) indicate positive impacts, and negative values (from white to red) indicate negative impacts. "☑" symbol indicates MTI value greater than 0.2, "!" symbol indicates MTI value between 0.2 and -0.2, "✗" symbol indicates MTI value smaller than -0.2.

Fig. 4. Relative total impact (ε_i) versus keystoneness (KS_i). The size of the circles is proportional to the species/group biomass. For the group numbers, refer to Table 1.

2.5. Ecosim

The fitting routine improved the model performance by 24% compared to the baseline fishing scenario (Table 3). Fig. 5 and Fig. S3 displayed the best fitted model predictions (lines) plotted against observations (points) when available for the 1995-2021 period, showing a

good fit for stock assessed species (Fig. 5) horse mackerel, anchovy, sprat, red mullet, whiting, turbot, picked dogfish and *Rapana venosa*.

Table 3. **Comparison across selected stepwise fitting interactions**.

Model fishing baseline and the two fitting steps, showing the number of total parameters estimated (K, Vulnerabilities (Vs)), number of years of the time-series, number of observations, model sum of squares (SS), SS percentage of contribution to the fitting, and second-order Akaike Information Criterion (AIC_C).

AIC test	Number of years	Number of observations	Number of estimated vulnerabilities (K) (K_{max}=38)	SS	AICc	Contribution to SS fitting
Fishing baseline	27	1029	0	131.4	-2117.7	-
Best fitted "by-predator"	27	1029	33	100.6	-2322.7	23.5%
Hybrid fitting	27	1029	5	99.7	-2328.4	0.9%

Fig. 5. Modelled biomass predictions of the best-fitted model.

Predictions (lines) versus observed (points) estimated by statistical stock assessments for the 1995-2021 period.

The fitted model showed good agreement with most biomass observations for stock-assessed species: all the commercially exploited species except red mullet and turbot declined since 1995 (Fig. 5). According to the trend analysis, all the simulated biomasses except juvenile anchovy had significant decreasing trends (Fig. 5 and Table S4). The only species with a significant increasing trend was red mullet (Table S4). The declines could be attributed to poorly-managed fisheries at national level and the lack of a basin-wide management policy, considering that the Black Sea fish stocks are shared due to the high level of connectivity , and as well as the migratory behaviours of the primarily exploited species [80]. The statistically significant increase simulated in the red mullet biomasses could be related to the simulated decreases in its competing demersal species like Sparidae.

The model, however, was unable to reproduce the recent increase in turbot biomass (2017-2021) predicted by the stock assessment. This increase could be captured by forcing the simulated catches for those years (Fig. S4). This means that the model uses the observed catch

trend for all countries (Ukraine, Romania, Bulgaria, Russia, and Turkey) in the Black Sea instead of predicting it. Overfishing has caused a strong decline in turbot since 1980; for this reason, since 2016, a fishing management plan has been put in place by the European Commission for Romania and Bulgaria in the Black Sea [81]. Turkey, the main ~~fishery~~ country in the Black Sea, did not support these management restrictions. According~~ly~~ to Hulak et al (2021), the decrease in turbot landings reported in the Turkish catch statistics, after 2002, is a consequence of the effort of riparian countries (Ukraine, Romania, and Bulgaria) to stop illegal Turkish turbot fisheries in their EEZ. We presume that abovementioned restrictions/controls could be the consequence of the increase in biomass predicted by the model.

2.6. Ecospace

Ecospace output represented the species distribution of the functional groups driven by prey distributions, and avoidance of predators (including fisheries) within their preferred habitat preference (e.g., depth, oxygen) (Fig. 6 and S5). Unfortunately, the output distributions of the modelled functional groups should be considered with caution, due to the lack of spatial data for many Black Sea species. Yet, qualitative validation can be assessed with available information. For instance, the predicted spatial biomass distributions of marine mammals (Fig. S5, upper panel) in shallower regions for bottlenose dolphins and harbour porpoises and offshore areas for common dolphins are in line with aerial surveys and species distribution models [82,83]. Seabirds showed distributions over waters shallower than 500 m (Fig. S5, lower panel), mainly in the North and West of the basin where their preys are distributed following a similar pattern as observed by Piroddi et al.[21] for the neighbour Mediterranean Sea.

The distributions of commercial migratory pelagic species, such as Atlantic bonito, bluefish and the mackerels groups were only driven by their prey distribution as migration patterns of these species were not considered in this study (Fig. 6 and S6).

Among the stock-assessed species (Fig. 6), sprat and anchovy were the species that mainly drove the distribution of many pelagic predators in the model. The anchovy distribution have a strong seasonal pattern migrating between North-Western shelf to the south of the basin, moreover in winter, the highest anchovy biomass is distributed in the south-east where a significant portion of anchovy catches are from [80,84]. Annual averaged maps here

presented may not represent these spatial seasonal patterns successfully; however, overall, our predicted distributions agreed well with those produced by stock assessment models [80,84].

Benthic species had a confined distribution (Fig. 6 and S6) over the continental shelf, due to the anoxic conditions that occur in deeper waters where the annual bottom oxygen concentration is lower than 10 mmol O_2/m^2 (Fig. 1C) whilst many demersal groups have confined distributions for trophic relationships on benthos. The spatial distribution of picked dogfish (Fig. 6) agreed with previous reported data, however we might underestimate their presence in ~~the~~ shallow waters of the west side of the basin as we do not represent the migration pattern with the Azov Sea [85]. A qualitative validation of the Ecospace habitat capacities of stock assessed demersal species whiting, turbot, red mullet and picked dogfish showed an overall agreement with the maps of habitat suitability model extracted from AquaMaps [75] (Fig. 7). Differences such as lower habitat capacity values towards the centre of the basin could be due to the different model resolutions between Ecospace and AquaMaps and an overestimation of the species sensitivity at lower bottom oxygen concentration.

Overall, our results showed the highest biodiversity in the North-Western shelf, highlighting even further the fragile status of the region and the necessity to prioritize spatial management actions to preserve the remaining biodiversity.

Fig. 6. Biomass spatial distributions of the stock-assessed species.

Data are presented as $t*km^{-2}$ averaged over all modelled years 1995-2021.

Fig. 7. Qualitative spatial validation of stocked assessed species.

Habitat capacity computed by Ecospace model (right panels) versus AquaMaps suitability maps (left panels).

3. Conclusions

Since the 1960s, the Black Sea has undergone a series of transformations driven by numerous anthropogenic stressors. These stressors, acting either concurrently or sequentially, include overfishing [1], pollution [2,3], eutrophication and hypoxia [4], coastal abrasion and erosion [5,6], invasion of alien species [7], and climate change [86]. These multiple anthropogenic pressures and the current conflict happening in Ukraine stress necessitates even more the need of a temporal and spatial assessment tool enable to evaluate the single and cumulative impacts of these pressures.

This new model and its findings constitute the reference baseline (defined as the past and current status) for the Black Sea ecosystem. The reference baseline is an essential component of Blue2MF for assessing the health status of European seas in forward-looking scenarios (2050). These scenarios are part of the Blue2.3 project, which aims to support the potential revision of the MSFD, analysing the impacts of policy options on marine environmental status by modelling scenarios. Such efforts is needed to directly assist spatial management actions (e.g., by prioritizing specific areas of concern) and support management decisions.

Acknowledgments

We thanks Dr Andrea Pierucci from the European Commission, Joint Research Centre in Ispra for the support and guidance on stock assessment data.

References

1. Mee LD. The Black Sea in Crisis: A Need for Concerted International Action. Ambio. 1992;21: 278–286.

2. Topçu EN, Öztürk B. Abundance and composition of solid waste materials on the western part of the Turkish Black Sea seabed. Aquat Ecosyst Health Manag. 2010;13: 301–306. doi:10.1080/14634988.2010.503684

3. Ioakeimidis C, Zeri C, Kaberi H, Galatchi M, Antoniadis K, Streftaris N, et al. A comparative study of marine litter on the seafloor of coastal areas in the Eastern Mediterranean and Black Seas. Mar Pollut Bull. 2014;89: 296–304. doi:10.1016/j.marpolbul.2014.09.044

4. Pokazeev KV, Sovga E, Chaplina T. Pollution in the Black Sea. Switzerland: Springer, Cham; 2021.

5. Kosyan RD, Velikova VN. Coastal zone – Terra (and aqua) incognita – Integrated Coastal Zone Management in the Black Sea. Estuar Coast Shelf Sci. 2016;169: A1–A16. doi:10.1016/j.ecss.2015.11.016

6. Tătui F, Pîrvan M, Popa M, Aydogan B, Ayat B, Görmüș T, et al. The Black Sea coastline erosion: Index-based sensitivity assessment and management-related issues. Ocean Coast Manag. 2019;182: 104949. doi:10.1016/j.ocecoaman.2019.104949

7. Shalovenkov N. Chapter 31—alien species invasion: case study of the Black Sea. Wolanski E, Day JW, Elliott M, Ramachandran R, eds. Coasts and Estuaries. Wolanski E, Day JW, Elliott M, Ramachandran R, eds. Amsterdam: Elsevier; 2019. pp. 547–568.

8. Oguz T, Gilbert D. Abrupt transitions of the top-down controlled Black Sea pelagic ecosystem during 1960–2000: Evidence for regime-shifts under strong fishery exploitation and nutrient enrichment modulated by climate-induced variations. Deep Sea Res Part Oceanogr Res Pap. 2007;54: 220–242. doi:10.1016/j.dsr.2006.09.010

9. Akoglu E. Ecological indicators reveal historical regime shifts in the Black Sea ecosystem. PeerJ. 2023;11: e15649. doi:10.7717/peerj.15649

10. FAO. The State of Mediterranean and Black Sea Fisheries 2022. Rome, Italy: FAO; 2022. doi:10.4060/cc3370en

11. Kideys AE. Fall and Rise of the Black Sea Ecosystem. Science. 2002;297: 1482–1484. doi:10.1126/science.1073002

12. Daskalov G, Grishin A, Rodionov S, Mihneva V. Trophic cascades triggered by overfishing reveal possible mechanisms of ecosystem regime shifts. Proc Natl Acad Sci U S A. 2007;104. doi:10.1073/pnas.0701100104

13. Oguz T, Akoglu E, Salihoglu B. Current state of overfishing and its regional differences in the Black Sea. Ocean Coast Manag. 2012;58: 47–56. doi:10.1016/j.ocecoaman.2011.12.013

14. Dorofeev VL. Modeling of decadal variations in the Black-Sea ecosystem. Phys Oceanogr. 2009;19: 400–409. doi:10.1007/s11110-010-9062-6

15. Akoglu E, Salihoglu B, Libralato S, Oguz T, Solidoro C. An indicator-based evaluation of Black Sea food web dynamics during 1960–2000. J Mar Syst. 2014;134: 113–125. doi:10.1016/j.jmarsys.2014.02.010

16. Almpanidou V, Doxa A, Mazaris AD. Combining a cumulative risk index and species distribution data to identify priority areas for marine biodiversity conservation in the Black Sea. Ocean Coast Manag. 2021;213: 105877. doi:10.1016/j.ocecoaman.2021.105877

17. Vaughan D, Korpinen S, Nygård H, Andersen J, Murray C, Kallenbach E, et al. Biodiversity in Europe's seas. 2020.

18. Salihoglu B, Arkin SS, Akoglu E, Fach BA. Evolution of Future Black Sea Fish Stocks under Changing Environmental and Climatic Conditions. Front Mar Sci. 2017;4. doi:10.3389/fmars.2017.00339

19. Piroddi C, Coll M, Macias D, Steenbeek J, Garcia-Gorriz E, Mannini A, et al. Modelling the Mediterranean Sea ecosystem at high spatial resolution to inform the ecosystem-based management in the region. Sci Rep. 2022;12: 19680. doi:10.1038/s41598-022-18017-x

20. Stips A, Dowell, Mark, Somma, Francesca, Coughlan, Clare, Piroddi, Chiara, Bouraoui, Faycal, et al. Towards an integrated water modelling toolbox. Luxemburg: European Commission; 2015. Report No.: JRC92843. Available: https://mcc.jrc.ec.europa.eu/documents/201603214045.pdf

21. Piroddi C, Akoglu E, Andonegi E, Bentley JW, Celić I, Coll M, et al. Effects of Nutrient Management Scenarios on Marine Food Webs: A Pan-European Assessment in Support of the Marine Strategy Framework Directive. Front Mar Sci. 2021;8: 179. doi:10.3389/fmars.2021.596797

22. Friedland R, Macias D, Cossarini G, Daewel U, Estournel C, Garcia-Gorriz E, et al. Effects of Nutrient Management Scenarios on Marine Eutrophication Indicators: A Pan-European, Multi-Model Assessment in Support of the Marine Strategy Framework Directive. Front Mar Sci. 2021;8. doi:10.3389/fmars.2021.596126

23. Grizzetti B, Vigiak O, Udias A, Aloe A, Zanni M, Bouraoui F, et al. How EU policies could reduce nutrient pollution in European inland and coastal waters. Glob Environ Change. 2021;69: 102281. doi:10.1016/j.gloenvcha.2021.102281

24. Macias MD, Stips A, Grizzetti B, Aloe A, Bisselink B, De MA, et al. Water/marine Zero Pollution Outlook. In: JRC Publications Repository [Internet]. 28 Nov 2022 [cited 4 Jul 2023]. doi:10.2760/681817

25. Christensen V, Walters CJ. Ecopath with Ecosim: methods, capabilities and limitations. Ecol Model. 2004;172: 109–139.

26. Hyder K, Rossberg AG, Allen JI, Austen MC, Barciela RM, Bannister HJ, et al. Making modelling count - increasing the contribution of shelf-seas community and ecosystem

models to policy development and management. Mar Policy. 2015;61: 291–302. doi:10.1016/j.marpol.2015.07.015

27. de Mutsert K, Lewis K, Milroy S, Buszowski J, Steenbeek J. Using ecosystem modeling to evaluate trade-offs in coastal management: Effects of large-scale river diversions on fish and fisheries. Ecol Model. 2017;360: 14–26.

28. Serpetti N, Baudron AR, Burrows MT, Payne BL, Helaouët P, Fernandes PG, et al. Impact of ocean warming on sustainable fisheries management informs the Ecosystem Approach to Fisheries. Sci Rep. 2017;7: 13438. doi:10.1038/s41598-017-13220-7

29. Pinnegar JK, Blanchard JL, Mackinson S, Scott RD, Duplisea DE. Aggregation and removal of weak-links in food-web models: system stability and recovery from disturbance. Ecol Model. 2005;184: 229–248. doi:10.1016/j.ecolmodel.2004.09.003

30. Heymans JJ, Coll M, Link JS, Mackinson S, Steenbeek J, Walters C, et al. Best practice in Ecopath with Ecosim food-web models for ecosystem-based management. Ecol Model. 2016;331: 173–184.

31. Burek P, Roo A, Knijff J. LISFLOOD - Distributed Water Balance and Flood Simulation Model - Revised User Manual. 2013. doi:10.2788/24719

32. Grizzetti B, Bouraoui F, Aloe A. Changes of nitrogen and phosphorus loads to European seas. Glob Change Biol. 2012;18: 769–782. doi:10.1111/j.1365-2486.2011.02576.x

33. Pisoni E, Thunis P, Clappier A. Application of the SHERPA source-receptor relationships, based on the EMEP MSC-W model, for the assessment of air quality policy scenarios. Atmospheric Environ X. 2019;4: 100047. doi:10.1016/j.aeaoa.2019.100047

34. Stips A, Bolding K, Pohlmann T, Burchard H. Simulating the temporal and spatial dynamics of the North Sea using the new model GETM (general estuarine transport model). Ocean Dyn. 2004;54: 266–283. doi:10.1007/s10236-003-0077-0

35. Macias D, Stips A, Hanke G. Model based estimate of transboundary litter pollution on Mediterranean coasts. Mar Pollut Bull. 2022;175: 113121. doi:10.1016/j.marpolbul.2021.113121

36. Macias D, Huertas IE, Garcia-Gorriz E, Stips A. Non-Redfieldian dynamics driven by phytoplankton phosphate frugality explain nutrient and chlorophyll patterns in model simulations for the Mediterranean Sea. Prog Oceanogr. 2019;173: 37–50. doi:10.1016/j.pocean.2019.02.005

37. Macias D, Garcia-Gorriz E, Piroddi C, Stips A. Biogeochemical control of marine productivity in the Mediterranean Sea during the last 50 years. Glob Biogeochem Cycles. 2014;28: 897–907. doi:10.1002/2014GB004846

38. Macias D, Garcia-Gorriz E, Stips A. Deep winter convection and phytoplankton dynamics in the NW Mediterranean Sea under present climate and future (horizon 2030) scenarios. Sci Rep. 2018;8: 6626. doi:10.1038/s41598-018-24965-0

39. Macias DM, Garcia-Gorriz E, Stips A. Productivity changes in the Mediterranean Sea for the twenty-first century in response to changes in the regional atmospheric forcing. Front Mar Sci. 2015;2. doi:10.3389/fmars.2015.00079

40. Piroddi C, Coll M, Liquete C, Macias D, Greer K, Buszowski J, et al. Historical changes of the Mediterranean Sea ecosystem: modelling the role and impact of primary productivity and fisheries changes over time. Sci Rep. 2017;7. Available: ://WOS:000396126800001

41. Miladinova S, Stips A, Macias Moy D, Garcia-Gorriz E. Seasonal and Inter-Annual Variability of the Phytoplankton Dynamics in the Black Sea Inner Basin. Oceans. 2020;1: 251–273. doi:10.3390/oceans1040018

42. Daskalov GM, Demirel N, Ulman A, Georgieva Y, Zengin M. Stock dynamics and predator–prey effects of Atlantic bonito and bluefish as top predators in the Black Sea. ICES J Mar Sci. 2020;77: 2995–3005. doi:10.1093/icesjms/fsaa182

43. Mackinson S, Daskalov G. An ecosystem model of the North Sea to support an ecosystem approach to fisheries management: description and parameterisation. Cefas Sci Ser Tech Rep. 2007;142.

44. Darwall WRT, Allison EH, Turner GF, Irvine K. Lake of flies, or lake of fish? A trophic model of Lake Malawi. Ecol Model. 2010;221: 713–727. doi:10.1016/j.ecolmodel.2009.11.001

45. Link JS. Adding rigor to ecological network models by evaluating a set of pre-balance diagnostics: A plea for PREBAL. Ecol Model. 2010;221: 1580–1591.

46. Odum EP. The Strategy of Ecosystem Development. Science. 1969;164: 262–270. doi:10.1126/science.164.3877.262

47. Ulanowicz RE. Growth and Development: Ecosystems Phenomenology. New York: Springer-Verlang; 1986.

48. Christensen V, Pauly D. Flow characteristics of aquatic ecosystems. 1993. Available: https://digitalarchive.worldfishcenter.org/handle/20.500.12348/2908

49. Pauly D, Christensen V, Dalsgaard J, Froese R, Torres F. Fishing down marine food webs. Science. 1998;279: 860–863.

50. Ulanowicz R. Ascendency: A measure of ecosystem performance. 2000. pp. 303–315.

51. Christensen V, Walters CJ, Pauly D, Forrest R. Ecopath with Ecosim, version 6. User Guide. Vancouver, B.C., Canada: University of British Columbia; 2008 p. 235 pp.

52. Libralato S, Christensen V, Pauly D. A method for identifying keystone species in food web models. Ecol Model. 2006;195: 153–171. doi:10.1016/j.ecolmodel.2005.11.029

53. Mackinson S. Combined analyses reveal environmentally driven changes in the North Sea ecosystem and raise questions regarding what makes an ecosystem model's performance credible? Can J Fish Aquat Sci. 2014;71: 31–46. doi:10.1139/cjfas-2013-0173

54. Ahrens RNM, Walters CJ, Christensen V. Foraging arena theory. Fish Fish. 2012;13: 41–59. doi:10.1111/j.1467-2979.2011.00432.x

55. Holling CS. The Components of Predation as Revealed by a Study of Small-Mammal Predation of the European Pine Sawfly. Can Entomol. 1959;91: 293–320. doi:10.4039/Ent91293-5

56. Bentley WJ, Serpetti N, Heymans JJ. Investigating the potential impacts of ocean warming on the Norwegian and Barents Seas ecosystem using a time-dynamic food-web model. Ecol Model. 2017;360: 94–107.

57. Sacchi J. Analysis of economic activities in the Mediterranean: Fishery and aquaculture sectors. Valbonne; 2011.

58. Greer K, Zeller D, Woroniak J, Coulter A, Winchester M, Palomares MLD, et al. Global trends in carbon dioxide (CO2) emissions from fuel combustion in marine fisheries from 1950 to 2016. Mar Policy. 2019;107: 103382. doi:10.1016/j.marpol.2018.12.001

59. FAO. The State of Mediterranean and Black Sea Fisheries 2020. Rome, Italy: FAO; 2020. doi:10.4060/cb2429en

60. FAO. The State of Mediterranean and Black Sea Fisheries 2023. FAO ; 2023. Available: https://openknowledge.fao.org/handle/20.500.14283/cc8888en

61. Rousseau Y, Blanchard JL, Novaglio C, Pinnell KA, Tittensor DP, Watson RA, et al. A database of mapped global fishing activity 1950–2017. Sci Data. 2024;11: 48. doi:10.1038/s41597-023-02824-6

62. Damalas D, Maravelias CD, Osio GC, Maynou F, Sbrana M, Sartor P. “Once upon a Time in the Mediterranean” Long Term Trends of Mediterranean Fisheries Resources Based on Fishers’ Traditional Ecological Knowledge. PLOS ONE. 2015;10: e0119330. doi:10.1371/journal.pone.0119330

63. European Court of Auditors. Have EU measures contributed to adapting the capacity of the fishing fleets to available fishing opportunities? Publications Office of the European Union; 2011. Available: https://data.europa.eu/doi/10.2865/20007

64. Scott E, Serpetti N, Steenbeek J, Heymans JJ. A Stepwise Fitting Procedure for automated fitting of Ecopath with Ecosim models. SoftwareX. 2016;5: 25–30. doi:10.1016/j.softx.2016.02.002

65. Akaike H. New Look at Statistical-Model Identification. Ieee Trans Autom Control. 1974;Ac19: 716–723.

66. Burnham KP, Anderson DR. Multimodel Inference Understanding AIC and BIC in Model Selection. Sociol Methods Res. 2004;33: 261–304. doi:10.1177/0049124104268644

67. Bentley JW, Lundy MG, Howell D, Beggs SE, Bundy A, de Castro F, et al. Refining Fisheries Advice With Stock-Specific Ecosystem Information. Front Mar Sci. 2021;8: 346. doi:10.3389/fmars.2021.602072

68. Bentley JW, Chagaris D, Coll M, Heymans JJ, Serpetti N, Walters CJ, et al. Calibrating ecosystem models to support ecosystem-based management of marine systems. ICES J Mar Sci. 2024;81: 260–275. doi:10.1093/icesjms/fsad213

69. R Core Team. R: A Language and Environment for Statistical Computing. Vienna, Austria: R Foundation for Statistical Computing; 2023.

70. Bronaugh D, Werner A. Zyp: Zhang + Yue-Pilon Trends Package. R package. 2023.

71. Zhang X, Vincent LA, Hogg WD, Niitsoo A. Temperature and precipitation trends in Canada during the 20th century. Atmosphere-Ocean. 2000;38: 395–429. doi:10.1080/07055900.2000.9649654

72. de Mutsert K, Coll M, Steenbeek J, Ainsworth C, Buszowski J, Chagaris D, et al. Advances in spatial-temporal coastal and marine ecosystem modeling using Ecospace. Reference Module in Earth Systems and Environmental Sciences. Elsevier; 2023. doi:10.1016/B978-0-323-90798-9.00035-4

73. Shurova NM. Influence of Salinity on the Structure and the State of Bivalve Mytilus galloprovincialisPopulations. Russ J Mar Biol. 2001;27: 151–155. doi:10.1023/A:1016713401707

74. Christensen V, Coll M, Steenbeek J, Buszowski J, Chagaris D, Walters CJ. Representing Variable Habitat Quality in a Spatial Food Web Model. Ecosystems. 2014;17: 1397–1412.

75. Kaschner K, Kesner-Reyes K, Garilao C, Rius-Barile J, Rees T, Froese R. AquaMaps: Predicted range maps for aquatic species. AquaMaps: Predicted range maps for aquatic species. World wide web electronic publication, www.aquamaps.org; 2019.

76. Piroddi C, Coll M, Steenbeek J, Moy DM, Christensen V. Modelling the Mediterranean marine ecosystem as a whole: addressing the challenge of complexity. Mar Ecol Prog Ser. 2015;533: 47–65. doi:10.3354/meps11387

77. Heymans JJ, Coll M, Libralato S, Morissette L, Christensen V. Global Patterns in Ecological Indicators of Marine Food Webs: A Modelling Approach. PLOS ONE. 2014;9: e95845. doi:10.1371/journal.pone.0095845

78. Costanza R, Mageau M. What is a healthy ecosystem? Aquat Ecol. 1999;33: 105–115. doi:10.1023/A:1009930313242

79. Power ME, Tilman D, Estes JA, Menge BA, Bond WJ, Mills LS, et al. Challenges in the Quest for Keystones: Identifying keystone species is difficult—but essential to understanding how loss of species will affect ecosystems. BioScience. 1996;46: 609–620. doi:10.2307/1312990

80. Daskalov G, Cardinale M, Gumus A, Panayotova M, Duzgunes E, Shlyakhov V, et al. Scientific, Technical and Economic Committee for Fisheries (STECF) Opinion by written procedure Assessment of Black Sea Stocks (STECF-OWP-11-06) Edited by Georgi Daskalov and Hans-Joachim Rätz. 2011 Dec. doi:10.2788/94711

81. European Union. Regulation 2016/73 - Fixing for 2016 of the fishing opportunities for certain fish stocks in the Black Sea - EU monitor. Sect. L 16/1, Official Journal of the European Union 2016. Available: https://www.eumonitor.eu/9353000/1/j4nvk6yhcbpeywk_j9vvik7m1c3gyxp/vk11b65xd9zi

82. Sánchez-Cabanes A, Nimak-Wood M, Harris N, Stephanis R de. Habitat preferences among three top predators inhabiting a degraded ecosystem, the Black Sea. Sci Mar. 2017;81: 217–227. doi:10.3989/scimar.04493.07A

83. ACCOBAMS, Paiu R-M, Panigada S, Cañadas A, Gol'din P, Popov D, et al. Estimates of abundance and distribution of cetaceans in the Black Sea from 2019 surveys. Monaco: ACCOBAMS; 2021 p. 54 pages.

84. Yu.P Z, Alexandrov B, Berlinsky NA, Zenetos A. Zaitsev Yu.P., B.G. Alexandrov, N.A. Berlinsky, & A. Zenetos, 2002. The Black Sea an oxygen-poor sea. In: Europe's biodiversity - biogeographical regions and seas. Environmental issue report Published by EEA (European Environment Agency) Copenhagen 2002 (http://reports.eea.eu.int/report_2002_0524_154909/en). 2002.

85. Miauta N. Report on the implementation of the memorandum of understanding regarding the conservation of migratory shark spiny dogfish in the Black Sea. Romania; 2012. Available: https://www.mmediu.ro/img/attachment/32/biodiversitate-54784ff997265.pdf

86. Oguz T, Dippner JW, Kaymaz Z. Climatic regulation of the Black Sea hydro-meteorological and ecological properties at interannual-to-decadal time scales. J Mar Syst. 2006;60: 235–254. doi:10.1016/j.jmarsys.2005.11.011

87. Dischereit A, Beermann J, Lebreton B, Wangensteen OS, Neuhaus S, Havermans C. DNA metabarcoding reveals a diverse, omnivorous diet of Arctic amphipods during the polar night, with jellyfish and fish as major prey. Front Mar Sci. 2024;11. doi:10.3389/fmars.2024.1327650

88. Mann KH. Production and use of detritus in various freshwater, estuarine, and coastal marine ecosystems. Limnol Oceanogr. 1988;33: 910–930. doi:10.4319/lo.1988.33.4part2.0910

89. Azam F, Fenchel T, Field J, Gray J, Meyer-Reil L, Thingstad F. The Ecological Role of Water-Column Microbes in the Sea. Mar Ecol Prog Ser. 1983;10: 257–263. doi:10.3354/meps010257

Figure 1

Click here to access/download;Figure;Fig.1 Ecospace_Env.tif

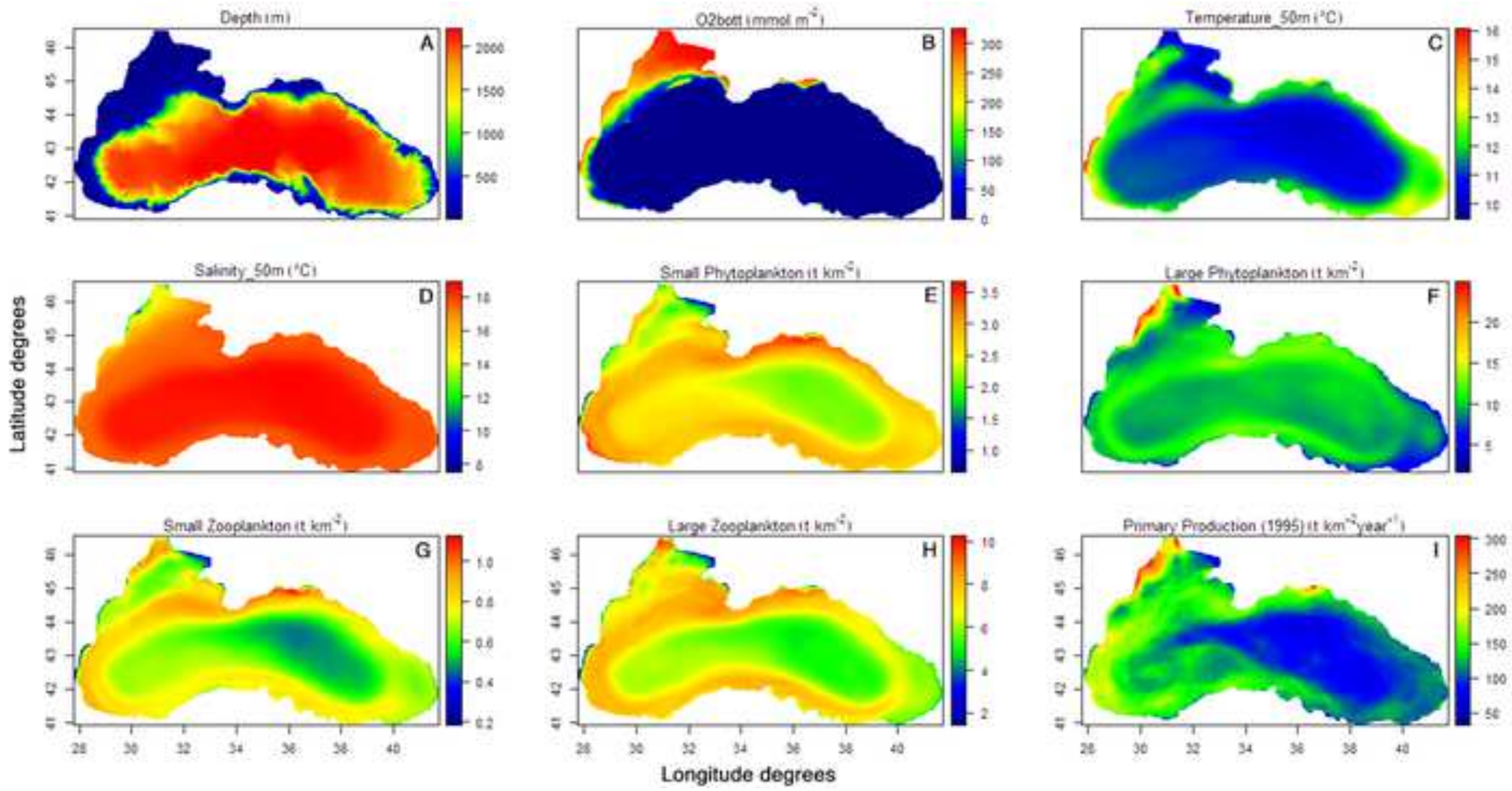

Figure 2

Click here to access/download;Figure;Fig.2 Flow diagram.tif

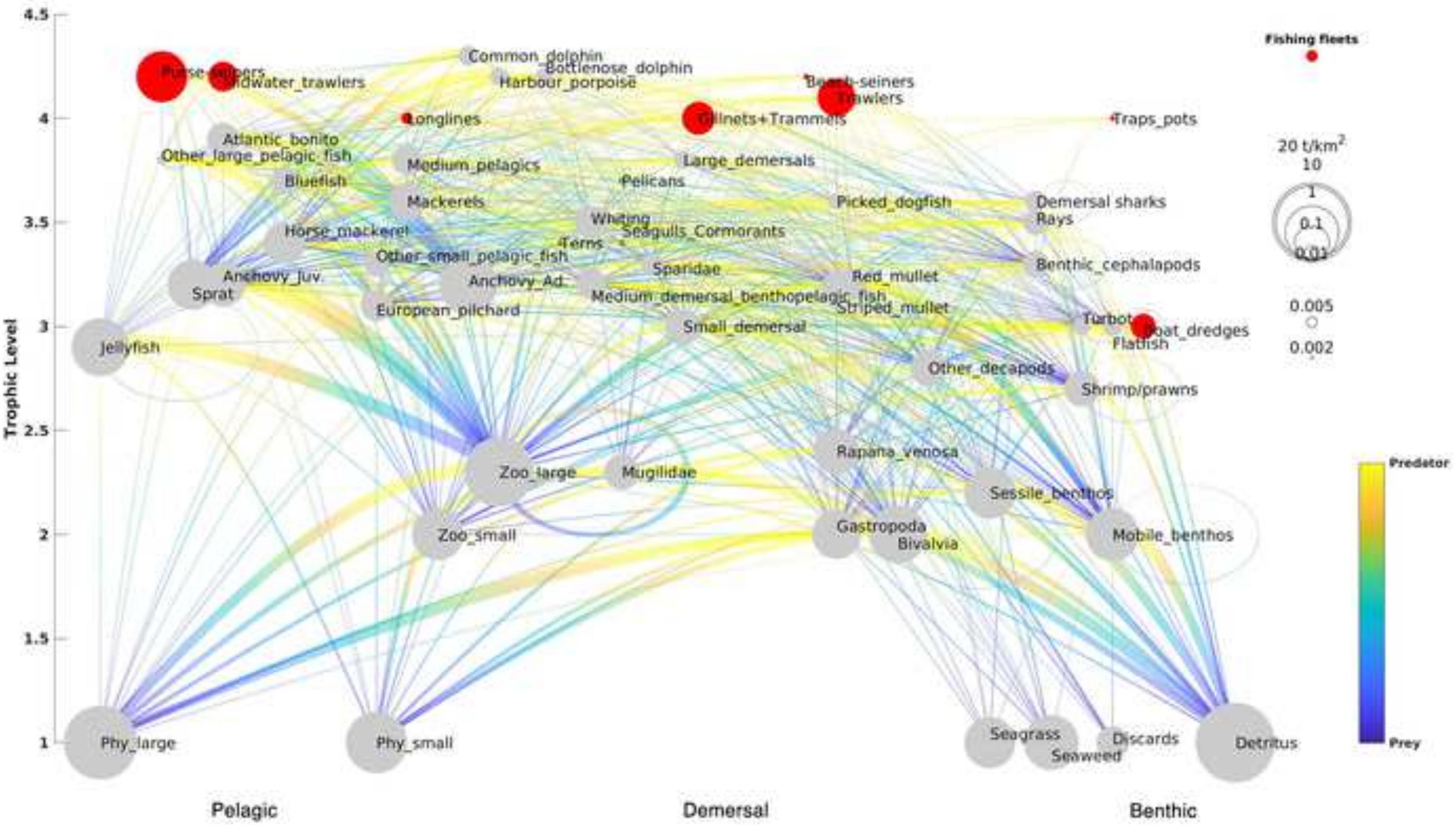

Click here to access/download;Figure;Fig.3 MTI.tif

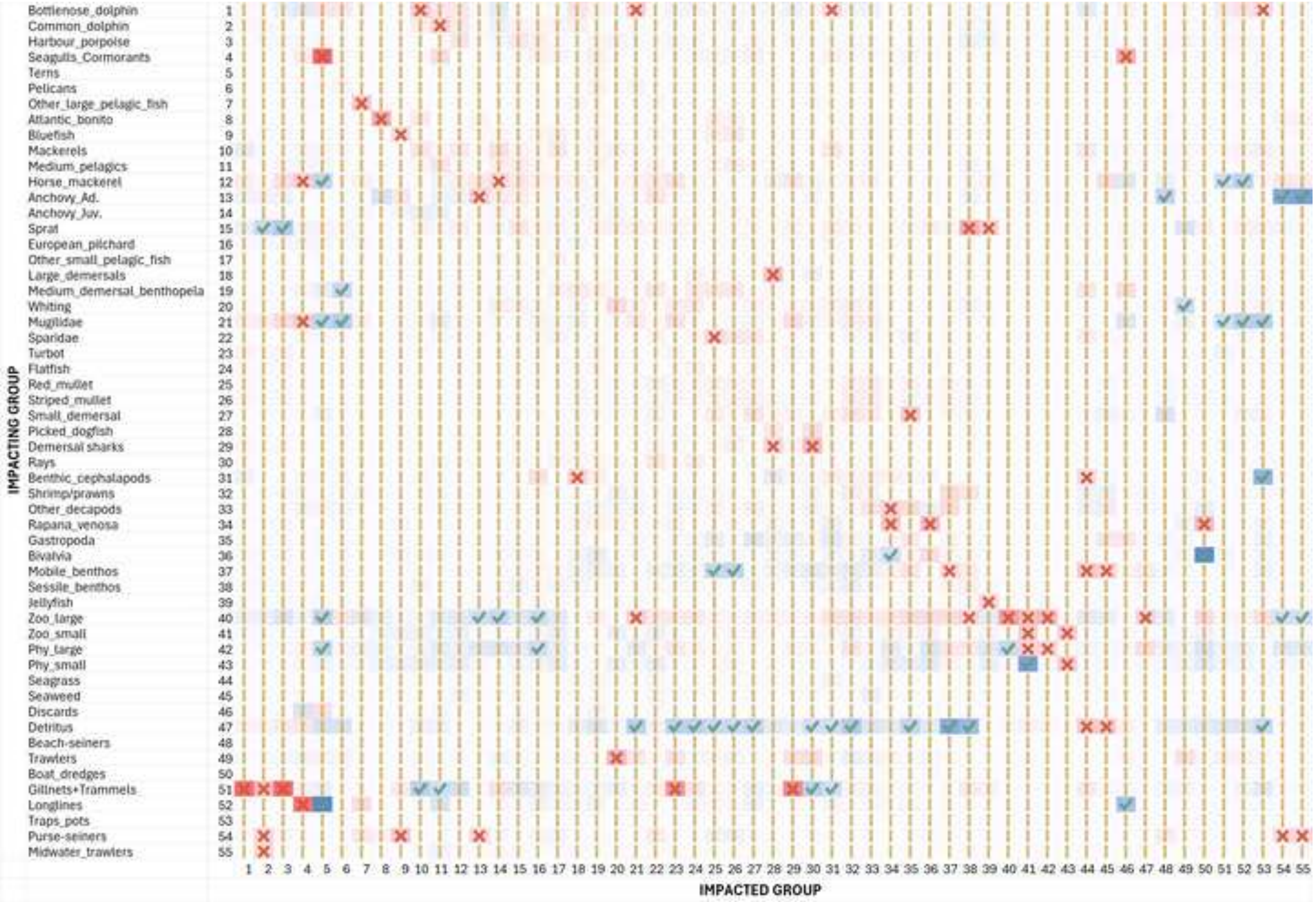

Figure 4

Click here to access/download;Figure;Fig.4 Keystoneness.tif

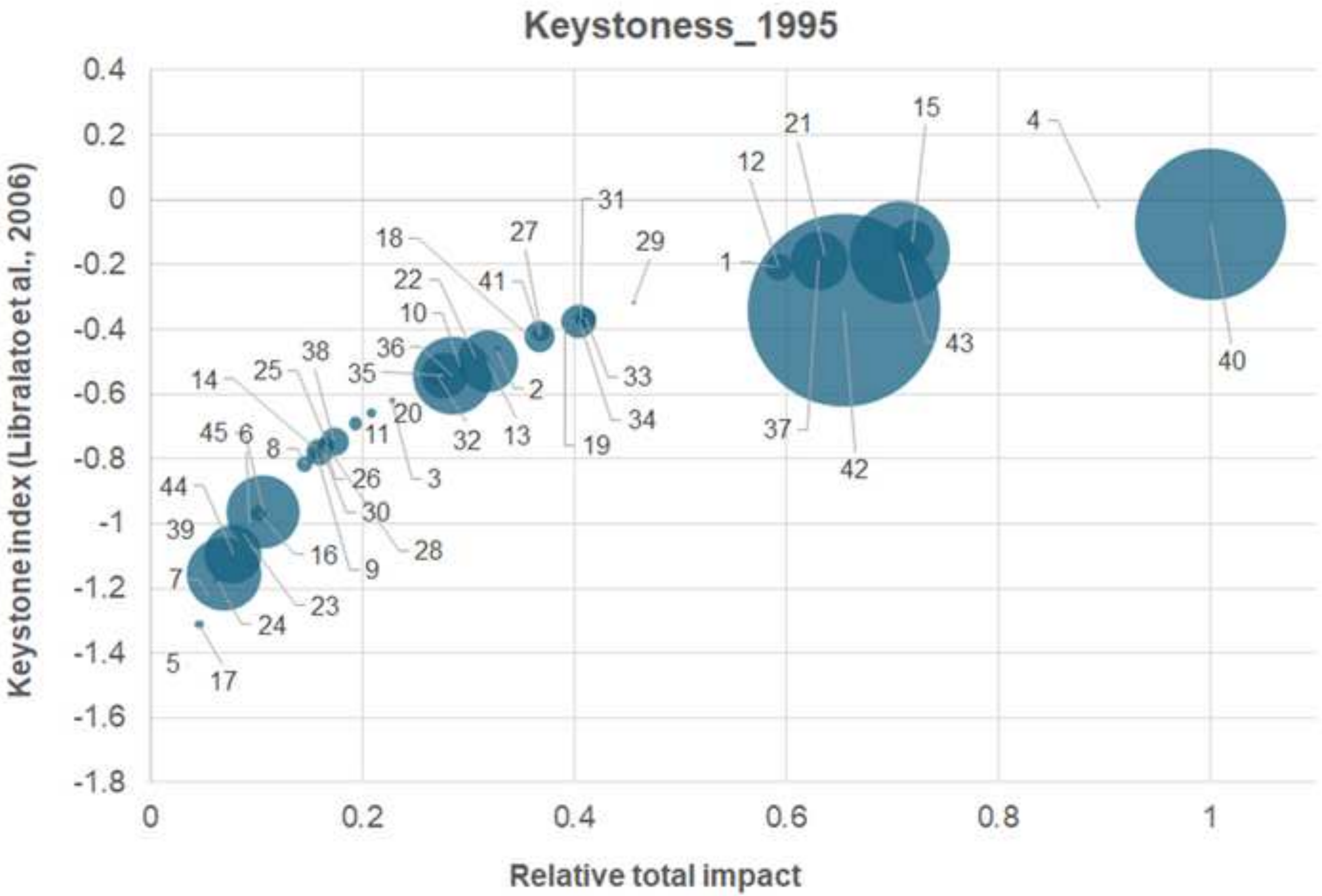

Figure 5

Click here to access/download;Figure;Fig.5 Fitted_trends.tif

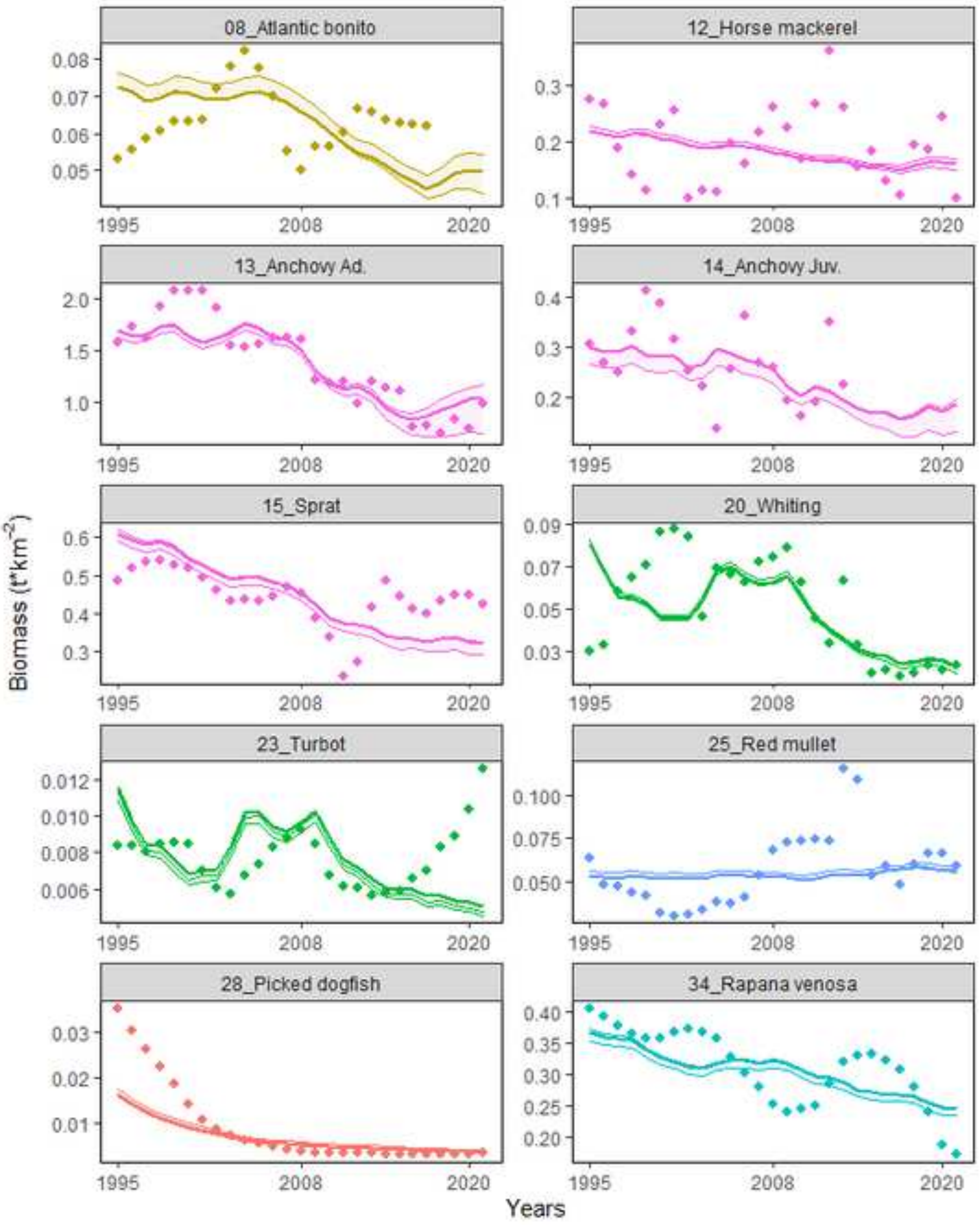

Figure 6

Click here to access/download;Figure;Fig.6 Stock_assessed.tiff

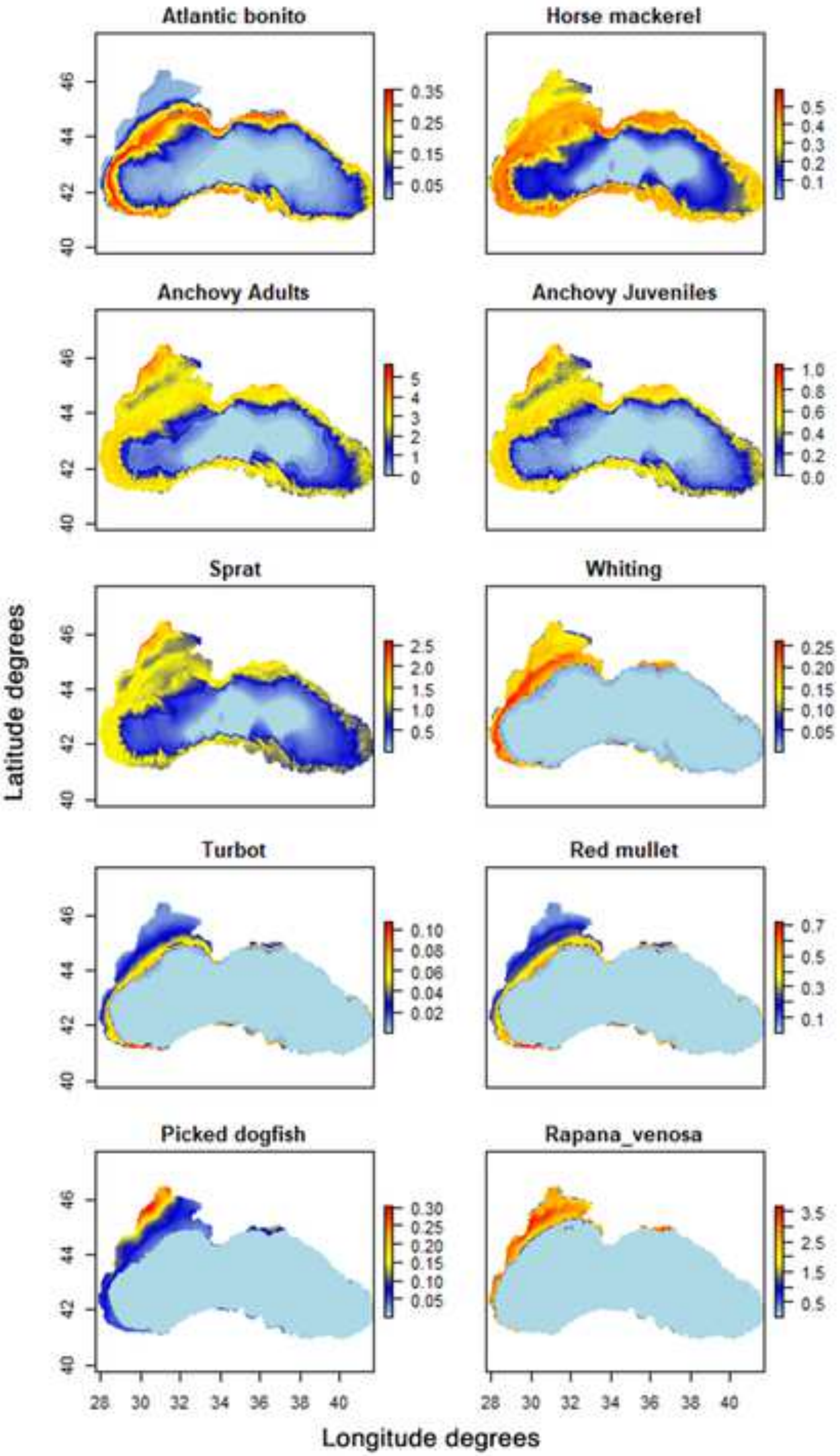

Figure 7

Click here to access/download;Figure;Fig.7 Habitat vs suitability.tif

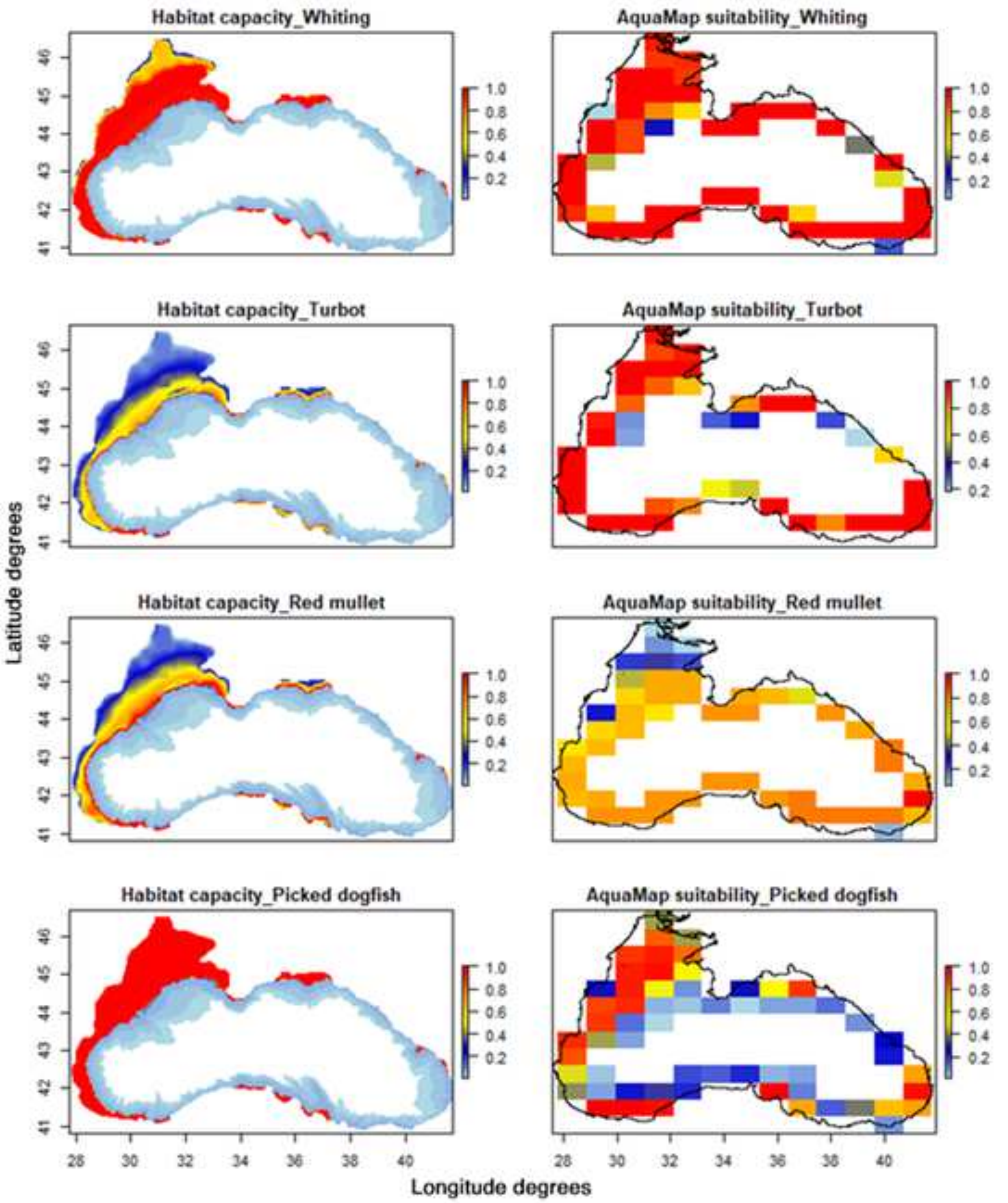

Click here to access/download
Supporting Information
Appendix 1.docx

Click here to access/download
Supporting Information
Appendix 2.xlsx
