# Supplementary material for: State of the art modelling for the Black Sea ecosystem to support European policies
Source: PLoS One. 2025 Jan 15;20(1):e0312170. doi: 10.1371/journal.pone.0312170 (PMC11734964; doi:10.1371/journal.pone.0312170)
Supplement: Attachment — Submitted filename: Responses to Reviewers_final.docx [file pone.0312170.s004.docx]

**Responses to Reviewers**

We sincerely thank the reviewers for their detailed feedback on our paper, which has greatly enhanced its quality. We found the reviewers’ comments to be incredibly helpful, particularly regarding the technical aspects of our modelling approach and insights into the Black Sea ecosystem.

We have carefully followed all the suggestions and have made the necessary revisions to the manuscript accordingly.

Best Regards,

Natalia Serpetti and co-Authors.

**Reviewer 1 Taner Yildiz**

Reviewer #1: Thank you for the opportunity to review this insightful manuscript titled "The state of the art modelling for the Black Sea ecosystem to support European policies." The study presents a significant contribution to our understanding of the Black Sea ecosystem, particularly in the context of supporting European policy frameworks. The methodology is rigorous, and the findings are both relevant and timely.

While the manuscript is well-structured and the content is compelling, I have identified a few minor issues that could be addressed to further enhance the clarity and impact of the paper. These minor suggestions are detailed below.

The species mentioned in the introduction should be supported with their scientific names, as was done with *Mnemiopsis leidyi*. While I understand that these are provided in the appendices, not all readers may have the same access to the full text or appendices, leading to potential misunderstandings.

*The scientific names of the species mentioned in the introduction were added (lines 44-55).*

In most of the sentences referenced in the introduction, a single reference is used, which weakens the strength of the literature support.

*More references have been added in the introduction (lines 38-41).*

Line 44-45: The authors mention 'recent reports,' implying multiple sources; however, only a single reference is provided.

*Another reference was added (line 49).*

I would not describe the Black Sea as a 'hotspot' of biodiversity, as stated in lines 56-57. While there is certainly biodiversity present in the Black Sea, I do not agree that it qualifies as a hotspot compared to other seas.

*This sentence was removed from the introduction and the reference was used in the discussion in relation to the biodiversity results.*

In lines 60 to 76, in addition to mentioning the contributions of the European Commission and its science Directorate-General (DG) and the Joint Research Centre, it would be beneficial to also highlight the efforts of the GFCM in bringing countries to the table and in data collection. These efforts are particularly relevant, given that the publication utilizes significant data from these initiatives.

*This aspect was added in the introduction as advised (lines 71-73).*

Lines 435-443 should be supported by existing literature. It is important to verify whether the 'assumptions' familiar in other seas also hold true for the Black Sea. For example, it is well-documented that interactions between dolphins and gillnets & trammels are frequent in the Black Sea due to declining fish stocks, which increases encounters between fishermen and dolphins. However, I have not come across any publications regarding the impact of trawlers on seagulls in the Black Sea. If such references exist, they should be cited here.

*We thank the reviewer for highlighting this point. The references were added for gillnets and trammel and for longliners. Trawlers have a positive interaction of seagulls as they feed on discards (lines 467-473). Unfortunately, we haven’t found a specific reference for this phenomenon in the Black Sea, but, as it is a well-known event, we provided references for other part of the world.*

In lines 491-503, it would be important to mention the quota system recommended and implemented by the GFCM to protect the remaining turbot stocks. Member countries of the GFCM are adhering to this regulation. The relevant text is as follows:

In lines 481-490, it should be noted that the similarities between Mullus barbatus (red mullet) and Mullus surmuletus (striped red mullet) often lead to inaccuracies in species reporting by fishermen. For instance, in Turkey, the minimum landing size for Mullus barbatus is 13 cm, while for Mullus surmuletus it is 11 cm. To avoid penalties and to land more catch, fishermen may register Mullus barbatus as Mullus surmuletus. This practice raises concerns about the accuracy of catch statistics for these two species.

*The paragraph was extended including these points including a reference of species misreporting (lines 531-537).*

'In the Black Sea, the proposal includes catch limits and quotas for sprat and turbot. For sprat, the Commission proposes to maintain the 2023 catch limit. For turbot, the levels of total allowable catches (TAC) and quotas will be set and adopted at the 2023 GFCM annual session. The proposal demonstrates the Commission's objective to make fisheries in these two sea basins sustainable, in line with the 2030 Strategy of the General Fisheries Commission for the Mediterranean (GFCM), which is the regional fisheries management organization competent for the conservation and management of fish stocks in the Mediterranean and the Black Seas.' (<https://ec.europa.eu/commission/presscorner/detail/en/ip_23_4861>)

*We thank the reviewer for these valid discussion points that have been included (lines 552-555).*

This addition would emphasize the ongoing efforts to manage and conserve key fish stocks in the Black Sea.

The manuscript's contribution to supporting European policies should be elaborated further in the discussion section, particularly given its mention in the title. Expanding on how the findings align with or inform key European policy objectives would strengthen the manuscript's relevance. Specifically, discussing how the research supports the 2030 Strategy of the General Fisheries Commission for the Mediterranean (GFCM) and other EU sustainability goals would provide valuable context. This could include an analysis of how the data contributes to decision-making processes at the European level. Such a discussion would emphasize the manuscript’s practical implications for policy development and implementation.

*The paragraph was extended including these points but in the conclusion rather than the discussion as it was suggested also by reviewer 2*

**Reviewer 2**

Reviewer #2: This study builds on previous EwE models of the Black Sea ecosystem, adding more refined data where possible, and develops the first spatially and temporally explicit Ecospace model for the region. Spatial results are qualitatively compared to other published and modeled species distributions for verification purposes in the absence of detailed spatial data for many Black Sea species. The developed Ecospace is expected to be the baseline for future testing of spatial management scenarios in support of and potential revision of the MSFD. I find the study to be rigorous and well documented, with a detailed supplement. I suggest that the authors do some mostly minor revisions considering the comments I have made on the attached PDF.

- Comment line 27 “successfully based on what? observed data? expert knowledge?”

*“Observed data”, this was added in the text.*

- Comment line 150 “perhaps this could be further explained”.

*Biomass accumulation was explained and a new reference added (lines 152-157 new version).*

- Comment line 174 “would be informative to list them here”.

*The balancing criteria were listed with also the reference to the Appendix.*

- Comment line 331 “spin-up” what does this mean?

*Explanation and reference were added.*

- Comment line 344 “integrated”, “what does this mean practically? Is it the same as 'averaged'?

*No, for integration we meant the SUM of the small phytoplankton for each depth layer up to 50 meters to account for the total biomass of small phytoplankton in the photic zone.*

- Comment line 362 Figure 2 “it's interesting to see visualized that most fishing fleets are top predators. you could perhaps add as a discussion point.

*This point was added in the discussion.*

- Comment line 394 “is this wording right? I find it hard to understand”.

*This has been rephrased. (lines 408-409 new version).*

- Comment line 413 “system ascendency, would be useful to elaborate a bit more”.

*This sentence was rephrased.*

- Comment line 427 “it's better to define all acronyms in the legend or below the table”.

*This has been added in the legend.*

- Comment line 445. “Based on Libralato et al. 2006 keystone groups are the one that have an index close to or greater than zero. Keramidas et al. 2022 define as true keystone group the one that has an index greater than zero. <https://www.sciencedirect.com/science/article/abs/pii/S0304380005006149>

*Thank you for highlighting this paper. We were not aware of this definition. However, we do not find useful to use this definition to assess changes of keystoness control on the Black Sea food web because, in our findings, all groups showed values of the index below zero similar to previous Black Sea models.*

- Comment line 462. “Names better be added on the figure which should be read without one needing to cross reference another table. Some names can go next to each numbers and some can be given in a list that can be inserted at one corner of the graph”.

*The legend of the name was added to the figure.*

- Comment line 468. “no, the fit for horse mackerel or red mullet is not good at all. the model is not capturing the observed fluctuations.

*This sentence was corrected*.

- Comment line 481 and 485. “when reporting the results and discussing them, the authors could differentiate between "fit of the model to the observed fluctuations" and "the model capturing the general increasing or decreasing trend in the observations" & “no, Table S4 reports only negative slopes, including the red mullet”.

*The paragraph was changed accordantly to the suggestions, sum of squares were added in the text and explained in the methods.*

- Comment line 489, “again, this maybe can be seen in Fig. 5 (it's very slight to assess visually), but it's not what is reported in Table S4”.

*The sentence was removed as it was incorrect.*

- Comment line 493, 498, 503. *All the suggestions were taken into account and the text changed accordantly.*
- Comment line 507, 509, 515. *All the suggestions were taken into account and the text changed accordantly.*
- Comment line 521, 524. *All the suggestions were taken into account and the text changed accordantly.*
- Comments line 529-533. *All the suggestions were taken into account and the text changed accordantly.*
- Comments line 534. “habitat capacity: could you please explain this?”. *Habitat capacity is explained in lines 347-351 (in this revised version). We re-named “capacity” with “Habitat Foraging Capacity” to remind this to the readers.*
- Comment line 536. “Turbot map is the least matching one. Any idea why?”.

*Thank you very much to the reviewer for highlighted this point. We found an error on depth niche for turbot and the Ecospace model was re-run and the figures 6 and 7 were corrected.*

- Comments line 540. “how does the highest biodiversity in the northwestern shelf imply fragility of the system?”

*It highlights the fragile status of the region as it is confined. We removed this confusing sentence.*

- Comments line 549. “Generally conclusions are meant to be more focused on the outcomes of this work, rather than an introduction-like summary of other references. I suggest modifying the first paragraph accordingly. The second paragraph seems more fitting.

*The text was changed accordantly to the suggestions.*

- Comment line 650. “Change the paper “Christensen & Walters 2004” with the new book.

*We added the new book “Christensen & Walters 2024” but we kept also the old reference as it is the most representative citation of the EwE software and highlights that the approach has been used since a long time.*
